# Stress ulcer prophylaxis with proton pump inhibitors or histamine 2 receptor antagonists in critically ill adults - a meta-analysis of randomized controlled trials with trial sequential analysis

**DOI:** 10.1186/s12876-019-1105-y

**Published:** 2019-11-21

**Authors:** Xiaoyang Zhou, Hanyuan Fang, Jianfei Xu, Peifu Chen, Xujun Hu, Bixin Chen, Hua Wang, Caibao Hu, Zhaojun Xu

**Affiliations:** 1 Department of Intensive Care Medicine, HwaMei Hospital, University Of Chinese Academy of Sciences, Ningbo, 315000 Zhejiang China; 2 Department of Emergency, Ningbo Yinzhou No.2 Hospital, Ningbo, 315000 Zhejiang China; 3 Department of Intensive Care Medicine, Zhejiang Hospital, Hangzhou, 310000 Zhejiang China

**Keywords:** Stress ulcer, Proton pump inhibitors, Histamine 2 receptor antagonists, Critically ill, Gastrointestinal bleeding

## Abstract

**Background:**

Proton pump inhibitors (PPI) and histamine 2 receptor antagonists (H2RA) have been widely used as stress ulcer prophylaxis (SUP) in critically ill patients, however, its efficacy and safety remain unclear. This study aimed to assess the effect of SUP on clinical outcomes in critically ill adults.

**Methods:**

Literature search was conducted in PubMed, EMBASE, Web of Science, and the Cochrane database of clinical trials for randomized controlled trials (RCTs) that investigated SUP, with PPI or H2RA, versus placebo or no prophylaxis in critically ill patients from database inception through 1 June 2019. Study selection, data extraction and quality assessment were performed in duplicate. The primary outcomes were clinically important gastrointestinal (GI) bleeding and overt GI bleeding. Conventional meta-analysis with random-effects model and trial sequential analysis (TSA) were performed.

**Results:**

Twenty-nine RCTs were identified, of which four RCTs were judged as low risk of bias. Overall, SUP could reduce the incident of clinically important GI bleeding [relative risk (RR) = 0.58; 95% confidence intervals (CI): 0.42–0.81] and overt GI bleeding (RR = 0.48; 95% CI: 0.36–0.63), these results were confirmed by the sub-analysis of trials with low risk of bias, TSA indicated a firm evidence on its beneficial effects on the overt GI bleeding (TSA-adjusted CI: 0.31–0.75), but lack of sufficient evidence on the clinically important GI bleeding (TSA-adjusted CI: 0.23–1.51). Among patients who received enteral nutrition (EN), SUP was associated with a decreased risk of clinically important GI bleeding (RR = 0.61; 95% CI: 0.44–0.85; TSA-adjusted CI: 0.16–2.38) and overt GI bleeding (RR = 0.64; 95% CI: 0.42–0.96; TSA-adjusted CI: 0.12–3.35), but these benefits disappeared after adjustment with TSA. Among patients who did not receive EN, SUP had only benefits in reducing the risk of overt GI bleeding (RR = 0.37; 95% CI: 0.25–0.55; TSA-adjusted CI: 0.22–0.63), but not the clinically important GI bleeding (RR = 0.27; 95% CI: 0.04–2.09).

**Conclusions:**

SUP has benefits on the overt GI bleeding in critically ill patients who did not receive EN, however, its benefits on clinically important GI bleeding still needs more evidence to confirm.

## Background

Critically ill patients admitted to the intensive care unit (ICU) are often exposed to the potential risk factors for stress-related gastrointestinal (GI) bleeding, which includes mechanical ventilation, coagulopathy, renal replacement therapy, etc. [1, 2]. Thus, critically ill patients are always at high risk of stress-related GI bleeding. However, the incidence of stress-related GI bleeding in ICU patients has decreased notably to approximately 2–5% [2, 3] over the past decade, which is due, at least in part, to improved hemodynamic management and better control of mechanical ventilation (MV) [4]. Although, critically ill patients complicated with GI bleeding are still associated with higher mortality and worse clinical outcomes [5, 6], a multi-center cohort study recorded a 90-day mortality of more than 50% in ICU patients complicated with clinically important GI bleeding [2]. Hence, it is important and necessary for ICU patients to take pharmacy prophylaxis for stress-related GI bleeding.

Proton pump inhibitors (PPI) and histamine2 receptor antagonists (H2RA) have been used as stress ulcer prophylaxis (SUP) in critically ill patients for more than 40 years [7], however, its efficacy and safety remain controversial. Over the past decades, numerous randomized controlled trials (RCTs) [7–13] were conducted to investigate the relationship of SUP and clinical outcomes in critically ill patients, several meta-analyses [14–16] found that SUP with PPI or H2RA was effective in preventing GI bleeding compared with placebo or no prophylaxis, however, a recent meta-analysis by Huang et al. [17] concluded that SUP with PPI or H2RA had no beneficial effects on GI bleeding in ICU patients receiving enteral nutrition (EN). Meanwhile, some studies [14, 17] revealed an increased risk of pneumonia with use of PPI or H2RA, but others studies [15, 16] suggested that SUP with PPI or H2RA was not accompanied by increased incident of pneumonia. We are confused about the benefits and harms of SUP with PPI or H2RA in critically ill patients in consideration of the above contradictory results. Recently, several RCTs, the SUP-ICU trial [18] in particular, were published, these studies will expand the sample size and may make a firm conclusion on the efficacy and safety of SUP in critically ill patients. For that reason, we conducted a new meta-analysis of RCTs with trial sequential analysis to detect the effect of SUP, with PPI or H2RA, on clinical outcomes in critically ill patients. Also, the difference of the outcome effects between patients who received EN and those did not receive EN will be investigated.

## Methods

### Search strategy

This meta-analysis was performed in accordance with the PRISMA guidance [19]. Literature search with medical subject heading terms and liberal terms was performed by two independent authors (Zhou X and Fang H) for RCTs of adult critically ill patients, where SUP with PPI or H2RA was compared to placebo or no prophylaxis, in PubMed, EMBASE, Web of Science and the Cochrane database of clinical trials from database inception through 5 November 2018, the last database search was updated on 1 June 2019. We also reviewed articles from others meta-analyses to avoid missing relevant literature. There was no restriction on language in this meta-analysis. The detailed search strategy is shown in the Additional file 1.

### Study selection

Firstly, all searched records were filtered to exclude duplicates. Then, three authors (Xu J, Chen P, and Hu X) independently screened the titles and abstracts of the remaining records to determine whether the studies met the inclusion criteria. The eligible articles were included in a further review of the full-text in accordance with the inclusion and exclusion criteria. The inclusion criteria in our meta-analysis include: 1) prospective RCTs where the interventions group received SUP with PPI or H2RA, with no restrictions on dosage, duration of drug used, or drug-delivery methods, and the control group received placebo or no prophylaxis; 2) adults critically ill patients (age ≥ 18 years) admitted to the ICU. The primary outcomes were clinically important GI bleeding and overt GI bleeding, the secondary outcomes includes all-cause mortality, incident of pneumonia and *Clostridium difficile* infection (CDI), and the tertiary outcomes were duration of MV and ICU stay. Studies that reported at least one of the above outcomes were included in this meta-analysis. However, studies that enrolled critically ill patients who were not admitted to the ICU were excluded. The different opinions between the three authors were discussed to reach a consensus.

### Data extraction

The associated data were extracted independently by two reviewers (Chen B and Wang H) using a standardized data extraction protocol, including characteristics of the included studies, details of the population enrolled, detailed information on the interventions including the type of SUP, dose, and regimen. The interested clinical outcomes were also recorded. In studies in which various mortalities were reported, the longest follow-up all-cause mortality was included in analysis. We predefined overt GI bleeding as evidence of hematemesis, melena, hematochezia, or coffee-grounds emesis or aspirate regardless of other clinical findings, clinically important GI bleeding was defined as evidence of GI bleeding plus any of the following signs: significant hemodynamic changes not explained by other causes, need for transfusion of blood, significant decrease in hemoglobin level, or need for surgery to control the bleeding [14]. Discrepancies between reviewers were resolved through discussion.

### Quality assessment

The risk of bias of each included studies were assessed by two independent authors (Hu C and Xu Z) for the primary outcomes (clinically important GI bleeding and overt GI bleeding) in the following six domains: adequate sequence generation, allocation concealment, blinding of participants and personnel, blinding of outcome assessment, incomplete outcome data, selective reporting, and other bias [20, 21]. Only studies that exhibited a low risk of bias in all domains were judged as low risk of bias. We resolved the disagreements by discussing with a third reviewer until a consensus.

### Statistical analysis

Data analyses were conducted using Stata/SE 11.0 (StataCorp, College Station, TX, USA). We calculated relative risk (RR) for dichotomous data and mean differences (MD) for continuous data, with 95% corresponding confidence interval (CI). In studies with multiple intervention groups received different type of SUP (such as one group received PPI, and another group received H2RA), the number of events and total subjects in the control group would be divided approximately evenly into multiple groups to make multiple comparisons, the means and SD were left unchanged, if the intervention groups received same type of SUP, the multiple groups would be combined into a single group, and the means and SD can be combined using methods described in Cochrane handbook (Chapter 7) [20].

Statistical heterogeneity among trials was evaluated by inspecting the Forest plots and quantified using the diversity (D^2^) and inconsistency factor (I^2^) statistics. Both fixed-effects and random-effects model were used to calculate the pooled results for all outcomes (Table 1). Given the potential clinical and statistical heterogeneity (in the type of SUP, drug-delivery methods, and ICU setting, etc.) between included trials, we reported the random-effects pooled data as the main results [22]. Subgroup analyses were also conducted for all outcomes based on the overall risk of bias of included studies (low or high risk of bias), the type of SUP (PPI or H2RA), and whether EN was received (yes or no). Funnel plots combined with Begg’s and Egger’s tests were performed to assess publication bias for the primary outcomes if the number of included trials was more than 10. Considering that the primary and secondary outcomes were multiple, we assessed the primary outcomes with statistical significance set at a *P* value of 0.033 or less, the secondary outcomes at a *P* value of 0.025 or less, and the tertiary outcomes at a *P* value of 0.33 or less [22].

**Table 1:** The conventional meta-analysis and trial sequential analysis using random-effects and fixed-effects for all outcomes

	Conventional meta-analysis				Trial sequential analysis (TSA)					
	Random-effects		Fixed-effects						Incidence in control group	Required information size
	RR or WMD (95% CI)	*P*	RR or WMD (95% CI)	*P*	I^2^	Random-effect TSA-adjusted 95% CI	Fixed-effect TSA-adjusted 95% CI	Actual diversity (D^2^)		
Primary endpoints										
*Clinically important GI bleeding*										
All trials (11 trials)	0.58 (0.42–0.81)	0.001	0.56 (0.41–0.76)	< 0.001	0%	0.23–1.51	0.22–1.39	0%	4.6%	22,114
Low risk of bias (4 trials)	0.64 (0.45–0.92)	0.017	0.64 (0.45–0.92)	0.016	0%	0.15–2.80	0.15–2.77	0%	4.1%	24,928
High risk of bias (7 trials)	0.38 (0.17–0.84)	0.017	0.35 (0.18–0.67)	0.002	11.8%	0.01–9.93	0.03–4.93	19%	6.7%	13,827
Received PPI (6 trials)	0.61 (0.43–0.88)	0.008	0.61 (0.43–0.88)	0.008	0%	0.14–2.70	0.14–2.68	0%	3.8%	26,970
Received H2RA (6 trials)	0.45 (0.17–1.22)	0.116	0.42 (0.23–0.78)	0.006	42.6%	–	–	50%	10.4%	–
Received EN (8 trials)	0.61 (0.44–0.85)	0.004	0.61 (0.44–0.84)	0.003	0%	0.16–2.38	0.16–2.33	0%	4.2%	24,310
Did not receive EN (3 trials)	0.27 (0.04–2.09)	0.211	0.27 (0.10–0.73)	0.010	62.3%	–	–	65%	13.0%	–
*Overt GI bleeding*										
All trials (27 trials)	0.48 (0.36–0.63)	< 0.001	0.50 (0.42–0.59)	< 0.001	34.6%	0.31–0.75	0.38–0.65	62%	12.1%	15,468
Low risk of bias (4 trials)	0.62 (0.48–0.79)	< 0.001	0.62 (0.49–0.79)	< 0.001	0%	0.37–1.04	0.37–1.04	0%	8.6%	11,384
High risk of bias (23 trials)	0.42 (0.30–0.60)	< 0.001	0.40 (0.32–0.51)	< 0.001	37.4%	0.25–0.72	0.28–0.57	52%	18.1%	7595
Received PPI (8 trials)	0.57 (0.45–0.71)	< 0.001	0.57 (0.45–0.71)	< 0.001	0%	0.36–0.88	0.36–0.88	0%	8.9%	10,969
Received H2RA (22 trials)	0.45 (0.30–0.67)	< 0.001	0.43 (0.33–0.54)	< 0.001	45.7%	0.26–0.79	0.30–0.60	59%	19.3%	8258
Received EN (13 trials)	0.64 (0.42–0.96)	0.029	0.60 (0.48–0.74)	< 0.001	25.7%	0.12–3.35	0.25–1.42	71%	9.2%	27,681
Did not receive EN (14 trials)	0.37 (0.25–0.55)	< 0.001	0.36 (0.27–0.48)	< 0.001	38.4%	0.22–0.63	0.25–0.52	52%	21.9%	6029
Secondary endpoints										
*All-cause mortality*										
All trials (24 trials)	1.01 (0.93–1.09)	0.842	1.02 (0.93–1.11)	0.692	0%	0.90–1.13	0.91–1.14	0%	26.8%	3230
Low risk of bias (4 trials)	1.01 (0.92–1.12)	0.813	1.01 (0.91–1.12)	0.858	0%	0.67–1.52	0.67–1.52	0%	29.8%	2801
High risk of bias (20 trials)	1.00 (0.87–1.16)	0.994	1.04 (0.89–1.22)	0.643	0%	0.80–1.24	0.82–1.32	0%	21.1%	4380
Received PPI (9 trials)	1.02 (0.93–1.13)	0.647	1.02 (0.93–1.13)	0.619	0%	0.82–1.28	0.82–1.28	0%	28.5%	2976
Received H2RA (18 trials)	0.97 (0.83–1.14)	0.711	0.99 (0.83–1.18)	0.921	0%	0.74–1.28	0.73–1.35	0%	22.3%	4089
Received EN (12 trials)	1.05 (0.96–1.16)	0.264	1.05 (0.96–1.16)	0.254	0%	0.88–1.27	0.88–1.27	0%	28%	3048
Did not receive EN (12 trials)	0.85 (0.71–1.02)	0.077	0.82 (0.66–1.02)	0.075	0%	0.63–1.15	0.58–1.17	0%	21.7%	4230
*Pneumonia*										
All trials (12 trials)	1.09 (0.95–1.24)	0.221	1.07 (0.94–1.22)	0.321	0%	0.90–1.31	0.89–1.29	0%	14.8%	6681
Low risk of bias (3 trials)	1.00 (0.86–1.17)	0.959	1.00 (0.86–1.17)	0.954	0%	0.79–1.27	0.79–1.27	0%	16.3%	5972
High risk of bias (9 trials)	1.47 (1.10–1.97)	0.010	1.40 (1.03–1.89)	0.032	0%	0.44–4.87	0.40–4.83	0%	9.9%	10,496
Received PPI (6 trials)	1.03 (0.89–1.20)	0.668	1.04 (0.89–1.20)	0.642	0%	0.82–1.29	0.83–1.30	0%	15.2%	6478
Received H2RA (8 trials)	1.39 (1.01–1.91)	0.046	1.29 (0.93–1.80)	0.130	0%	0.37–5.13	0.33–5.03	0%	12.3%	8246
Received EN (8 trials)	1.09 (0.95–1.25)	0.236	1.07 (0.93–1.24)	0.312	0%	0.88–1.34	0.87–1.32	0%	15.1%	6528
Did not receive EN (4 trials)	1.08 (0.65–1.80)	0.757	1.02 (0.63–1.67)	0.935	0%	0.14–8.58	0.14–7.53	0%	11.8%	8640
*Clostridium difficile infection*										
All trials (4 trials)	0.78 (0.45–1.35)	0.377	0.78 (0.46–1.34)	0.370	0%	0.08–7.32	0.09–7.01	0%	1.6%	70,261
Low risk of bias (3 trials)	0.75 (0.43–1.31)	0.309	0.74 (0.43–1.29)	0.290	0%	0.08–7.26	0.08–7.01	0%	1.7%	66,068
High risk of bias (1 trial)	3.06 (0.13–74.19)	0.492	3.06 (0.1–74.19)	0.492		–	–	–	0%	–
Received PPI (4 trials)	0.78 (0.45–1.35)	0.377	0.78 (0.46–1.34)	0.370	0%	0.08–7.32	0.09–7.01	0%	1.6%	70,261
Received EN (4 trials)	0.78 (0.45–1.35)	0.377	0.78 (0.46–1.34)	0.370	0%	0.08–7.32	0.09–7.01	%	1.6%	70,261
Tertiary endpoints										
*Duration of ICU stay*										
All trials (11 trials)	−0.23 (−1.19–0.73)	0.635	−0.13 (−0.90–0.65)	0.751	18.4%					
Low risk of bias (3 trials)	0.11 (−3.20–3.43)	0.947	−0.42 (−2.02–1.18)	0.607	54.3%					
High risk of bias (8 trials)	−0.16 (−1.18–0.86)	0.757	−0.03 (− 0.92–0.85)	0.938	10.5%					
Received PPI (5 trials)	− 0.54 (− 1.66–0.59)	0.349	− 0.57 (− 1.63–0.49)	0.294	7.0%					
Received H2RA (7 trials)	−0.20 (−1.94–1.54)	0.820	0.38 (−0.76–1.52)	0.510	22.6%					
Received EN (7 trials)	−0.00 (−1.15–1.15)	0.998	0.05 (−0.79–0.88)	0.915	30.5%					
Did not receive EN (4 trials)	−1.18 (−3.26–0.90)	0.266	−1.18 (− 3.26–0.90)	0.266	0%					
*Duration of MV*										
All trials (7 trials)	−0.41 (−1.42–0.61)	0.434	−0.41 (−1.42–0.61)	0.434	0%					
Low risk of bias (3 trials)	−0.01 (−2.56–2.53)	0.991	−0.52 (−1.80–0.76)	0.423	46.6%					
High risk of bias (4 trials)	−0.21 (−1.87–1.46)	0.809	−0.21 (−1.87–1.46)	0.809	0%					
Received PPI (4 trials)	−0.04 (−1.63–1.55)	0.965	−0.35 (−1.55–0.85)	0.567	20.8%					
Received H2RA (4 trials)	−0.57 (−2.57–1.43)	0.574	−0.55 (− 2.47–1.37)	0.576	5.6%					
Received EN (6 trials)	−0.25 (−1.43–0.93)	0.676	−0.37(−1.40–0.66)	0.481	11.5%					
Did not receive EN (1 trial)	−2.00 (−8.83–4.83)	0.566	−2.00 (−8.83–4.83)	0.566	–					

*ICU* intensive care unit, *PPI* proton pump inhibitors, *H2RA* histamine2 receptor antagonists, *EN* enteral nutrition, *MV* mechanical ventilation, *GI* gastrointestinal TSA trial sequential analysis, *WMD* weighted mean difference, *RR* relative risk, *CI* confidence interval

“– “means unavailable data due to too little information used

TSA was conducted with an adjusted type I error of 3.3% for the primary endpoints and 2.5% for the secondary endpoints, power of 80%, D^2^ suggested by the included trials, relative risk reduction of 20%, two-tailed. If the actual measured D^2^ was zero, a D^2^ of 25% was used, because in this case heterogeneity would most likely increase when further studies are included

### Trial sequential analysis

Because of the increased risk of random errors resulted from sparsity data and repeated significance testing [23], we conducted trial sequential analysis (TSA) for the primary and secondary outcomes to assess this risk using TSA program version 0.9.5.10 beta (available from www.ctu.dk/tsa). We calculated the TSA-adjusted CI using fixed-effects and random-effects models for heterogeneity [D^2^ adjustment], and the random-effects result was reported as the main result (Table 1). Because the overall risk of falsely rejecting the null hypothesis (the type I error) will increase when performing multiple hypothesis tests [22], we calculated the TSA-adjusted 95% CI with a type I error of 5% with a statistical signifcance level of 3.3% for the co-primary outcomes and 2.5% for the co-secondary outcomes, a beta (power) of 80%, and a D^2^ suggested by the included trials. If the actual measured D^2^ was zero, a D^2^ of 25% was used because the heterogeneity may be expected to increase when further studies are included [22]. The required information size was calculated based on a relative risk reduction of 20% in the risk of clinically important GI bleeding and overt GI bleeding in the control group calculated from the conventional meta-analysis [21].

## Results

### Study selection

**Fig. 1:**
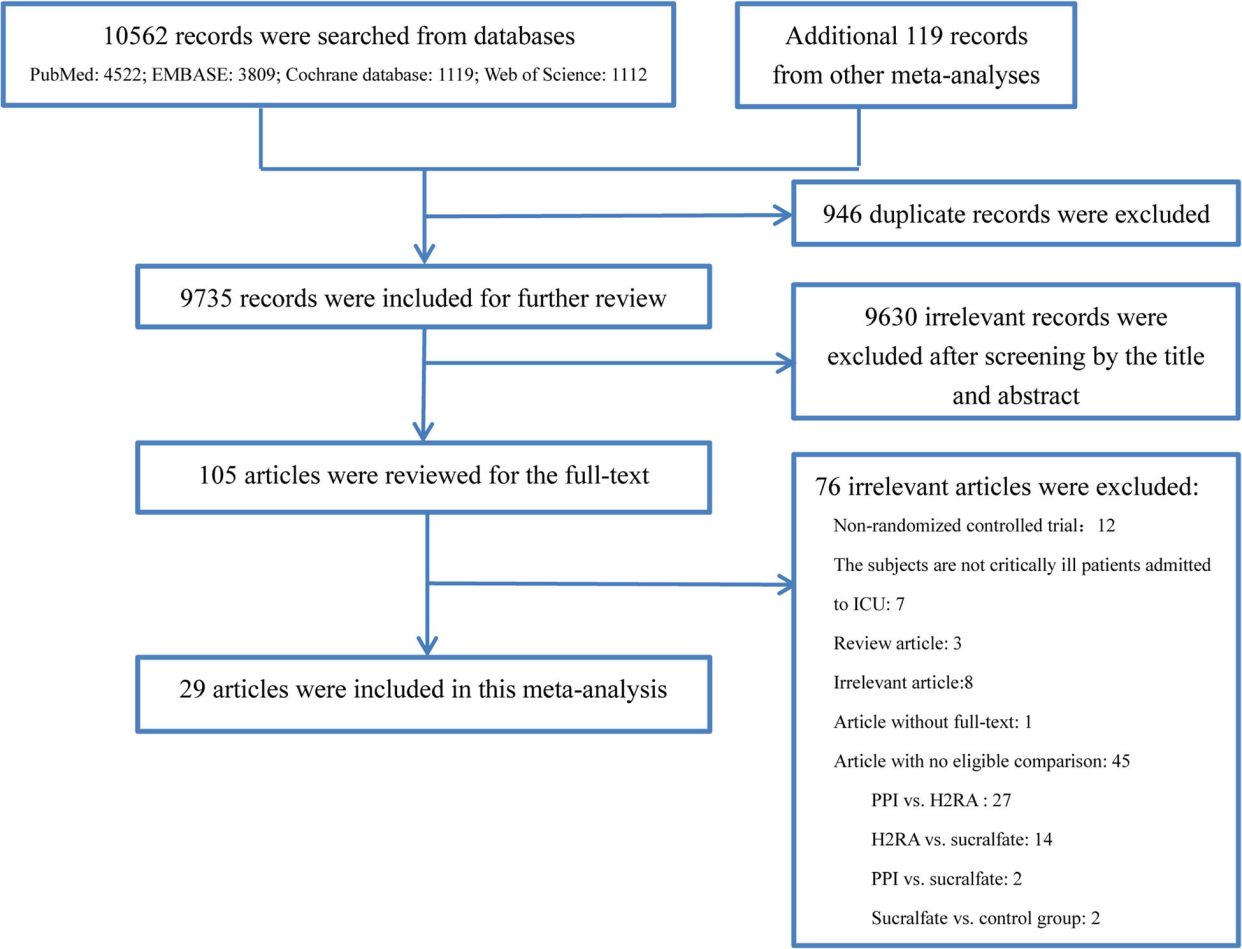
The PRISMA flowchart of the study selection process

The PRISMA flowchart of this study is shown in Fig. 1 and the reasons for exclusion of illegible studies are presented in (Additional file 1: Table S1). We searched a total of 10,562 records in the abovementioned database, additional 119 records from other meta-analyses were also identified. Among these records, 946 duplicate records and 9630 records, which were ineligible for our inclusion criteria, were excluded after screening the title and abstract. Then, the remaining 105 studies were included in the review of the full text. Finally, a total of 29 studies [7–13, 18, 24–44] met the eligibility criteria and were included in this meta-analysis.

### Characteristics of included studies

The detailed characteristics and clinical outcomes of the individual studies are described in (see Additional file 1: Table S2 and Additional file 1: Table S3). Among these included RCTs [7–13, 18, 24–44], six trials [18, 24, 30, 36, 38, 43] were published in recent 3 years, seven trials [11–13, 18, 24, 30, 44] were multi-center, and twenty-two trials [7–10, 25–29, 31–43] were single-center. The discrepancy in the number of subjects between trials were large and it varied from 25 to 3298. The subjects were from medical ICU [7, 25, 26, 30, 31, 38, 40], surgical ICU [8, 10, 13, 28, 34, 35, 37, 39, 41, 44], or both [9, 11, 12, 18, 24, 27, 29, 32, 33, 36, 42, 43]. SUP with H2RA was reported in 20 trials [7–13, 25–29, 31, 32, 34, 38, 40–42, 44], of which 9 trials [9, 13, 25, 28, 29, 34, 38, 41, 42] used ranitidine and 11 trials [7, 8, 10–12, 26, 27, 31, 32, 40, 44] used cimetidine. There are 6 trials in which the intervention group received PPI as SUP [8, 24, 30, 33, 36, 43], and it is worth mentioning that the trial by Gursoy et al. [33] set 5 intervention groups and one control group, all intervention groups received PPI, hence we combined the intervention groups into a single group to compare with the control group. Notably, the trials by Kantorova et al. [35] and Liu et al. [37] set 2 intervention groups in which one group used PPI and another group used H2RA, therefore the control group was divided evenly into two groups to make two comparisons. Additionally, as to the trial by Powell et al. [39] which have 3 intervention groups (one group received H2RA and two groups received PPI), we combined the two groups received PPI into a single group, and divided the control group into two groups. In all included trials, the dosage, duration of drug used and drug-delivery methods were largely different in the intervention group. The control groups received placebo [10–13, 18, 24, 27–35, 37, 39, 40, 43] or no prophylaxis [7–9, 25, 26, 36, 38, 41, 42, 44]. The subjects in 16 trials did not receive EN [7–9, 11–13, 28, 29, 31–34, 37, 39, 41, 44], however part or all patients received EN in the remaining 13 trials [10, 18, 24–27, 30, 35, 36, 38, 40, 42, 43].

### Risk of bias assessment

The full details of the risk of bias assessment of each included trials were presented in Table 2. There were 4 studies adjudicated as low risk of bias in all domains [18, 24, 30, 34], and all other studies were deemed overall high risk of bias due to at least one domain with an unclear or high risk of bias.

**Table 2:** Assessment of the risk of bias of each included trials

First author /Publication year	Adequate Sequence Generation	AllocationConcealment	Blinding of participants and personnel	Blinding of outcome assessment	Incomplete Outcome Data	Selective reporting	Other Bias	Overall risk of bias
Alhazzani/2017	L	L	L	L	L	L	L	L
Apte/1992	unclear	unclear	H	unclear	L	L	H	H
Basso/1981	L	unclear	H	L	H	H	unclear	H
Ben-Menachem/1994	L	L	H	L	H	L	L	H
van den Berg /1985	unclear	unclear	L	L	L	H	L	H
Burgess/1995	L	unclear	L	L	H	L	unclear	H
Calvet/1998	unclear	L	L	L	L	H	L	H
Darlong/2003	unclear	unclear	H	unclear	H	H	L	H
El-Kersh/2018	L	L	L	L	L	L	L	L
Friedman/1982	unclear	unclear	L	L	L	H	H	H
Groll/1986	unclear	unclear	L	L	L	L	H	H
Gursoy/2008	L	unclear	L	L	L	L	L	H
Halloran/1980	L	unclear	L	L	L	L	H	H
Hanisch/1998	L	L	L	L	L	L	L	L
Kantorova/2004	L	L	unclear	L	L	L	unclear	H
Karlstadt/1990	unclear	unclear	L	L	L	L	unclear	H
Krag/2018	L	L	L	L	L	L	L	L
Lin/2016	unclear	unclear	unclear	unclear	L	L	L	H
Liu/2013	L	unclear	unclear	unclear	L	L	unclear	H
Macdougall/1977	unclear	unclear	H	H	L	L	unclear	H
Martin/1993	unclear	unclear	L	L	L	L	H	H
Metz/1993	L	L	L	L	L	H	H	H
Nourian/2018	L	unclear	unclear	unclear	L	L	L	H
Peura/1985	unclear	unclear	L	L	L	L	L	H
Powell/1993	H	H	H	unclear	L	L	L	H
Reusser/1990	unclear	unclear	H	unclear	L	L	L	H
Ruiz-Santana/1991	unclear	unclear	H	H	H	L	L	H
Selvanderan/2016	L	unclear	L	L	L	L	L	H
Zinner/1981	L	H	H	unclear	H	unclear	L	H

*L* low, *H* high

### Analysis of the primary outcomes

#### The clinically important GI bleeding

**Fig. 2:**
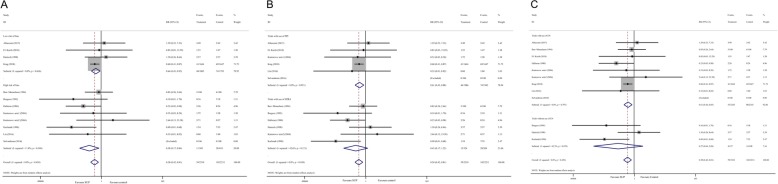
Forest plot of subgroup analysis for the clinically important GI bleeding based on the trials quality (panel **a**), the type of SUP used (panel **b**), and whether EN was used (panel **c**). SUP stress ulcer prophylaxis; GI gastrointestinal; RR relative risk; PPI proton pump inhibitors; H2RA histamine 2 receptor antagonists; EN enteral nutrition

**Fig. 3:**
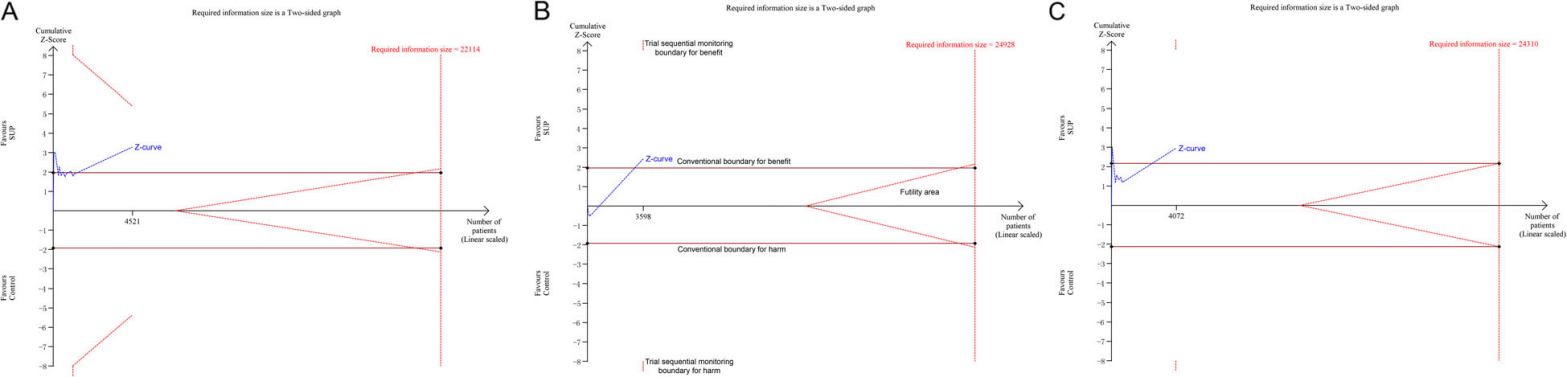
Trial sequential analysis for the clinically important GI bleeding. Trial sequential analysis using random-effects model with a relative risk reduction of 20%, an adjusted type I error of 3.3%, power of 80%. (panel **a**) In all trials reported data on the clinically important GI bleeding, control event proportion of 4.6%, D2 of 25% (the actual measured D2 was 0%), the cumulative Z-curve cross no boundaries, the required information size of 22,114 patients are not reached. The TSA-adjusted 95% CI for an RR of 0.58 is 0.23 to1.51. (panel **b**) In trials with low risk of bias, control event proportion of 4.1%, D2 of 25% (the actual measured D2 was 0%), the cumulative Z-curve cross no boundaries, the required information size of 24,928 patients are not reached. The TSA-adjusted 95% CI for an RR of 0.64 is 0.15 to 2.80. (panel **c**) In trials with use of EN, control event proportion of 4.2%, D2 of 25% (the actual measured D2 was 0%), the cumulative Z-curve cross no boundaries, the required information size of 24,310 patients are not reached. The TSA-adjusted 95% CI for an RR of 0.61 is 0.16 to 2.38. SUP stress ulcer prophylaxis; GI gastrointestinal; TSA trial sequential analysis; EN enteral nutrition

There were 11 studies [10, 11, 18, 24, 26, 28, 30, 34–36, 43], consisting of 4521 participants, reported data on the clinically important GI bleeding. The pooled results demonstrated that use of SUP was associated with a decreased risk of the clinically important GI bleeding (RR = 0.58, 95% CI: 0.42–0.81, *P* = 0.001,I^2^ = 0%), this benefit was also found in sub-analysis of trials with low risk of bias (RR = 0.64, 95% CI: 0.45–0.92, *P* = 0.017;I^2^ = 0%) (Fig. 2.A), trials used PPI (RR = 0.61, 95% CI: 0.43–0.88, *P* = 0.008; I^2^ = 0%) (Fig. 2.B), and trials received EN (RR = 0.61, 95% CI: 0.44–0.85, *P* = 0.004; I^2^ = 0%) (Fig. 2.C), but not in trials used H2RA and trials did not receive EN. However, the results from TSA showed that the Z-curve just crossed the conventional boundary for benefit, but not the trial sequential monitoring boundary for benefit (Fig. 3, and in Additional file 1: Figure S1), indicated that it still lack of sufficient evidence to favor a 20% relative risk reduction in the risk of clinically important GI bleeding with use of SUP.

#### The overt GI bleeding

**Fig. 4:**
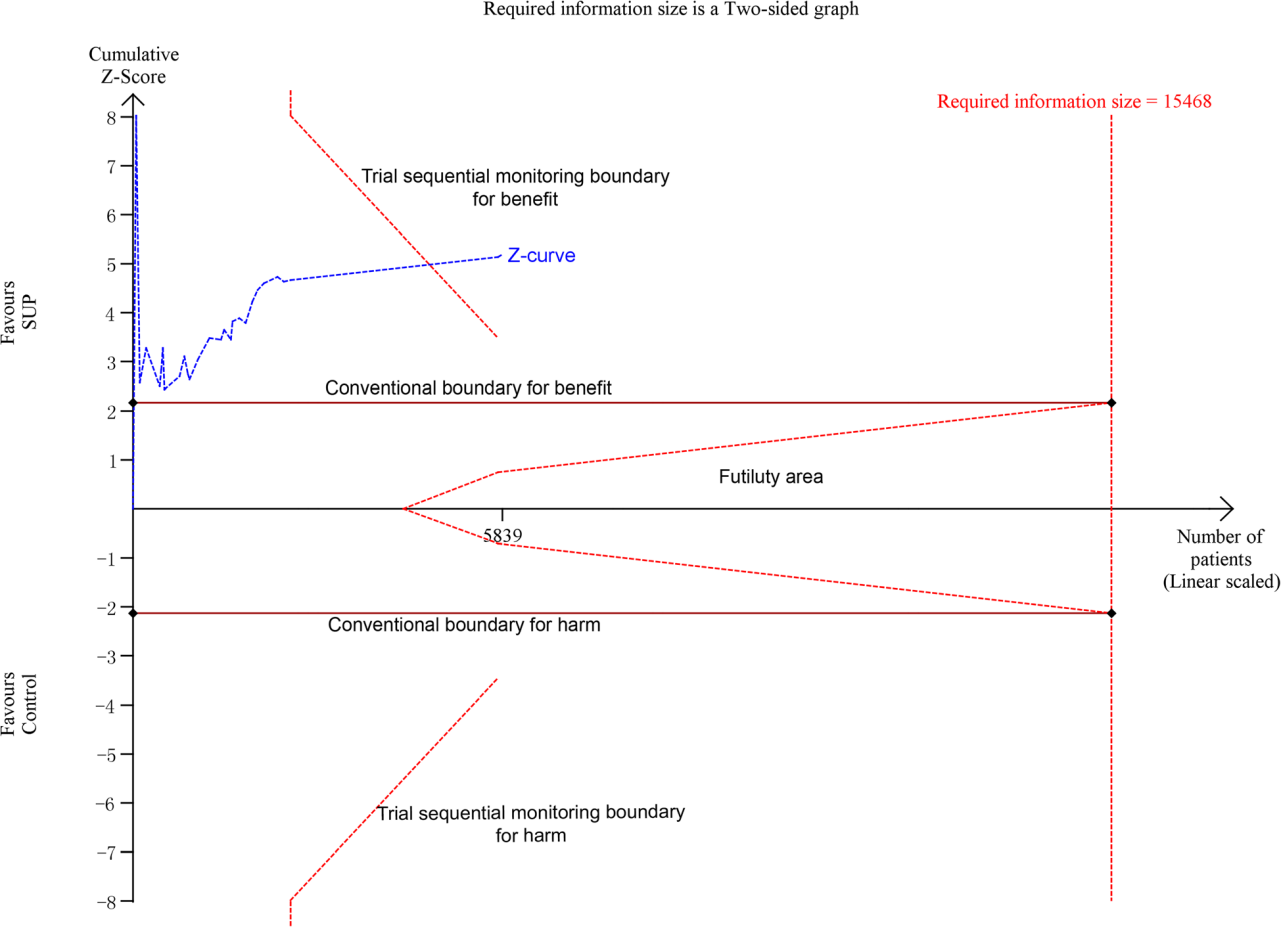
Trial sequential analysis for the overt GI bleeding in all included trials. Trial sequential analysis using random-effects model with an adjusted type I error rate of 3.3%, power of 80%, D2 of 62%, for an relative risk reduction of 20% in control event proportion of 12.1%. The Z-curve cross the trial sequential monitoring boundary for benefit, but do not reach the required information size of 15,468 participants. The TSA-adjusted 95% CI for an RR of 0.48 is 0.31 to 0.75. SUP stress ulcer prophylaxis; GI gastrointestinal; TSA trial sequential analysis

**Fig. 5:**
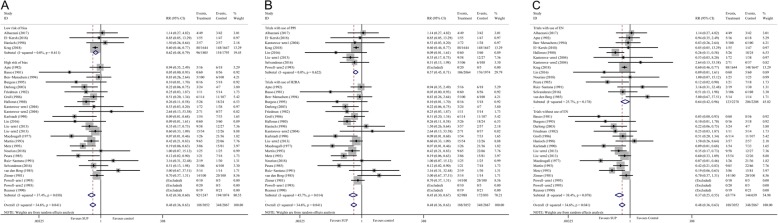
Forest plot of subgroup analysis for the overt GI bleeding based on the trials quality (panel **a**), the type of SUP used (panel **b**), and whether EN was used (panel **c**). SUP stress ulcer prophylaxis; GI gastrointestinal; RR relative risk; PPI proton pump inhibitors; H2RA histamine 2 receptor antagonists; EN enteral nutrition

**Fig. 6:**
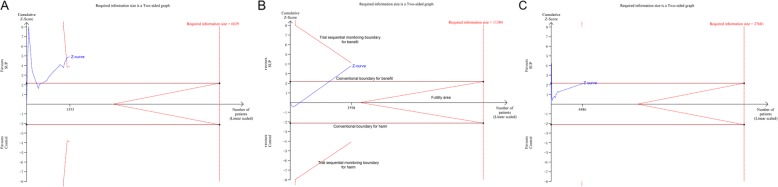
Trial sequential analysis for the overt GI bleeding. Trial sequential analysis using random-effects model with a relative risk reduction of 20%, an adjusted type I error of 3.3%, power of 80%. (panel **a**) In trials did not receive EN, control event proportion of 21.9%, D2 of 52%, the cumulative Z-curve cross cross the trial sequential monitoring boundary for benefit, the required information size of 6029 patients are not reached. The TSA-adjusted 95% CI for an RR of 0.37 is 0.22 to 0.63. (panel **b**) In trials with low risk of bias, control event proportion of 8.6%, D2 of 25% (the actual measured D2 was 0%), the cumulative Z-curve cross no boundaries, the required information size of 11,384 patients are not reached. The TSA-adjusted 95% CI for an RR of 0.62 is 0.37 to 1.04. (panel **c**) In trials with use of EN, control event proportion of 9.2%, D2 of 71%, the cumulative Z-curve cross no boundaries, the required information size of 27,681 patients are not reached. The TSA-adjusted 95% CI for an RR of 0.64 is 0.12 to 3.35. SUP stress ulcer prophylaxis; GI gastrointestinal; TSA trial sequential analysis; EN enteral nutrition

There were 27 studies [7–13, 18, 24–28, 30–32, 34–44], with 30 comparisons, including 5919 participants, reported available data regarding overt GI bleeding. The pooled RR from the conventional meta-analysis for overt GI bleeding in all trials was 0.48 (95% CI: 0.36–0.63, *P* < 0.001; I^2^ = 34.6%), the TSA showed that the trial sequential monitoring boundary for benefit was crossed with TSA-adjusted CI from 0.31 to 0.75 (D^2^ = 62%) (Fig. 4), indicating a firm evidence on the beneficial effect of SUP on overt GI bleeding. In the sub-analyses of trials based on the trials quality (Fig. 5.A), the type of SUP used (Fig. 5.B), and whether EN was used (Fig. 5.C), we also found a significant reduction in the incident of overt GI bleeding with use of SUP. Furthermore, TSA showed that the Z-curve crossed the trial sequential monitoring boundary for benefit in trials did not receive EN (Fig. 6.A), in trials used PPI as SUP (Additional file 1: Figure S2.A), and in trials used H2RA as SUP (Additional file 1: Figure S2.B). However, the TSA for trials adjudicated as low risk of bias (Fig. 6.B) and trials received EN (Fig. 6.C) revealed a neutral benefit in reducing the risk of overt GI bleeding, thus more evidence is required to confirm this benefits in this case.

#### Analysis of the secondary outcomes

There are 24 RCTs [7, 10–12, 18, 24–26, 28–30, 32–44] included in analysis for all-cause mortality, the conventional meta-analysis indicated that SUP was not associated with a lower mortality compared with the control group, regardless of the trials quality (Additional file 1: Figure S3.A), the type of SUP used (Additional file 1: Figure S3.B), and whether EN was used (Additional file 1: Figure S3.C). These results was firmly confirmed by TSA in which the Z-curve reached the futility area, indicating that there are sufficient events in current trials to reject a 20% relative risk reduction in mortality with use of SUP (Additional file 1: Figure S4). However, it still lack of sufficient evidence on the effects of SUP on mortality in patients did not receive EN (Additional file 1: Figure S4.F), because the Z-curve did not cross any boundaries.

In the meta-analysis of 12 trials [11, 12, 18, 24–26, 34–38, 43] refered to data on pneumonia, we found that administraion of SUP was not accompanied by increased risk of pneumonia, TSA further confirmed this result with the Z-curve crossing the boundary for futility (Additional file 1: Figure S5.A). Also, negtive results were observed in sub-analysis of trials with low risk of bias (Additional file 1: Figure S6.A), in trials used PPI as SUP (Addtional file 1: Figure S6.B), in trials used H2RA as SUP (Additional file 1: Figure S6.B), in trials with use of EN (Additional file 1: Figure S6.C), or in trials without use of EN (Additional file 1: Figure S6.C), TSA suggested that the pooled results were robust in subgroup of trials with low risk of bias (Additional file 1: Figure S5.B), trials received PPI (Additional file 1: Fig. S5.C), and trials with use of EN (Additional file 1: Figure S5.E).

There are 4 trials presented data on CDI [18, 24, 30, 43], all of them received PPI and EN, three of them was judged have low risk of bias. There was no difference in the incident of CDI between SUP group and control group. Sub-analysis of trials with low risk of bias also revealed a neutral effect of SUP on the incident of CDI (Additional file 1: Figure S7). However, TSA demonstrated that the Z-curve crossed no boundaries (Additional file 1: Figure S8), indicating that this results could be changed when more studies were incuded.

#### Analysis of the tertiary outcomes

There are 11 studies reported data on the duration of ICU stay [12, 24, 26, 30, 33–35, 38, 41–43] and 7 studies [24, 26, 30, 34, 35, 38, 43] reported data on the duration of MV, the pooled results suggested that use of SUP could not result in a significant reduction in the duration of ICU stay (Additional file 1: Figure S9) and duration of MV (Additional file 1: Figure S10) when compared with placebo or no prophylaxis, regardless of the trials quality, the type of SUP used, and whether EN was used.

#### Analysis for publication bias

**Fig. 7:**
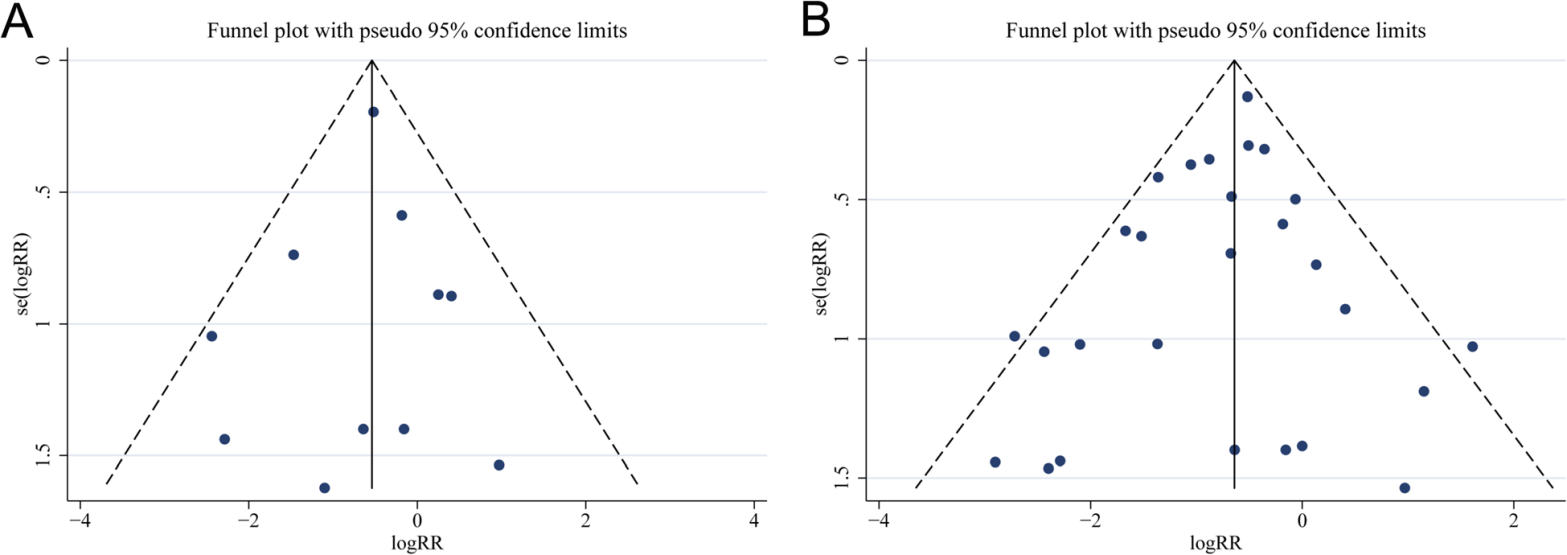
Funnel plots for evaluating the publication bias of included trials for theclinically important GI bleeding (Panel **a**) and overt GI bleeding (Panel **b**). Both funnel plots are visually symmetric, and the Begg’s and Egger’s tests reveals no significant publication bias. RR relative risk; GI GI gastrointestinal

We assessed the publication bias for the primary outcomes. All the funnel plots were visually symmetrical (Fig. 7). The Begg’s and Egger’s tests for the clinically important GI bleeding (*P* = 0.436 and *P* = 0.395, respectively) and for overt GI bleeding (*P* = 0.617 and *P* = 0.280, respectively) revealed no evidence of publication bias.

## Discussion

In this meta-analysis, we found that use of SUP, with PPI or H2RA, was associated with a reduced incident of clinically important GI bleeding and overt GI bleeding, but had no effects on the all-cause mortality, incident of pneumonia and CDI, and duration of MV and ICU stay. After adjustment with TSA, we could firmly conclude that SUP had beneficial effects in reducing the incident of overt GI bleeding, this benefit was confirmed in patients who did not receive EN, but not in those who received EN, and SUP had also no effects on the mortality and pneumonia. However, it still needs more evidence to confirm the effects of SUP on others clinical outcomes.

Critically ill patients in the ICU always suffer from a series of change in hemodynamics, such as hypoxia, hypotension, stress, etc., these conditions can cause a reduction in GI mucosal blood flow and lead to a collapse of defense mechanisms of GI mucosa [45, 46], thus resulting in stress-related ulcer and subsequent GI bleeding. As to critically ill patients who are at high risk of GI bleeding, pharmacy prophylaxis is the first-line therapy for preventing stress-related ulcer, PPI and H2RA are the most common drugs for SUP. Over the past decades, nearly 30 RCTs were successively conducted to investigate the efficacy of SUP with PPI or H2RA in ICU patients, and the results from the most recent meta-analyses [15, 16] favor the use of SUP with PPI or H2RA in critically ill patients, however, the quality of evidence is low and the number of subjects is insufficient. Accordingly, the Surviving Sepsis Campaign guidelines [47] for septic shock just weakly recommended use of PPI or H2RA as SUP for patients with sepsis or septic shock who have risk factors for GI bleeding. Moreover, the trial by Apte et al. [25] revealed a higher incidence of pneumonia in patients received H2RA, some observational studies [6, 48, 49] also found an increased risk of infectious complications, including pneumonia and CDI, in critically ill patients receiving SUP, thus concerns on the balance between benefits and harms of SUP raised in recent years.

In this meta-analysis, we found that administration of PPI or H2RA has benefits in reducing the risk of overt GI bleeding, and might reduce the risk of clinically important GI bleeding, but has no benefits for all-cause mortality, these results were similar with the study by Reynolds et al. [15] and by Karg et al. [50]. This finding caused our question that the benefit of SUP in preventing GI bleeding cannot be translated into beneficial effect on mortality. The potential explation includes that overt GI bleeding indicates slight haemorrhage in GI tract without influence on hemodynamics and did not need transfusion or surgical intervention, thus the reduction in overt GI bleeding might not produce a benefit in mortality. Additionally, *Clostridium difficile*–associated diarrhea has become an increasing challenge in ICU, a massive fluid loss would lead to changes of hemodynamics and disorder of internal environment, and thus, result in a high mortality and prolonged ICU stay [6, 50]. Furthermore, gastric acid is a natural barrier against pathogens, suppressing secretion of gastric acid by PPI or H2RA will break this barrier and results in a bacterial overgrowth and colonization, which will cause infectious compilcations [51]. Therefore, the above adverse effects might counterbalance the benefit in mortality resulted from the decreased risk of clinically important GI bleeding with use of SUP. In addition, we also found a beneficial effect of SUP on overt GI bleeding among patients who did not receive EN, however, this benefit were not sure in patients who received EN, the study by Huang et al. [17] also found that SUP is not beneficial for ICU patients received EN to reduce the GI bleeding. Nowadays, EN has been recongnized as the first choice of nutrition routine for critically ill patients, EN is expected to prevent GI bleeding by increasing GI mucosa blood flow and limiting the bacterial translocation through maintenance of gut integrity [52–55]. Previous studies has demonstrated that EN might provide sufficient prophylaxis against stress-related GI bleeding [56, 57], there are even opinions that EN might be more effective than SUP in preventing GI bleeding [58]. Therefore, SUP maybe more effective in reducing overt GI bleeding in patients who did not receive EN than those who received EN.

Two strengths in this meta-analysis should be mentioned. Firstly, our meta-analysis has a larger sample size. Different from the previous meta-analysis [14–17], our study included the SUP-ICU trial [18], in which the number of the subject is more than 3000, which are larger than the total number of subjects in all previous trials. Therefore, our meta-analysis with a larger sample size would decrease the sampling errors and selective bias to some extent and reveal the outcome effects more objectively [21]. In addition, TSA suggests that sufficient events had been accrued in the current trials to draw a firm conclusion on the effect of SUP on the risk of overt GI bleeding, this benefit was more obvious in patients did not receive EN, however, convinced evidence regarding the efficacy of SUP in patients received EN are still lacking, hence this results could be referenced to guide future trial to focus on the use of SUP in critically ill patients received EN. Secondly, a more reasonable method was implemented in our meta-analysis to handle the data in trials with multiple intervention groups. As to trials with multiple intervention groups, the optimal approach recommended by the Cochrane Collaboration [20] is to combine all relevant intervention groups into a single group. However, in the meta-analyses by Reynolds et al. [15] and by Krag et al. [59], they compared the multiple intervention groups with the “shared” control group and then simply entered multiple comparisons into meta-analysis, this approach should be avoided on account of a unit-of-analysis error, which was associated with the unaddressed correlation between the estimated intervention effects from multiple comparisons [20]. To overcome this error, we combined all intervention groups into a single group to compare with the “shared” control group if the intervention groups received the same type of SUP, or split the “shared” control group into multiple groups to make multiple pair-wise comparisons if the intervention groups received a different type of SUP. We also adjusted the thresholds for significance based on the number of outcomes because of problems with multiplicity, this is another methodological strength in our study.

However, there are several limitations in our study. First, subgroup analysis was conducted based on the type of SUP, the trials quality, and status of EN, not for other variables. We noticed that there was significant statistical heterogeneity in the analysis for the overt GI bleeding, and the clinical heterogeneity between studies is substantial, the dosage, route of administration, infusion duration of drug used, and ICU setting are varied between the included trials, unfortunately, we did not perform a sub-analysis based on these factors. Additionally, there is a big difference in the number of subjects between included trials, the possible overestimation of effect size in studies with a small sample size should be considered when interpreting the results. Second, TSA in our study was performed for the primary and secondary outcomes, but not for the tertiary outcomes, cumulative meta-analysis of sparse data and repeated significance testing always lead to increased risk of random error, TSA could prevent this risk [23], hence the results of conventional meta-analyses for the tertiary outcomes should be interpreted carefully. Lastly, there was a considerable variation in the definition of overt GI bleeding, clinically important GI bleeding and pneumonia in some studies, this variation might be translated into statistical heterogeneity.

## Conclusions

This meta-analysis with TSA firmly conclude that there are sufficient evidence in favor of the benefit of SUP in reducing the risk of overt GI bleeding in critically ill patients, those who did not receive EN might suffer from this benefit more obviously, future trials are unlikely to detect a 20% reduction in all-cause mortality and pneumonia, however, the effect of SUP on clinically important GI bleeding still needs more evidence to confirm.

## Supplementary information

**Additional file 1:** the supplementary materials regarding search strategy, reasons for exclusion of ineligible studies, detailed information on included studies, and supplementary tables and figures for analyzing the clinical outcomes.

## Data Availability

All data generated or analyzed during this study are included in this published article (and its supplementary information files).

## Abbreviations

CDI: *Clostridium difficile* infection
CI: Confidence interval
EN: Enteral nutrition
GI: Gastrointestinal
H2RA: Histamine2 receptor antagonists
ICU: Intensive care unit
IQR: Interquartile rang
MV: Mechanical ventilation
PPI: Proton pump inhibitors;
RCTs: Randomized controlled trials
RR: Relative risk
SD: Standard deviation
SUP: Stress ulcer prophylaxis
TSA: Trial sequential analysis
WMD: Weighted mean difference
