# Supplementary material for: Stress ulcer prophylaxis with proton pump inhibitors or histamine 2 receptor antagonists in critically ill adults - a meta-analysis of randomized controlled trials with trial sequential analysis
Source: BMC Gastroenterol. 2019 Nov 21;19:193. doi: 10.1186/s12876-019-1105-y (PMC6873751; doi:10.1186/s12876-019-1105-y)
Supplement: Supplementary file 1 — Additional file 1: the supplementary materials regarding search strategy, reasons for exclusion of ineligible studies, detailed information on included studies, and supplementary tables and figures for analyzing the clinical outcomes. [file 12876_2019_1105_MOESM1_ESM.docx]

**SUPPLEMENTARY MATERIALS**

## Table of contents

[Table of contents 1](#_Toc12201708)

[The search strategy 2](#_Toc12201709)

[Table S1. The reasons for exclusion of ineligible studies 5](#_Toc12201710)

[Table S2. The detailed characteristics of all included trials 11](#_Toc12201711)

[Table S3. The detailed data on clinical outcomes in all included trials 16](#_Toc12201712)

[Figure S1. Trial sequential analysis for the clinically important GI bleeding in trials with use of PPI 18](#_Toc12201713)

[Figure S2. Trial sequential analysis for the overt GI bleeding in trials with use of PPI and trials with use of H2RA 19](#_Toc12201714)

[Figure S3. The conventional meta-analysis for the all-cause mortality 21](#_Toc12201715)

[Figure S4. Trial sequential analysis for the all-cause mortality 24](#_Toc12201716)

[Figure S5. Trial sequential analysis for the incident of pneumonia 28](#_Toc12201717)

[Figure S6. The conventional meta-analysis for the incident of pneumonia 32](#_Toc12201718)

[Figure S7. Sub-analysis of trials based on the trials quality for the incident of clostridium difficile infection 34](#_Toc12201719)

[Figure S8. Trial sequential analysis for the incident of clostridium difficile infection 35](#_Toc12201720)

[Figure S9. The conventional meta-analysis for the duration of ICU stay 37](#_Toc12201721)

[Figure S10. The conventional meta-analysis for the duration of mechanical ventilation 39](#_Toc12201722)

## The search strategy

1. **Pubmed (4522 records)：**

#1. ((((((((Proton Pump Inhibitors[MeSH Terms]) OR Proton Pumps[MeSH Terms]) OR Omeprazole[MeSH Terms]) OR Esomeprazole[MeSH Terms]) OR Benzimidazoles[MeSH Terms]) OR 2-Pyridinylmethylsulfinylbenzimidazoles[MeSH Terms]) OR Lansoprazole[MeSH Terms]) OR Dexlansoprazole[MeSH Terms]) OR Rabeprazole[MeSH Terms]

#2. ((((((((Histamine H2 Antagonists[MeSH Terms]) OR Cimetidine[MeSH Terms]) OR Guanidines[MeSH Terms]) OR Imidazoles[MeSH Terms]) OR Famotidine[MeSH Terms]) OR Thiazoles[MeSH Terms]) OR Nizatidine[MeSH Terms]) OR Ranitidine[MeSH Terms]) OR Furans[MeSH Terms]

#3. (((((((((((((((((((((((((((((((((((((((((((((((Proton Pump Inhibitor*) OR Proton Pump*) OR PPI) OR Benzimidazole*) OR Omeprazole*) OR losec) OR omez) OR prilosec) OR rapinex) OR zegerid) OR esomeprazole*) OR axagon) OR esporal) OR lucen) OR nexiam) OR nexium) OR sompraz) OR soleri) OR Lansoprazole*) OR agopton) OR bamalite) OR lanzor) OR monolitum) OR ogast) OR ogastro) OR ogastro) OR opiren) OR prevacid) OR prezal) OR proulco) OR promeco) OR takepron) OR ulpax) OR zoton) OR 2-Pyridinylmethylsulfinylbenzimidazole*) OR dexlansoprazole) OR kapidex) OR dexilant) OR pantoprazole) OR pantoloc) OR pantecta) OR protium) OR protonix) OR rabeprazole) OR AcipHex) OR Pariet) OR dexrabeprazole) OR rablet[all]

#4. (((((((((((((((((((((((((((((((((((((((((((((((((((Histamine H2 Antagonist*) OR histamine blocker*) OR H2 blocker*) OR histamine receptor*) OR histamine antagonist*) OR H2 antihistaminic*) OR H2RA*) OR Cimetidine*) OR Guanidine*) OR Imidazole*) OR acitak) OR altramet) OR biomet) OR dyspamet) OR eureceptor) OR galenamet) OR histodil) OR peptimax) OR phimetin) OR tagamet) OR ultec) OR zita) OR FAMOTIDINE) OR famotidine) OR fluxid) OR leader acid reducer) OR mylanta) OR pepcid) OR Thiazoles) OR roxatidine) OR Roxit) OR NIZATIDINE) OR Nizatidine) OR axid) OR tazac) OR zinga) OR RANITIDINE) OR ranitidine) OR azanplus) OR biotidin) OR pylorid) OR raciran) OR raniberl) OR ranisen) OR ranitidin) OR rantec) OR sostril) OR taladine) OR tritec) OR wal-zan) OR zantac) OR Furans[all]

#5. #1 OR #2 OR #3 OR #4

#6. (((Critical Care[MeSH Terms]) OR intensive care[MeSH Terms]) OR Critical Illness[MeSH Terms]) OR Intensive Care Units[MeSH Terms]

#7. (((((Critical Care) OR intensive care) OR Critical* illness) OR Intensive Care Unit*) OR ICU) OR intensive illness[all]

#8. #6 OR #7

#9. #5 AND #8

1. **Web of Science (1112 records)：**

#1. TS=(Proton Pump Inhibitor*) OR TS=(Proton Pump*) OR TS=(PPI) OR TS=(Benzimidazole*) OR TS=(Omeprazole*) OR TS=(losec) OR TS=(omez) OR TS=(prilosec) OR TS=(rapinex) OR TS=(zegerid) OR TS=(esomeprazole*) OR TS=(axagon) OR TS=(esporal) OR TS=(lucen) OR TS=(nexiam) OR TS=(nexium) OR TS=(sompraz) OR TS=(soleri) OR TS=(Lansoprazole*) OR TS=(agopton) OR TS=(bamalite) OR TS=(lanzor) OR TS=(monolitum) OR TS=(ogast) OR TS=(ogastro) OR TS=(ogastro) OR TS=(opiren) OR TS=(prevacid) OR TS=(prezal) OR TS=(proulco) OR TS=(promeco) OR TS=(takepron) OR TS=(ulpax) OR TS=(zoton) OR TS=(2-Pyridinylmethylsulfinylbenzimidazole*) OR TS=(dexlansoprazole) OR TS=(kapidex) OR TS=(dexilant) OR TS=(pantoprazole) OR TS=(pantoloc) OR TS=(pantecta) OR TS=(protium) OR TS=(protonix) OR TS=(rabeprazole) OR TS=(AcipHex) OR TS=(Pariet) OR TS=(dexrabeprazole) OR TS=(rablet)

#2. TS=(Histamine H2 Antagonist*) OR TS=(histamine blocker*) OR TS=(H2 blocker*) OR TS=(histamine receptor*) OR TS=(histamine antagonist*) OR TS=(H2 antihistaminic*) OR TS=(H2RA*) OR TS=(Cimetidine*) OR TS=(Guanidine*) OR TS=(Imidazole*) OR TS=(acitak) OR TS=(altramet) OR TS=(biomet) OR TS=(dyspamet) OR TS=(eureceptor) OR TS=(galenamet) OR TS=(histodil) OR TS=(peptimax) OR TS=(phimetin) OR TS=(tagamet) OR TS=(ultec) OR TS=(zita) OR TS=(FAMOTIDINE) OR TS=(famotidine) OR TS=(fluxid) OR TS=(leader acid reducer) OR TS=(mylanta) OR TS=(pepcid) OR TS=(Thiazoles) OR TS=(roxatidine) OR TS=(Roxit) OR TS=(NIZATIDINE) OR TS=(Nizatidine) OR TS=(axid) OR TS=(tazac) OR TS=(zinga) OR TS=(RANITIDINE) OR TS=(ranitidine) OR TS=(azanplus) OR TS=(biotidin) OR TS=(pylorid) OR TS=(raciran) OR TS=(raniberl) OR TS=( ranisen) OR TS=(ranitidin) OR TS=(rantec) OR TS=(sostril) OR TS=(taladine) OR TS=(tritec) OR TS=(wal-zan) OR TS=(zantac) OR TS=(Furans)

#3. #1 OR #2

#4. TS=(Critical Care) OR TS=(intensive care) OR TS=(Critical* illness) OR TS=(Intensive Care Unit*) OR TS=(ICU) OR TS=(intensive illness) OR TS=(Critical* ill*)

#5. #3 AND #4

1. **EMBASE (3809 records)：**

#1.(proton AND pump AND inhibitor*) OR (proton AND pump*) OR ppi OR benzimidazole* OR omeprazole* OR 'losec'/exp OR losec OR omez OR tprilosec OR 'rapinex'/exp OR rapinex OR'zegerid'/exp OR zegerid OR esomeprazole* OR axagon OR tesporal OR lucen OR 'nexiam'/exp OR nexiam OR tnexium OR 'sompraz'/exp OR sompraz OR soleri OR lansoprazole* OR'agopton'/exp OR agopton OR 'bamalite'/exp OR bamalite OR 'lanzor'/exp OR lanzor OR 'monolitum'/exp OR monolitum OR 'ogast'/exp OR ogast OR 'ogastro'/exp OR ogastro OR'opiren'/exp OR opiren OR 'prevacid'/exp OR prevacid OR 'prezal'/exp OR prezal OR proulco OR promeco OR 'takepron'/exp OR takepron OR 'ulpax'/exp OR ulpax OR 'zoton'/exp ORzoton OR '2 pyridinylmethylsulfinylbenzimidazole*' OR 'dexlansoprazole'/exp OR dexlansoprazole OR 'kapidex'/exp OR kapidex OR 'dexilant'/exp OR dexilant OR 'pantoprazole'/exp OR pantoprazole OR 'pantoloc'/exp OR pantoloc OR 'pantecta'/exp OR pantecta OR 'protium'/exp OR protium OR 'protonix'/exp OR protonix OR 'rabeprazole'/exp OR rabeprazole OR'aciphex'/exp OR aciphex OR 'pariet'/exp OR pariet OR 'dexrabeprazole'/exp OR dexrabeprazole OR rable

#2.(histamine AND h2 AND antagonist*) OR (histamine AND blocker*) OR (h2 AND blocker*) OR (histamine AND receptor*) OR (histamine AND antagonist) OR (h2 AND antihistaminic*) ORh2ra* OR cimetidine* OR guanidine* OR imidazole* OR acitak OR altramet OR biomet OR dyspamet OR eureceptor OR galenamet OR histodil OR peptimax OR phimetin OR tagamet ORultec OR zita OR famotidine OR fluxid OR (leader AND acid AND reducer) OR mylanta OR pepcid OR thiazoles OR roxatidine OR roxit OR nizatidine OR axid OR tazac OR zinga ORranitidine OR azanplus OR biotidin OR pylorid OR raciran OR raniberl OR ranisen OR ranitidin OR rantec OR sostril OR taladine OR tritec OR 'wal zan' OR zantac OR furans

#3. #1 OR #2

#4.(critical AND care) OR (intensive AND care) OR (critical* AND illness) OR (intensive AND care AND unit*) OR icu OR (intensive AND illness) OR (critical* AND ill)

#5. #3 AND #4

#6. #5 AND ('human'/de OR 'randomized controlled trial'/de) AND 'article'/it

1. [**Cochrane Library**](http://www.xttsg.com/e/public/jump/?classid=155&id=209) **(1119 records)：**

#1.MeSH descriptor: [Proton Pump Inhibitors] explode all trees

#2.MeSH descriptor: [Proton Pumps] explode all trees

#3.MeSH descriptor: [Omeprazole] explode all trees

#4.MeSH descriptor: [Esomeprazole] explode all trees

#5.MeSH descriptor: [Benzimidazoles] explode all trees

#6.MeSH descriptor: [2-Pyridinylmethylsulfinylbenzimidazoles] explode all trees

#7.MeSH descriptor: [Lansoprazole] explode all trees

#8.MeSH descriptor: [Rabeprazole] explode all trees

#9.MeSH descriptor: [Dexlansoprazole] explode all trees

#10. #1 OR #32 OR #3 OR #4 OR #5 OR #6 OR #7 OR #8 OR #9

#11.MeSH descriptor: [Histamine H2 Antagonists] explode all trees

#12.MeSH descriptor: [Cimetidine] explode all trees

#13.MeSH descriptor: [Guanidines] in all MeSH products

#14.MeSH descriptor: [Imidazoles] explode all trees

#15.MeSH descriptor: [Famotidine] explode all trees

#16.MeSH descriptor: [Thiazoles] explode all trees

#17.MeSH descriptor: [Nizatidine] explode all trees

#18.MeSH descriptor: [Ranitidine] explode all trees

#19.MeSH descriptor: [Furans] explode all trees

#20.#11 OR #12 OR #13 OR #14 OR #15 OR #16 OR #17 OR #18 OR #9

#21.MeSH descriptor: [Critical Care] explode all trees

#22.MeSH descriptor: [Critical Illness] explode all trees

#23.MeSH descriptor: [Intensive Care Units] explode all trees

#24.#21 OR #22 OR #23

#25.(Proton Pump Inhibitor*):ti,ab,kw OR (Proton Pump*):ti,ab,kw OR (Omeprazole):ti,ab,kw OR (Esomeprazole):ti,ab,kw OR (Benzimidazole*):ti,ab,kw

#26.(*Pyridinylmethylsulfinylbenzimidazole*):ti,ab,kw OR (Lansoprazole):ti,ab,kw OR (Dexlansoprazole):ti,ab,kw OR (Pantoprazole):ti,ab,kw OR (Rabeprazole):ti,ab,kw

#27.(Histamine H2 Antagonist*):ti,ab,kw OR (H2RA):ti,ab,kw OR (Guanidine*):ti,ab,kw OR (Cimetidine):ti,ab,kw OR (Imidazole*):ti,ab,kw

#28.(Famotidine):ti,ab,kw OR (Thiazole*):ti,ab,kw OR (Nizatidine):ti,ab,kw OR (Ranitidine):ti,ab,kw OR (Furans):ti,ab,kw

#29. #25 OR #26 OR #27 OR #28

#30.(Critical* Care):ti,ab,kw OR (intensive care):ti,ab,kw OR (intensive ill*):ti,ab,kw OR (Intensive Care Unit*):ti,ab,kw OR (Critical* ill*):ti,ab,kw

#31. #10 OR #20 OR 29

#32.#24 OR #30

#33.#31 AND #32

## Table S1. The reasons for exclusion of ineligible studies

| **Reasons** | **References** |
| --- | --- |
| Non-randomized controlled trial (12 trials) | Mesquita/2018[s1]; Cohen/2017[s2]; Krzych/2017[s3]; Grindlinger/2016[s4]; Lee/2016[s5]; Murata/2015[s6]; Barletta/2014[s7]; Barletta/2014[s8]; Danziger/2013[s9]; Sankaranarayanan/2010[s10]; Ojiako/2008[s11]; Enns/2004[s12] |
| The subjects are not critically ill patients admitted to ICU (7 trials) | Hamai /2018[s13]: Bedridden hospitalized patients who were not admitted to the ICU  Wu/2011[s14]: Patients with acute coronary syndromes who were not admitted to the ICU  Goel/2016[s15]: Patients undergoing elective surgery who were not admitted to the ICU  Chan/1995[s16]: Patients suffering from nontraumatic neurosurgical lesions who were not admitted to the ICU  Cheadle/1985[s17]: Patients underwent major abdominal procedures who were not admitted to the ICU  Misra/2005[s18]: Patients with spontaneous intracerebral hemorrhage who were not admitted to the ICU  Schiessel/1981[s19]: Patients after renal transplantation who were not admitted to the ICU |
| Review article (3 records) | Lee /2017[s20]; Buendgens /2017[s21]; Milanov /2006[s22]; |
| Irrelevant articles(8 trials) | Duceppe/2018[s23];Ovenden /2017[s24];Hammond /2017[s25];  Sinuraya/2017[s26];Radev/2017[s27];Hammond/2015[s28]; Foroutan/2015[s29]; Dabiri/2015[s30] |
| Article without full-text  (1 trial) | Soria /1985[s31] (Article in Spanish without English abstract, we failed to obtain the full-text from the authors, so we excluded this article from our analysis) |
| PPI vs. H2RA  (27 trials) | Alikiaii /2017[s32]; Kahn/2015[s33]; MacLaren/2014 [s34];  MacLaren/2014 [s35]; Parri/2013[s36]; Tabeefar/2012[s37];  Brophy/2010[s38]; Madl/2008[s39]; Tofil/2008[s40];  Kodlyanskaya/2007[s41];Conrad/2005[s42];Kalaghchi/2004[s43];  Laine/2004[S44];Luo/2018[s45]; Bashar/2013[s46];  Fink/2013[s47]; Fogas/2013[s48]; Hata/2005[s49];  Lee/2004[s50];Levy/1997[s51];Pan/2004[s52];  Phillips/1998[s53]; Risaliti/1993[s54]; Solouki/2009[s55];  Solouki/2009[s56]; Somberg/2008[s57]; Wee/2013[s58] |
| H2RA vs. sucralfate  (14 trials) | Cook/1998[s59]; Cannon/1987[s60]; Fabain/1993[S61];  Harlaftis/1997[s62]; Laggner/1988[s63]; Laggner/1989[s64];  Mustafa/1995[s65]; Pickworth/1993[s66];Prakash/2008[s67];  Prod'hom/1994[s68]; Ryan/1993[s69]; Simms/1991[s70];  Thomason/1996[s71]; Tryba/1985[s72] |
| PPI vs. sucralfate  (2 trials) | Khorvash/2014[s73]; Kotlyanskaya/2008[s74] |
| Sucralfate vs. control group  (2 trials) | Eddleston /1994[s75]; Labattut/1992[s76] |

**SUPPLEMENTARY REFERENCE**

1. Mesquita WJ, Kline CM, Vernier P, Ledgerwood AM, Lucas CE. Failure of gastric prophylaxis against stress bleeding reflects inadequate pH control. Surgery. 2018;164(4):733-737.
2. Cohen ME, Hathway JM, Salmasian H, Liu J, Terry M, Abrams JA, et al. Prophylaxis for Stress Ulcers With Proton Pump Inhibitors Is Not Associated With Increased Risk of Bloodstream Infections in the Intensive Care Unit. Clin Gastroenterol Hepatol. 2017;15(7):1030-1036.
3. Krzych ŁJ, Łój P, Nowak T, Kazura W, Knapik P. Short-term proton pump inhibitor treatment may cause hypomagnesaemia in critically ill patients - a pilot study. Acta Biochim Pol. 2017;64(3):499-501.
4. Grindlinger GA, Cairo SB, Duperre CB. Pneumonia prevention in intubated patients given sucralfate versus proton-pump inhibitors and/or histamine II receptor blockers. J Surg Res. 2016;206(2):398-404.
5. Lee J, Mark RG, Celi LA, Danziger J. Proton Pump Inhibitors Are Not Associated With Acute Kidney Injury in Critical Illness. J Clin Pharmacol. 2016;56(12):1500-1506.
6. Murata A, Ohtani M, Muramatsu K, Matsuda S. Effects of proton pump inhibitor on outcomes of patients with severe acute pancreatitis based on a national administrative database. Pancreatology. 2015;15(5):491-496.
7. Barletta J, Sclar D. Proton pump inhibitors and the risk for hospital-acquired clostridium difficile in ICU patients. Crit Care Med. 2014; 42(12): p A1459.
8. Barletta JF. Histamine-2-receptor antagonist administration and gastrointestinal bleeding when used for stress ulcer prophylaxis in patients with severe sepsis or septic shock. Ann Pharmacother. 2014;48(10):1276-81.
9. Danziger J, William JH, Scott DJ, Lee J, Lehman LW, Mark RG, et al. Proton-pump inhibitor use is associated with low serum magnesium concentrations. Kidney Int. 2013;83(4):692-9.
10. Sankaranarayanan J, Olsen KM, Reardon T, Hays EM, Orwe LA. Factors associated with the use of enteral and paraenteral proton pump inhibitor use in critically ill patients. Value in Health. 2010; 13(3):A75.
11. Ojiako K, Shingala H, Schorr C, Gerber, DR. Famotidine Versus Pantoprazole for Preventing Bleeding in the Upper Gastrointestinal Tract of Critically Ill Patients Receiving Mechanical Ventilation. Am J Crit Care.2008;17(2): 142-147.
12. Enns R, Andrews CN, Fishman M, Hahn M, Atkinson K, Kwan P, et al. Description of prescribing practices in patients with upper gastrointestinal bleeding receiving intravenous proton pump inhibitors: a multicentre evaluation. Can J Gastroenterol. 2004;18(9):567-71.
13. Hamai K, Iwamoto H, Ohshimo S, Wakabayashi Y, Ihara D, Fujitaka K, et al. Use of proton pump inhibitors is associated with increased mortality due to nosocomial pneumonia in bedridden patients receiving tube feeding. Geriatr Gerontol Int. 2018;18(8):1215-1218.
14. Wu H, Jing Q, Wang J, Guo X. Pantoprazole for the prevention of gastrointestinal bleeding in high-risk patients with acute coronary syndromes. J Crit Care. 2011;26(4):434.e1-6.
15. Goel C, Anand LK, Gombar KK. Comparative evaluation of single dose intravenous pantoprazole and ranitidine on gastric pH and volume: A double blind study. Journal of Anaesthesiology Clinical Pharmacology. 2006;22(2):145-149.
16. Chan KH, Lai EC, Tuen H, Ngan JH, Mok F, Fan YW, et al. Prospective double-blind placebo-controlled randomized trial on the use of ranitidine in preventing postoperative gastroduodenal complications in high-risk neurosurgical patients. J Neurosurg. 1995;82(3):413-7.
17. Cheadle WG, Vitale GC, Mackie CR, Cuschieri A. Prophylactic postoperative nasogastric decompression. A prospective study of its requirement and the influence of cimetidine in 200 patients. Ann Surg. 1985;202(3):361-6.
18. Misra UK, Kalita J, Pandey S, Mandal SK, Srivastava M. A randomized placebo controlled trial of ranitidine versus sucralfate in patients with spontaneous intracerebral hemorrhage for prevention of gastric hemorrhage. J Neurol Sci. 2005;239(1):5-10.
19. Schiessel R, Starlinger M, Wolf A, Pinggera W, Zazgornik J, Schmidt P, et al. Failure of cimetidine to prevent gastroduodenal ulceration and bleeding after renal transplantation. Surgery. 1981;90(3):456-8.
20. Lee P, Fike DS, Hall R, Pass S, Alvarez C. Do the types and routes of proton pump inhibitor treatments affect Clostridium difficile in ICU patient? Pharmacotherapy.2017;37(12): E201.
21. Buendgens L, Tacke F. Do we still need pharmacological stress ulcer prophylaxis at the ICU? J Thorac Dis. 2017;9(11):4201-4204.
22. Milanov St, Georgiev G, Geube M, Milanov M. Proton pump inhibitors and stress-related mucosal disease in critically ill. Anaesthesiology and Intensive Care.2016;33(5):3-12.
23. Duceppe E, Yusuf S, Tandon V, Rodseth R, Biccard BM, Xavier D, et al. Design of a Randomized Placebo-Controlled Trial to Assess Dabigatran and Omeprazole in Patients with Myocardial Injury after Noncardiac Surgery (MANAGE). Can J Cardiol. 2018;34(3):295-302.
24. Ovenden C, Plummer MP, Selvanderan S, Donaldson TA, Nguyen NQ, Weinel LM, et al. Occult upper gastrointestinal mucosal abnormalities in critically ill patients. Acta Anaesthesiol Scand. 2017;61(2):216-223.
25. Hammond DA, Kathe N, Shah A, Martin BC. Cost-Effectiveness of Histamine2 Receptor Antagonists Versus Proton Pump Inhibitors for Stress Ulcer Prophylaxis in Critically Ill Patients. Pharmacotherapy. 2017;37(1):43-53.
26. Sinuraya RK, Gondodiputro S, Djuhaeni, H. Proton pump inhibitors for stress ulcer bleeding prophylaxis in critically ill patients: A cost analysis study. Asian Journal of Pharmaceutical and Clinical Research.2017;10(Special Issue may): 128-130.
27. Radev RN, Radev VR, Stefanovski PH, Vateva YaD, Malkodanski ITs, Bogdanov SlH, Yolova S. Prophylaxis of stres-ulcer in critically ill patients with acute gastro intestinal damage. Anaesthesiology and Intensive Care.2017;46(3): 40-45.
28. Hammond D, Shah A, Martin B. Cost-effectiveness of H2RAS versus ulcer prophylaxis in critically ill patients. Crit Care Med.2015;431(12):
29. Foroutan N, Foroutan A. Finacial impacts of using omeprazole oral suspension for preventing upper gastrointestinal bleeding early after intensive care admission. Value in Health.2015;18(3):A224.
30. Dabiri Y, Fahimi F, Jamaati H, Hashemian SMR. The comparison of extemporaneous preparations of omeprazole, pantoprazole oral suspension and intravenous pantoprazole on the gastric pH of critically ill-patients. Indian Journal of Critical Care Medicine.2015;19(1): 21-26.
31. Soria F, Jr., Perez HR, Mathier VH. Incidence of acute gastroduodenal lesions in patients with intracranial lesions. Prophylactic value of treatment with ranitidine. Rev Fac Cien Med Univ Nac Cordoba. 1985;43(1):32-5. [Article in Spanish]
32. Alikiaii B, Kashefi P, Abbasi S, Askari-Barzani E. Comparison of serum level of magnesium in patients received pantoprazole or ranitidine in intensive care unit. Journal of Isfahan Medical School.2017;35(432): 615-621.
33. Kahn J, Kopterides P. Histamine-2 receptor antagonists versus proton pump inhibitors for stress ulcer prophylaxis in the ICU. F1000 Research. 2015; 4:1291.
34. MacLaren R, Reynolds PM, Allen RR. Histamine-2 receptor antagonists vs proton pump inhibitors on gastrointestinal tract hemorrhage and infectious complications in the intensive care unit. JAMA Intern Med. 2014;174(4):564-74.
35. MacLaren R, Campbell J. Cost-effectiveness of histamine receptor-2 antagonist versus proton pump inhibitor for stress ulcer prophylaxis in critically ill patients*. Crit Care Med. 2014;42(4):809-15.
36. Parri MS, Gianetti J, Dushpanova A, Della Pina F, Saracini C, Marcucci R, et al. Pantoprazole significantly interferes with antiplatelet effect of clopidogrel: Results of a pilot randomized trial. International Journal of Cardiology.2013;167(5): 2177-2181.
37. Tabeefar H, Beigmohammadi MT, Javadi MR, Abdollahi M, Mahmoodpoor A, Ahmadi A, et al. Effects of Pantoprazole on Systemic and Gastric Pro- and Anti-inflammatory Cytokines in Critically Ill Patients. Iran J Pharm Res. 2012; 11(4): 1051–1058.
38. Brophy GM, Brackbill ML, Bidwell KL, Brophy DF. Prospective, randomized comparison of lansoprazole suspension, and intermittent intravenous famotidine on gastric pH and acid production in critically ill neurosurgical patients. Neurocrit Care. 2010;13(2):176-81.
39. Madl C, Kitzberger R, Warszawska J, Holzinger U, Hammer J, Stauber R, et al. Effect of intravenous esomeprazole versus ranitidine on gastric pH in critically ill patients - a prospective, randomized, double-blind, multicenter study. WIENER KLINISCHE WOCHENSCHRIFT.2008;120(1-2):A8.
40. Tofil NM, Benner KW, Fuller MP, Winkler MK. Histamine 2 receptor antagonists vs intravenous proton pump inhibitors in a pediatric intensive care unit: a comparison of gastric pH. J Crit Care. 2008 Sep;23(3):416-21.
41. Kodlyanskaya A, Luka B, Mukherji R, Shukla M, Vajinder V, Cohen H. A comparison trial of lansoprazole disintegrating tablet, lansoprazole suspension or ranitidine for stress ulcer prophylaxis in critically ill patients. Crit Care Med.2007;35s(12):A194.
42. Conrad SA, Gabrielli A, Margolis B, Quartin A, Hata JS, Frank WO, et al. Randomized, double-blind comparison of immediate-release omeprazole oral suspension versus intravenous cimetidine for the prevention of upper gastrointestinal bleeding in critically ill patients. Crit Care Med. 2005;33(4):760-5.
43. Kalaghchi B, Ghandi M, Hoque ME, Henderson T, Adams RG, Thomas AV, et al. Randomized comparison of gastric pH control with ranitidine, esomeprazole and pantoprazole for stress ulcer prophylaxis in critically ill patients. Gastroenterology.2004;126(4):A445.
44. Laine L, Margolis B, Bagin RG, Rock J, Hepburn B, Frank WO. Double-blind trial of omeprazole-immediate release oral suspension (OME-IR SUSP) vs. intravenous cimetidine (IV CIM) for prevention of upper gastrointestinal (UGI) bleeding in critically ill patients. Gastroenterology. 2004;126(4):A77-A78.
45. Lou W, Xia Y, Xiang P, Zhang L, Yu X, Lim S, et al. Prevention of upper gastrointestinal bleeding in critically ill Chinese patients: a randomized, double-blind study evaluating esomeprazole and cimetidine. Current Medical Research and Opinion. 2018;34(8):1449-1455.
46. Bashar FR, Manuchehrian N, Mahmoudabadi M, Hajiesmaeili MR, Torabian S. Effects of ranitidine and pantoprazole on ventilator-associated pneumonia: a randomized double-blind clinical trial. Tanaffos.2013; 12(2):16–21.
47. Fink M, Karlstadt RG, Maroko RT, Field B. Intravenous pantoprazole (IVP) and continuous infusion cimetidine (C) prevent upper gastrointestinal bleeding (UGIB) regardless of APSII score (APACHE II) in high risk intensive care unit (ICU) patients. Gastroenterology.2013; 124:A625–A626.
48. Fogas JF, Kiss KK, Gyura FG, Tobias ZT, Molnar ZM. Effects of proton pump inhibitor versus H2-receptor antagonist stress ulcer prophylaxis on ventilator-associated pneumonia: a pilot study. 33rd International Symposium on Intensive Care and Emergency Medicine Brussels Belgium.2013;17:S150–S151.
49. Hata M, Shiono M, Sekino H, Furukawa H, Sezai A, Iida M, et al. Prospective randomized trial for optimal prophylactic treatment of the upper gastrointestinal complications after open heart surgery. Circ J.2005; 69:331–334.
50. Lee T-H, Hung F-M, Yang L-H. Comparison of the efficacy of esomeprazole and famotidine against stress ulcers in a neurosurgical intensive care unit. Adv Dig Med.2004; 1:50–53.
51. Levy MJ, Seelig CB, Robinson NJ, Ranney JE. Comparison of omeprazole and ranitidine for stress ulcer prophylaxis. Dig Dis Sci.1997; 42:1255–1259.
52. Pan X, Zhang W, Li Z, Xu G, Yin N, Wang D, et al. The preventive effects of rabeprazole on upper gastrointestinal tract hemorrhage in patients with severe acute pancreatitis. Chin J Gastroenterol.2004; 9:30–32.
53. Phillips JO, Metzler MH, Huckfeldt RE, Olsen K. A multicenter, prospective, randomized clinical trial of continuous infusion IV ranitidine vs. omeprazole suspension in the prophylaxis of stress ulcers. Crit Care Med.1998; 26:101A.
54. Risaliti A, Terrosu G, Uzzau A, Petri R, Intini S, Carcoforo P, et al. Intravenous omeprazole vs ranitidine in the prophylaxis of stress ulcers. Acta Chirurgica Italica.1993;49:397–401.
55. Solouki M, Mar’ashian SM, Koochak M, Nasiri A, Mokhtari M, Amirpour A. Ventilator-associated pneumonia among ICU patients receiving mechanical ventilation and prophylaxis of gastrointestinal bleeding. Iran J Clinical Infect Dis.2009;4:177–180.
56. Solouki M, Marashian SM, Kouchak M, Mokhtari M, Nasiri E. Comparison between the preventive effects of ranitidine and omeprazole on upper gastrointestinal bleeding among ICU patients. Tanaffos.2009;8:37–42.
57. Somberg L, Morris J Jr, Fantus R, Graepel J, Field BG, Lynn R, et al. Intermittent intravenous pantoprazole and continuous cimetidine infusion: effect on gastric pH control in critically ill patients at risk of developing stress-related mucosal disease. J Trauma.2008;64:1202–1210.
58. Wee B, Liu CH, Cohen H, Kravchuk S, Reddy K, Mukherji R. IV Famotidine vs. IV Pantoprazole for stress ulcer prevention in the ICU: a prospective Study. Crit Care Med.2013; 41:A181.
59. Cook D, Guyatt G, Marshall J, Leasa D, Fuller H, Hall R, et al. A comparison of sucralfate and ranitidine for the prevention of upper gastrointestinal bleeding in patients requiring mechanical ventilation. Canadian Critical Care Trials Group. N Engl J Med. 1998;338(12):791-7.
60. Cannon LA, Heiselman D, Gardner W, Jones J. Prophylaxis of upper gastrointestinal tract bleeding in mechanically ventilated patients. A randomized study comparing the efficacy of sucralfate, cimetidine, and antacids. Arch Intern Med. 1987;147(12):2101-6.
61. Fabain TC, Boucher BA, Croce MA, Kuhl DA, Janning SW, Coffey BC, et al. Pneumonia and stress ulceration in severely injured patients. A prospective evaluation of the effects of stress ulcer prophylaxis. Arch Surg. 1993;128(2):185-91; discussion 91-2.
62. Harlaftis N, Basdanis G, Papapolychroniadis C, Prousalidis J, Apostolidis S, Kosmidis C, et al. Nosocomial pneumonia in mechanically ventilated patients during stress ulcer prophylaxis with sucralfate and ranitidine. Hellenic journal of gastroenterology. 1997;10:230-5.
63. Laggner AN, Lenz K, Graninger W, Gremmel F, Grimm G, Base W, et al. Prevention of stress hemorrhage in an internal medicine intensive care station: sucralfate versus ranitidine. Der Anaesthesist. 1988;37(11):704-10.
64. Laggner AN, Lenz K, Base W, Druml W, Schneeweiss B, Grimm G. Prevention of upper gastrointestinal bleeding in long-term ventilated patients: Sucralfate versus ranitidine. The American journal of medicine. 1989;86(6):81-4.
65. Mustafa NA, Aktürk G, Özen I, Köksal I, Erciyes N, Solak M. Acute stress bleeding prophylaxis with sucralfate versus ranitidine and incidence of secondary pneumonia in intensive care unit patients. Intensive care medicine. 1995;21(3):287.
66. Pickworth KK, Falcone RE, Hoogeboom JE, Santanello SA. Occurrence of nosocomial pneumonia in mechanically ventilated trauma patients: a comparison of sucralfate and ranitidine. Crit Care Med. 1993;21(12):1856-62.
67. Prakash S, Rai A, Gogia AR, Prakash S. Nosocomial pneumonia in mechanically ventilated patients receiving ranitidine or sucralfate as stress ulcer prophylaxis. Indian Journal of Anaesthesia. 2008;52(2):179.
68. Prod'hom G, Leuenberger P, Koerfer J, Blum A, Chiolero R, Schaller M-D, et al. Nosocomial pneumonia in mechanically ventilated patients receiving antacid, ranitidine, or sucralfate as prophylaxis for stress ulcer: a randomized controlled trial. Annals of internal medicine. 1994;120(8):653-662.
69. Ryan P, Dawson J, Teres D, Celoria G, Navab F. Nosocomial pneumonia during stress ulcer prophylaxis with cimetidine and sucralfate. Arch Surg. 1993;128(12):1353-1357.
70. Simms HH, DeMaria E, McDonald L, Peterson D, Robinson A, Burchard KW. Role of gastric colonization in the development of pneumonia in critically ill trauma patients: results of a prospective randomized trial. J Trauma. 1991;31(4):531-6; discussion 6-7.
71. Thomason MH, Payseur ES, Hakenewerth AM, Norton HJ, Mehta B, Reeves TR, et al. Nosocomial pneumonia in ventilated trauma patients during stress ulcer prophylaxis with sucralfate, antacid, and ranitidine. J Trauma. 1996;41(3):503-508.
72. Tryba M, Zevounou F, Torok M, Zenz M. Prevention of acute stress bleeding with sucralfate, antacids, or cimetidine: a controlled study with pirenzepine as a basic medication. The American journal of medicine. 1985;79(2):55-61.
73. Khorvash F, Abbasi S, Meidani M, Dehdashti F, Ataei B. The comparison between proton pump inhibitors and sucralfate in incidence of ventilator associated pneumonia in critically ill patients. Adv Biomed Res.2014;3:52.
74. Kotlyanskaya A, Luka B, Mukherji R. A comparison of lansoprazole disintegrating tablet, lansoprazole suspension or ranitidine for stress ulcer prophylaxis in critically ill patients. Crit Care Med. 2008;7:A194.
75. Eddleston JM, Pearson RC, Holland J, Tooth JA, Vohra A, Doran BH. Prospective endoscopic study of stress erosions and ulcers in critically ill adult patients treated with either sucralfate or placebo. Crit Care Med.1994; 22:1949–1954.
76. Labattut AG, Santolalla PM, De Andres AP, Ortigosa AM, Del Mar M, Serrano G, Gimeno OL. Efficacy of sucralfate in the prevention of upper gastrointestinal stress bleeding in intensive care patients: comparison vs a control group. Clin Intensive Care.1992;3:19–25.

## Table S2. The detailed characteristics of all included trials

| First author /Publication year | Design (location) | Sample size | Population | Setting | Intervention group | Control group | APACHE II /SAPS II score | | | Definition of GI bleeding | Enteral nutrition |
| --- | --- | --- | --- | --- | --- | --- | --- | --- | --- | --- | --- |
|  |  |  |  |  |  |  | Intervention group | Control group | |  |  |
| Alhazzani  /2017[24] | Multi-center  (Canada, Saudi Arabia, Australia) | 91 | ICU patients anticipated  to receive MV for ≥ 48 hours | Mixed ICU | Pantoprazole 40 mg once daily , IV | Placebo | 21 (17–26) | 21.5 (14–27) | | Overt GI bleeding plus one of these features: a spontaneous drop of SBP or DBP ≥ 20 mm Hg within 24 h of upper GI bleeding; an orthostatic increase in pulse rate ≥ 20 beats/min and a decrease in SBP ≥ 10 mm Hg, a decrease in hemoglobin ≥ 2 g/dL in 24 h or transfusion of ≥ 2 units of packed RBCs within 24h of bleeding | Most patients (81/91) received |
| Apte/1992[25] | Single-center  (India) | 34 | Tracheotomised  patients with tetatus | Medical ICU | Ranitidine 50 mg q6h, IV | No prophylaxis | - | | - | No clear definition | All patients received |
| Basso/1981[8] | Single-centre (Italy) | 116 | Patients after surgery with high risk for GI bleeding | surgical ICU | Cimetidine 200 mg q6h, IV or orally, at least 10 days | No prophylaxis | - | | - | Clinical signs related to GI hemorrhage (hematemesis, melena, blood in the nasogastric tube or the stool or changes in hematocrit) | No |
| Ben-Menachem/1994[26] | Single-centre (USA) | 200 | ICU patients with high risk for GI bleeding | Medical ICU | Cimetidine loading dose of 300 mg IV and infusion titrated to maintain gastric pH >4 | No prophylaxis | 18.0 ± 8.0 | | 16.5 ± 6.9 | Substantial GI hemorrhage plus one of these features: 1)persistent hematemesis (red blood or guaiac-positive "coffee grounds" that did not clear with 1.5 L saline lavage; 2) 3-point decrease in hematocrit during 24 h accompanied by red blood or guaiac-positive "coffee grounds" material that cleared with lavage, or melena, or three guaiac-positive stools without evidence of lower gastrointestinal bleed; and 3) any unexplained 6-point decrease in hematocrit during a 48 h period. | Most patients (129/200)  received |
| van den Berg /1985[27] | Single-centre (Netherlands) | 28 | ICU patients on assisted ventilation | Mixed ICU | Cimetidine 20 mg/  Kg per 24 h, IV | Placebo | - | | - | No clear definition | Most patients (17/28)  received |
| Burgess/1995[28] | Single-centre (USA) | 34 | Adults with severe head injury and a GCS score ≤10 | Surgical ICU | Ranitidine 6.25 mg/h IV for  up to 72 h | Placebo | - | | - | 5% decrease from baseline in hematocrit occurring at least 8 h after study drug initiation plus any of the following signs: hematemesis, hematochezia, bright red blood per NG tube or "coffee ground" nasogastric tube aspirates. | No |
| Calvet/1998[29] | Single-centre (Spain) | 25 | Critically ill patients requiring MV, arterial catheter and NG tube | Mixed ICU | Ranitidine 50 mg, IV (one dose) | Placebo | 15.2 ± 5.3 | | 18 ± 9.5 | No clear definition | No |
| Darlong/2003[9] | Single centre  (India) | 31 | ICU patients needed MV and NG tube in situ | Mixed ICU | Ranitidine 50 mg q8h, IV | No prophylaxis | - | | - | No clear definition | No |
| El-Kersh/2018[30] | Multi-center (2 hospitals in USA) | 102 | ICU patients needed MV for >48 h with no contraindications to EN | Medical ICU | Pantoprazole 40 mg once  Daily, IV | Placebo | 41.0  (34.5–53.0) | | 44.0  (34.0–54.0) | A 3-point decrease in hematocrit within a 24 h period, or an unexplained 6-point decrease in hematocrit in a 48 h period with clinical signs of overt GI bleeding (coffee-ground aspirate in nasogastric tube or coffee-ground emesis, bloody secretions in NG tube or hematemesis, melena or hematochezia) | All patients received |
| Friedman/1982[31] | Single-centre  (USA) | 25 | ICU patients received MV for <12 h | Medical ICU | Cimetidine 300 mg q6h, IV | Placebo | - | | - | Overt GI bleeding was defined as the presence of fresh or old blood in the NG aspirate which failed to clear with saline lavage in 15 min, or as melena | No |
| Groll/1986[32] | Single-centre  (Canada) | 221 | ICU patients | Mixed ICU | Cimetidine 300 mg q6h, IV | Placebo | - | | - | Criteria for bleeding were:  (i)haematemesis or gastric aspirate >50ml fresh blood  (ii) melaena or fresh blood per rectum with an upper source verified by endoscopy if the gastric aspirate was clear  (iii) a fall in haemoglobin level >2 g/dl in a 24 h period associated with either 4+ occult blood in stools, or coffee ground gastric drainage of at least 100 ml | No |
| Gursoy/2008[33] | Single-centre  (Turkey) | 75 | Adult trauma, general surgical, medical patients requiring MV | Mixed ICU | Arm1: Omeprazole 20 mg capsule (n=15);  Arm2: Pantoprazole 40 mg tablet (n=15);  Arm3: Esomeprazole 20 mg tablet (n=15) ;  Arm4: Rabeprazole 20 mg tablet (n=15);  All were administered nasogastrically in 100 mL of saline | Placebo | Arm1:15(12-17)  Arm2:14(12–17)  Arm3:14(12–16)  Arm4:13(11–17) | | 15 (12–18) | No clear definition | No |
| Halloran/1980[10] | Single-centre  (USA) | 50 | Patients with severe head injury | Surgical ICU | Cimetidine 300 mg q4h, IV | Placebo | - | | - | Bright red blood or a 4 plus positive guaiac in the gastric aspirate for three consecutive 8 h periods; Bleeding was considered marked when transfusion of 2 or more units of whole blood. | Yes |
| Hanisch/1998[34] | Single-centre  (Germany) | 114 | ICU patients with MV >48 h | Surgical ICU | Ranitidine 50 mg IV, 3 doses | Placebo | Mean(range)  19(2–30) | | Mean(range)  18(1–28) | Bright red blood via gastric tube or melena combined with hemodynamic changes (SBP <100 mmHg, tachycardia >100 beats/min) and requirement of blood transfusion (fall in hemoglobin >2 g/dL within 24 h) and endoscopic identification of bleeding site and activity | No |
| Kantorova/2004[35] | Single-centre  (Czech Republic) | 218 | ICU patients with MV >48h or have coagulopathy or have a NG tube in place | Surgical ICU | Arm 1: Omeprazole 40 mg once daliy, IV (n=74)  Arm 2: Famotidine  40 mg q12h, IV (n=71) | Placebo | Arm 1: 17.5±8.6  Arm 2: 19.1±9.3 | | 18.1±9.3 | Overt GI bleeding plus at least one of the following: 1) drop of SBP ≥20 mmHg or increase in pulse rate ≥20 beats/min within 24 h of upper GI bleeding; 2) decrease in hemoglobin ≥2 g/dL | Yes (81/218) |
| Karlstadt/1990[11] | Multi-centre  (USA) | 87 | ICU patients with high risk for GI bleeding | Mixed ICU | Cimetidine 300 mg loading dose, followed by infusion at 50 mg/every hour | Placebo | - | | - | Clinically significant GI bleeding was defined by one of the following criteria: (1) hematemesis or the presence of more than 10 ml of bright red blood in a single aspirate; (2) melena or hematochezia; (3) the presence of “coffee grounds”; positive for hemoglobin by Gastroccult in the NG aspirate on each of 3 consecutive 6-hourly observations (over 12 h) and a 1-gm decrease in hemoglobin over 24 h; or (4) Gastroccult-positive “coffee grounds” in aspirate that did not clear with lavage. | No |
| Krag/2018[18] | Multi-center (Denmark, Finland,Netherlands, Norway, Switzerland, and UK) | 3298 | ICU patients with at least one high risk for GI bleeding | Mixed ICU | Pantoprazole 40 mg as a  single bolus once daily, IV | Placebo | 49 (39–59) | | 48 (37–59) | Overt GI bleeding plus at least one of the following features:1) in the absence of other causes, in the ICU; 2) a spontaneous decrease in SBP,MAP, or DBP of 20 mm Hg or more; 3) initiation of treatment with a vasopressor or a 20% increase in vasopressor dose; 4) a decrease in hemoglobin of at least 2 g per deciliter or transfusion of 2 or more units of packed red cells | Most patients (1885/3291)received |
| Lin/2016[36] | Single-centre  (China) | 120 | ICU patients being  weaned from MV | Mixed ICU | Lansoprazole OD 30 mg once daily | No  prophylaxis | 21.3 ±6.7 | | 19.9 ±6.9 | Apparent GI bleeding was defined as follows: (1) a coffee ground substance from the NG aspirate ≥60 mL; (2) fresh blood from the NG tube; or (3) passage of tarry stool.  Clinically significant UI bleeding: UGI bleeding with hemoglobin level decrease ≥2 gm/dL or in need of a blood transfusion of >2 units | All patients received |
| Liu/2013[37] | Single-centre  (China) | 165 | Patients hospitalized at the neurosurgical ICU with CT-proven ICH within 72 h of ictus and negative results for gastric occult blood testing | Surgical ICU | Arm 1: Omeprazole 40 mg q12h, IV (n=58)  Arm 2: Cimetidine 300 mg q6h, IV (n=54) | Placebo | - | | - | Hematemesis, aspiration of coffeeground material from NG tube, or melena which was proven by positive results of gastric occult blood testing or fecal occult blood testing, with or without hemodynamic instability resulting from gross bleeding that needed transfusion | No |
| Macdougall/1977[7] | Single-centre  (UK) | 62 | Fulminant hepatic  failure | Medical ICU | Metiamide 150 mg/  h (n = 10) or cimetidine  100 mg/h (n =16), IV | No prophylaxis | - | | - | No clear definition | No |
| Martin/1993[12] | Multi-centre  (USA) | 131 | ICU patients at least one high risk for GI bleeding with a NG tube in place | Mixed ICU | Cimetidine 300 mg loading dose, followed by 50 mg/h IV for up to 7 days | Placebo | 16.9±7.8 | | 15.1±5.8 | 1.Hematemesis or bright red blood that did not clear after NG tube adjustment or 5-10 min lavage;2. Or persistent coffeeground material(8 h) and/or accompanied by a 5% decrease in hematocrit | No |
| Metz/1993[13] | Multi-centre  (USA) | 167 | Patients with severe head injury  (GCS ≤10) | Surgical ICU | Ranitidine 6.25 mg/h. IV for up to 5 days | Placebo | - | | - | 1. a presence of Gastroccult positive NG tube drainage 2. presence bright red blood per NG tube 3. Hematemesis 4. Hemoccult positive stool 5. Melena 6. hematochezia | No |
| Nourian/2018[38] | Single-centre  (Iran) | 50 | Critically ill adults with indications to receive SUP | Medical ICU | Ranitidine 50 mg q8h as slow intravenous injection | No prophylaxis | 18.28 ± 5.31 | | 17.12 ± 5.43 | Overt GI bleeding (coffee ground gastric secretions, hematemesis or melena) | All patients received |
| Peura/1985[40] | Single-centre  (USA) | 39 | Patients admitted to ICU no less than 5 days' | Medical ICU | Cimetidine 300 mg q6h, IV for 3–14 days | Placebo | - | | - | No clear definition | Yes |
| Powell/1993[39] | Single-centre  (UK) | 41 | Scheduled coronary artery bypass graft patients in ICU | Surgical ICU | Arm1: Ranitidine 50 mg q8h, IV (n=11)  Arm2: Omeprazole 80 mg loading dose followed by 40 mg q8h by bolus IV (n=10)  Arm3: Omeprazole 80 mg loading dose followed by 40 mg q8h by infusion (n=10) | Placebo | - | | - | No clear definition | No |
| Reusser/1990[41] | Single-centre  (Switzerland) | 40 | ICU patients at least one high risk for GI bleeding | Surgical ICU | Ranitidine 50 mg/ q8-6h, IV | No prophylaxis | - | | - | Bright red bleeding via NG tube; melena; or decrease of Hgb blood level >2g/L within 24h, associated with a positive stool guaiac test or with gastric drainage of >100ml of coffee groung material | No |
| Ruiz-Santana/1991[42] | Single-centre  (Spain) | 49 | ICU patients with no less than 6 days'of MV | Mixed ICU | Ranitidine 50 mg q8h, IV | No prophylaxis | 16±6 | | 16±5 | Coffee ground material, bloody aspirate, hematemesis or melena | All patients received |
| Selvanderan/2016[43] | Single-centre (Australia) | 214 | Mechanically ventilated critically ill patients suitable for enteral nutrition. | Mixed ICU | Pantoprazole 40 mg once daily IV for a maximum of  14 days | Placebo | APACHE III score 66±26 | | APACHE III score 66±28 | An episode of overt bleeding (hematemesis, bloody gastric aspirate, melena, or hematochezia), accompanied by at least one of the following: 1) a reduction in MAP of ≥ 20 mmHg within 24 h in the absence of another cause, 2) a reduction in hemoglobin ≥ 20 g/L within 24 h, or 3) a need for endoscopy or surgery to achieve hemostasis | All patients received |
| Zinner/1981[44] | Multi-centre  (USA) | 200 | Expected ICU duration >48 h | Surgical ICU | Cimetidine 300 mg q6h, IV | No prophylaxis | - | | - | Incidence of upper GI bleeding defined as persistent guaiac 4+ positive NG aspirate, continuous for greater than 16 h even after NG lavage, bright red bleeding per NG tube or by emesis, and guaiac-positive stools and documented fall in haematocrit value | No |

The data were presented as mean± standard deviation or median (interquartile rang);

APACHE acute physiology and chronic health evaluation; SAPS Simplified Acute Physiology Score; GI gastrointestinal; NG nasogarstic; ICU intensive care unit; MV mechanical ventilation; SBP systolic blood pressure; DBP diastolic blood pressure; MAP mean arterial pressure; GCS Glasgow coma scale; CT computed tomography; ICH intracerebral hemorrhage.

## Table S3. The detailed data on clinical outcomes in all included trials

| First author /Publication year | Mortality  (death/total) | | Clinically important GI  bleeding (events/total) | | Overt GI bleeding (events/total) | | Pneumonia  (events/total) | | Clostridium difficile infection  (events/total) | | Duration of ICU stay (days)  [mean±SD/median(IQR)] | | Duration of MV (days) [mean±SD/median(IQR)] | |
| --- | --- | --- | --- | --- | --- | --- | --- | --- | --- | --- | --- | --- | --- | --- |
|  | IG | CG | IG | CG | IG | CG | IG | CG | IG | CG | IG | CG | IG | CG |
| Alhazzani/2017 | ICU mortality  11/49 10/42  In-hospital mortality  17/49 13/42 | | 3/49 2/42 | | 4/49 3/42 | | 10/49 6/42 | | 2/49 1/42 | | 12(8-23) 8.5(6-18) | | 9(5-17) 6.5(4-14) | |
| Apte/1992 | 11/16 7/18 | | — — | | 5/16 6/18 | | 13/16 9/18 | | — — | | — — | | — — | |
| Basso/1981 | - — | | - — | | 0/60 8/56 | | - — | | - — | | - — | | - — | |
| Ben-Menachem/1994 | ICU mortality  19/100 11/100  In-hospital mortality  28/100 19/100 | | 5/100 6/100 | | 5/100 6/100 | | 13/100 6/100 | | - — | | 4(2-9) 3(2-8) | | 8.1±11 7.9±9.6 | |
| van den Berg /1985 | - — | | - — | | 5/14 1/14 | | - — | | - — | | - — | | - — | |
| Burgess/1995 | 1/16 0/18 | | 0/16 5/18 | | 0/16 5/18 | | - — | | - — | | - — | | - — | |
| Calvet/1998 | 5/12 5/13 | | - — | | - — | | - — | | - — | | - — | | - — | |
| Darlong/2003 | - — | | - — | | 3/24 4/7 | | - — | | - — | | - — | | - — | |
| El-Kersh/2018 | In-hospital mortality  7/55 8/47 | | 1/55 1/47 | | 1/55 1/47 | | - — | | 1/55 3/47 | | 6(4-6.9) 7(3.5-11.5) | | 4(2.2-7) 5(3-8) | |
| Friedman/1982 | - — | | - — | | 1/11 5/14 | | - — | | - — | | - — | | - — | |
| Groll/1986 | 13/114 13/107 | | - — | | 6/114 11/107 | | - — | | - — | | - — | | - — | |
| Gursoy/2008 | Arm1: 1/15 2/15  Arm2: 2/15  Arm3: 2/15  Arm4: 1/15 | | - — | | - — | | - — | | - — | | Arm1:5.27±3.37  Arm2:6.87±3.13  Arm3:7.13±2.94  Arm4:6.53±5.54  CG: 6.67±4.88 | | - — | |
| Halloran/1980 | 8/26 10/24 | | 2/26 8/24 | | 5/26 18/24 | | - — | | - — | | - — | | - — | |
| Hanisch/1998 | 7/57 12/57 | | 3/57 2/57 | | 3/57 2/57 | | 10/57 12/57 | | - — | | Mean(range)  9.7(2-9.5) 12.6(2-58) | | Mean(range)  8.2(2-93) 10.2(2-55) | |
| Kantorova/2004 | ICU mortality  Arm1: 9/72 8/75  Arm2: 10/71  In-hospital mortality  Arm1: 14/72 13/75  Arm2: 11/71 | | Arm1: 1/72 1/75  Arm2: 2/71 | | Arm1: 1/72 1/75  Arm2: 2/71 | | Arm1: 8/72 5/75  Arm2: 7/71 | | - — | | Arm1: 7.7±7.3 8.6±11.3  Arm2: 10.1±9.8 | | Arm1:6.6±9.5 6.1±10.4  Arm2: 7.3±8.4 | |
| Karlstadt/1990 | 5/54 2/33 | | 1/54 7/33 | | 1/54 7/33 | | 1/54 0/33 | | - — | | - — | | - — | |
| Krag/2018 | 90-day mortality  510/1642 499/1640 | | 41/1644 69/1647 | | 88/1644 148/1647 | | 266/1644 266/1647 | | 19/1644 25/1647 | | - — | | - — | |
| Lin/2016 | 30-day mortality  2/60 0/60 | | 0/60 1/60 | | 0/60 5/60 | | 4/60 6/60 | | - — | | - — | | - — | |
| Liu/2013 | Arm1:17/58 20/53  Arm2:14/54 | | - — | | Arm1:9/58 24/53  Arm2:15/54 | | Arm1:14/58 8/53  Arm2:12/54 | | - — | | - — | | - — | |
| Macdougall/1977 | 20/26 31/36 | | - — | | 1/26 21/36 | | - — | | - — | | - — | | - — | |
| Martin/1993 | 30-day mortality  8/65 7/66 | | - — | | 9/65 22/66 | | 0/56 4/61 | | - — | | Median(range)  4(1-91) 4(1-133) | | - — | |
| Metz/1993 | - — | | - — | | 3/86 15/81 | | - — | | - — | | - — | | - — | |
| Nourian/2018 | 28-day mortality  8/25 5/25 | | - — | | 1/25 1/25 | | 9/25 7/25 | | - — | | 15.19±12.81 13±11.98 | | 11.6±7.43 15.04±7.17 | |
| Peura/1985 | 7/21 7/18 | | - — | | 1/21 7/18 | | - — | | - — | | - — | | - — | |
| Powell/1993 | Arm1: 0/10 0/10  Arm2: 1/10  Arm3: 0/10 | | - — | | Arm1: 0/10 0/10  Arm2: 0/10  Arm3: 0/10 | | - — | | - — | | - — | | - — | |
| Reusser/1990 | In-hospital mortality  5/19 6/21 | | - — | | 0/19 0/21 | | - — | | - — | | Median(range)  13(6-40) 17(5-30) | | - — | |
| Ruiz-Santana/1991 | 7/19 7/30 | | - — | | 2/19 1/30 | | - — | | - — | | 16±7 19±9 | | - — | |
| Selvanderan/2016 | 90-day mortality  30/106 25/108 | | 0/106 0/108 | | 3/106 6/108 | | 12/106 8/108 | | 1/106 0/108 | | 6(3-11) 7(4-14) | | 21(10-25) 21(4-25) | |
| Zinner/1981 | 9/100 17/100 | | - — | | 14/100 20/100 | | - — | | - — | | - — | | - — | |

GI gastrointestinal; ICU intensive care unit; MV mechanical ventilation; SD standard deviation; IQR interquartile rang; IG Intervention group; CG control group

## Figure S1. Trial sequential analysis for the clinically important GI bleeding in trials with use of PPI


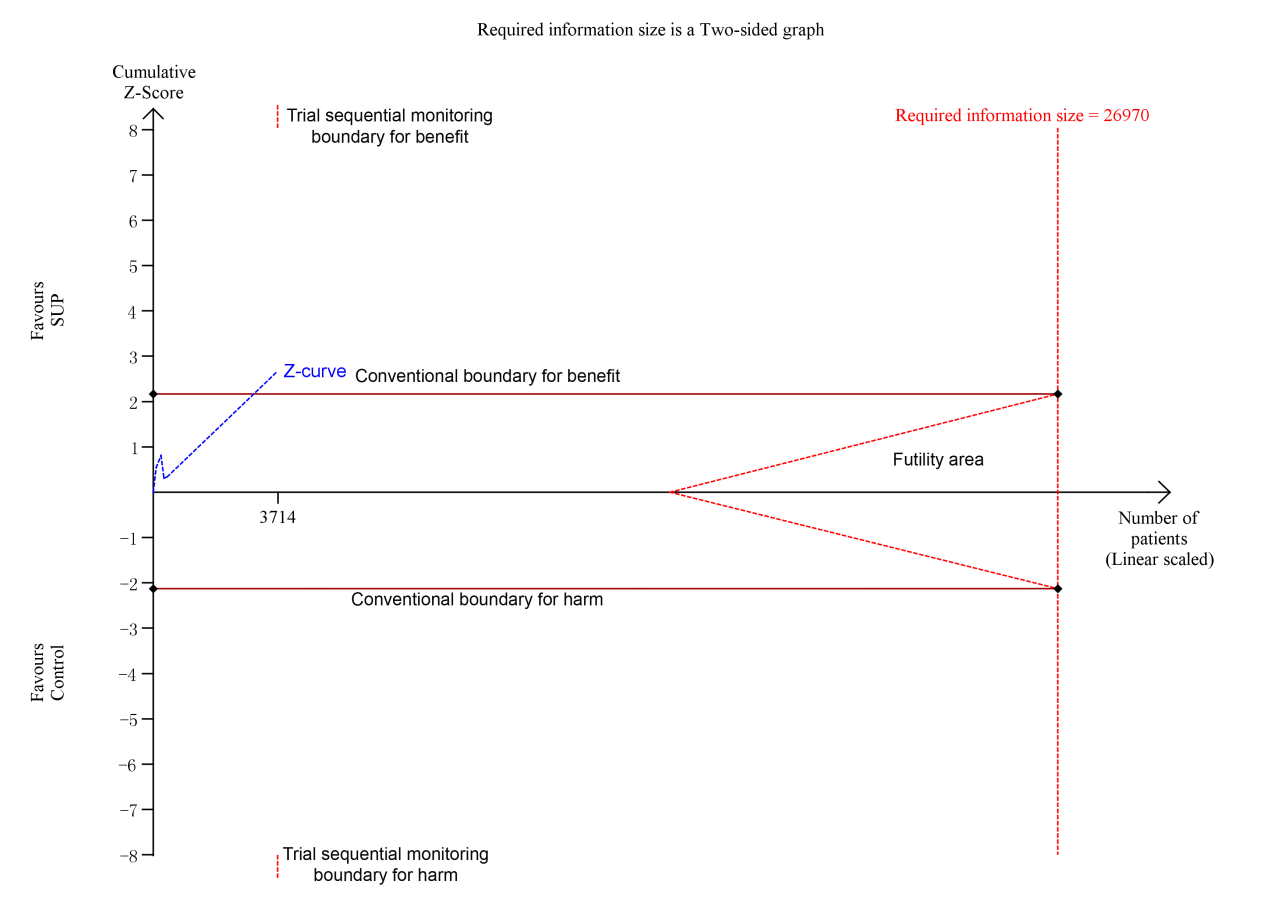


Trial sequential analysis for the clinically important GI bleeding was conducted using random-effects model in trials with use of PPI, with an adjusted type I error of 3.3%, power of 80%, D^2^ of 25% (the actual measured D^2^ was 0), and a relative risk reduction of 20% in control event proportion of 3.8%. The cumulative z-curve cross no boundaries, the required information size of 26970 patients are not reached, the TSA-adjusted 95% CI for an RR of 0.61 is 0.14 to 2.70.

## Figure S2. Trial sequential analysis for the overt GI bleeding in trials with use of PPI and trials with use of H2RA


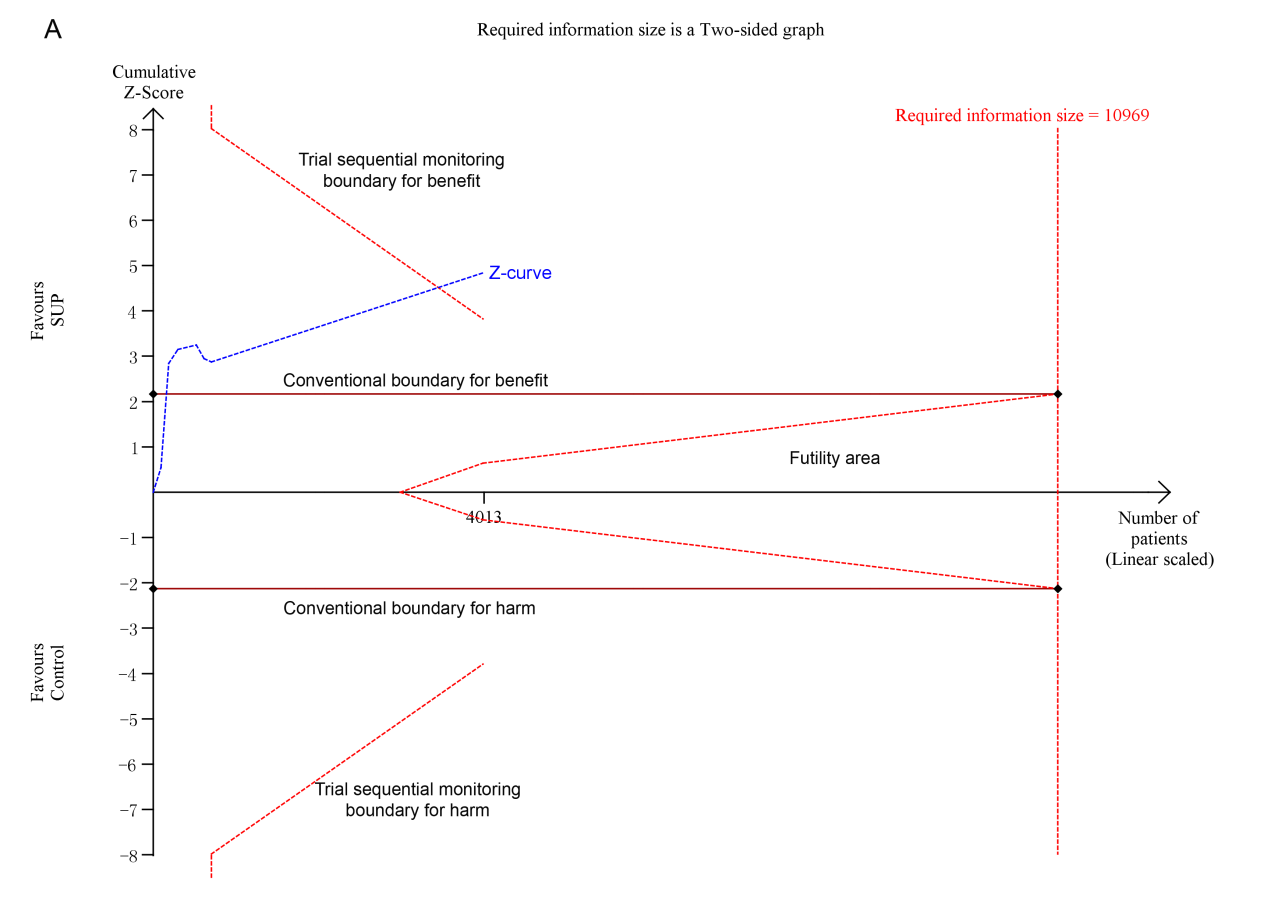


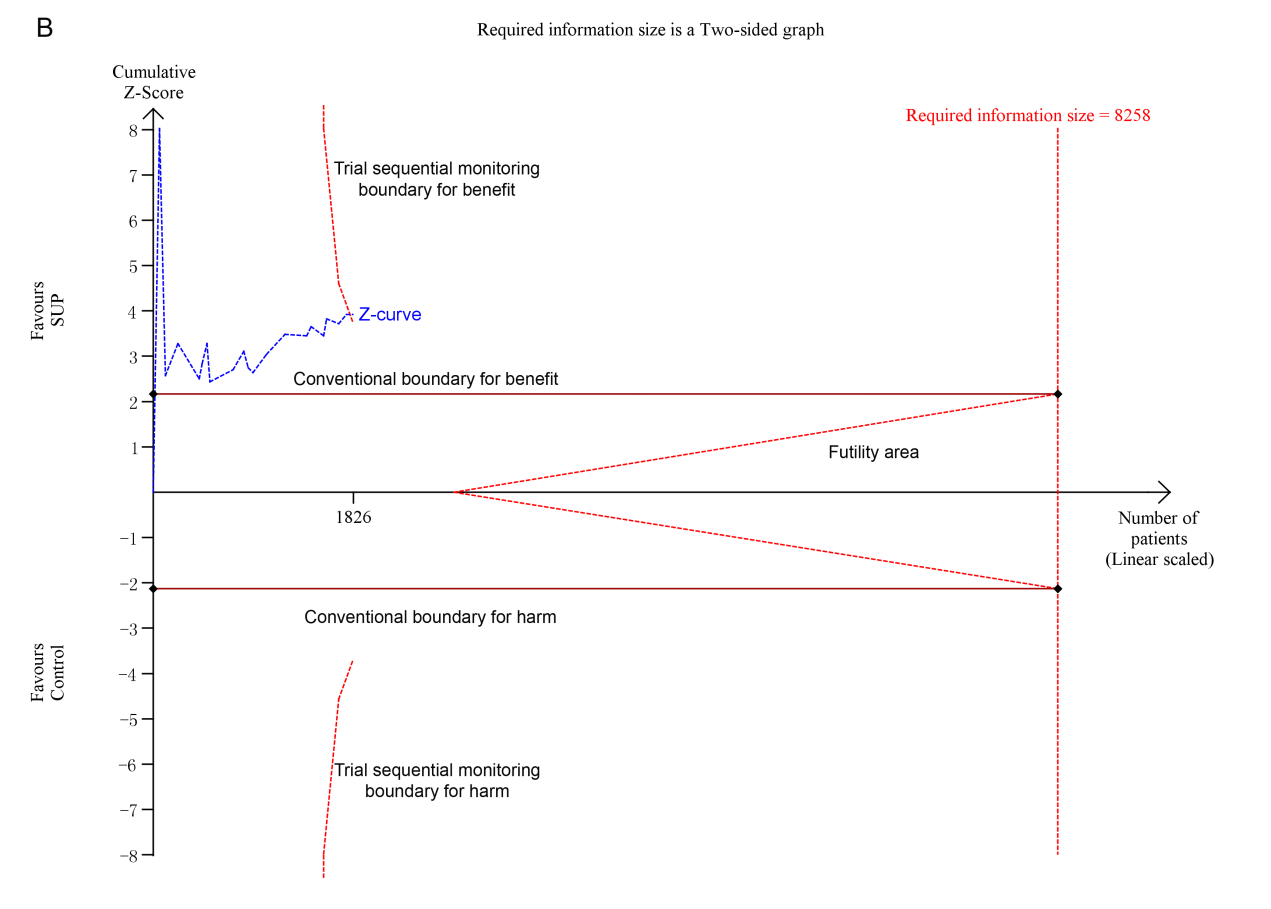


(**Panel** **A**) Trial sequential analysis using random-effects model for the overt GI bleeding in trials with use of PPI, with an adjusted type I error of 3.3%, power of 80%, D^2^ of 25% (the actual measured D^2^ was 0), and a relative risk reduction of 20% in control event proportion of 8.9%. The cumulative z-curve cross the trial sequential monitoring boundary for benefit, but the required information size of 10969 patients are not reached, the TSA-adjusted 95% CI for an RR of 0.57 is 0.36 to 0.88. (**Panel** **B**) Trial sequential analysis using random-effects model for the overt GI bleeding in trials with use of H2RA, with an adjusted type I error of 3.3%, power of 80%, D^2^ of 59%, and a relative risk reduction of 20% in control event proportion of 19.3%. The cumulative z-curve cross the trial sequential monitoring boundary for benefit, but the required information size of 8258 patients are not reached, the TSA-adjusted 95% CI for an RR of 0.45 is 0.26 to 0.79.

## Figure S3. The conventional meta-analysis for the all-cause mortality


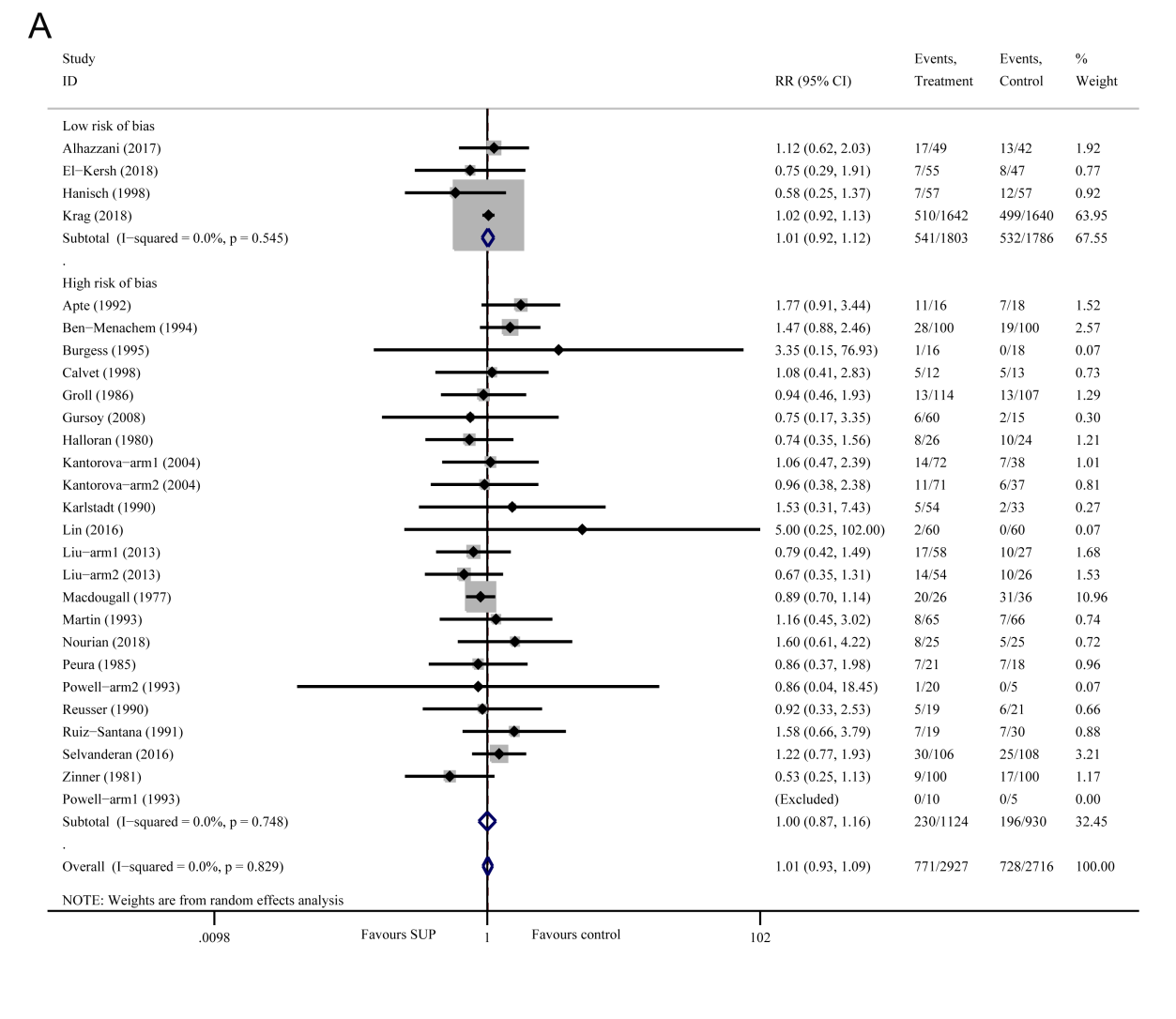


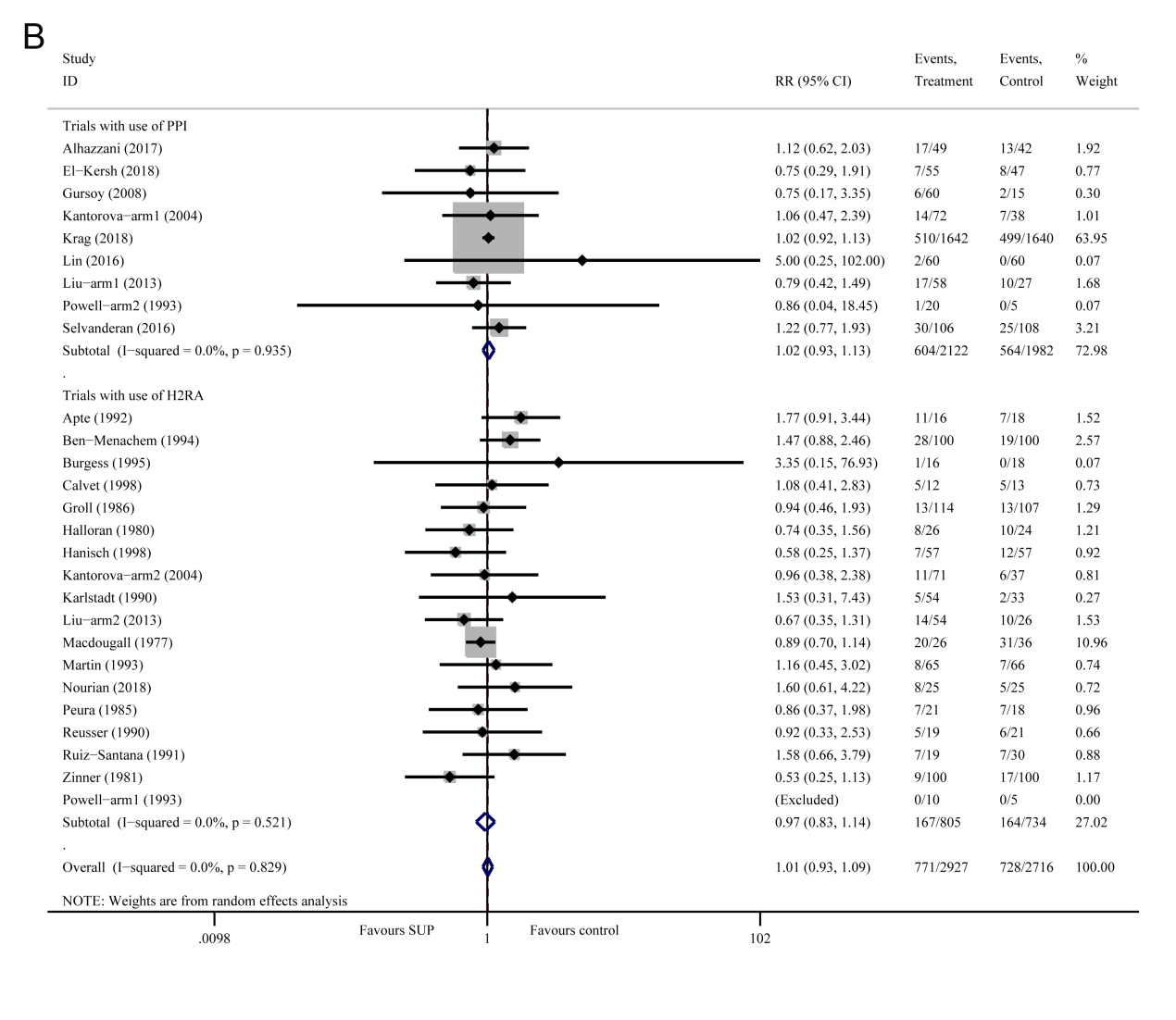


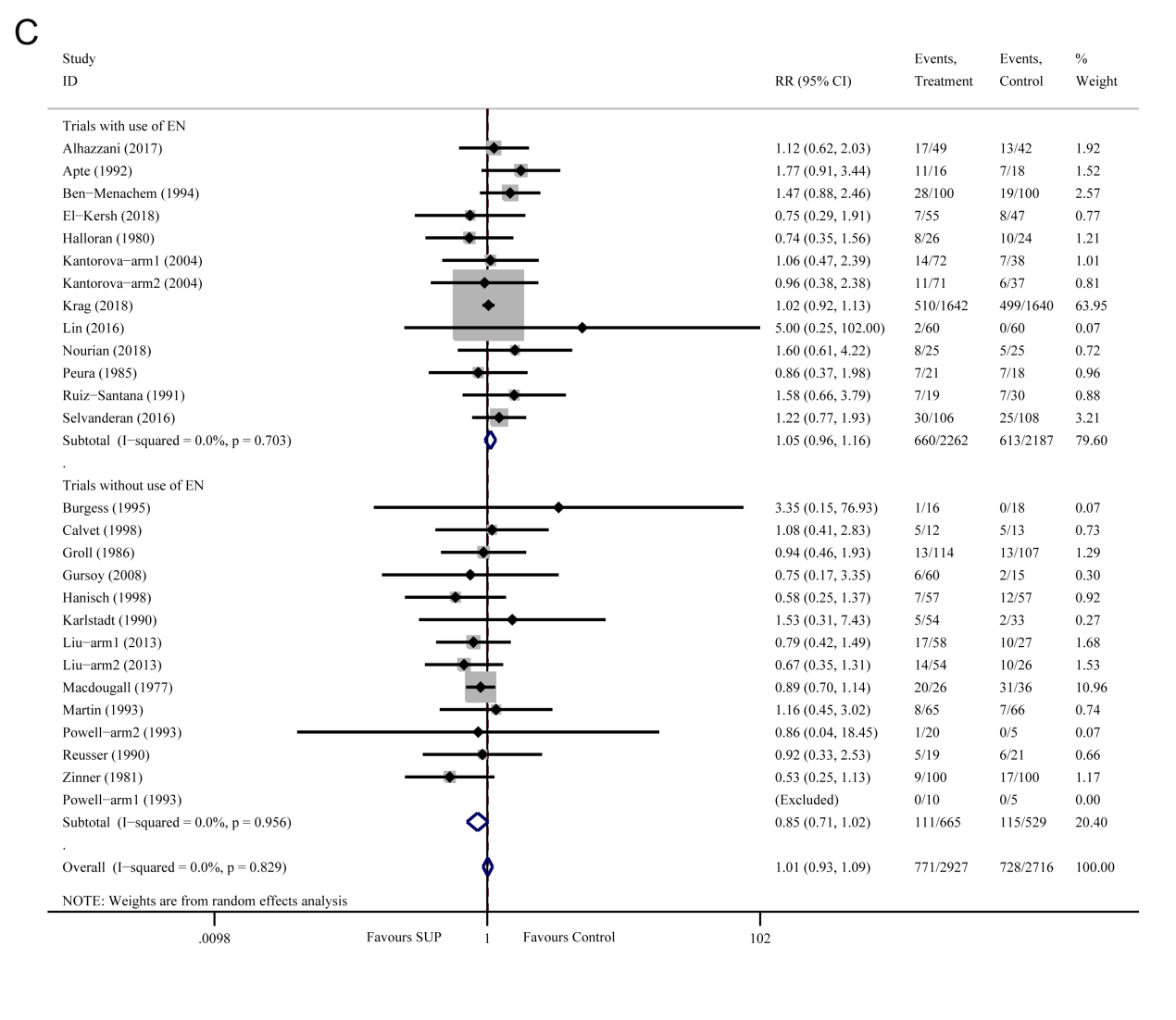


(**Panel** **A**) The conventional meta-analysis of the all-cause mortality in subgroup stratified based on the trials quality. (**Panel** **B**) The conventional meta-analysis of the all-cause mortality in subgroup stratified based on the type of SUP used. (**Panel** **C**) The conventional meta-analysis of the all-cause mortality in subgroup stratified based on whether enteral nutrition was used.

## Figure S4. Trial sequential analysis for the all-cause mortality


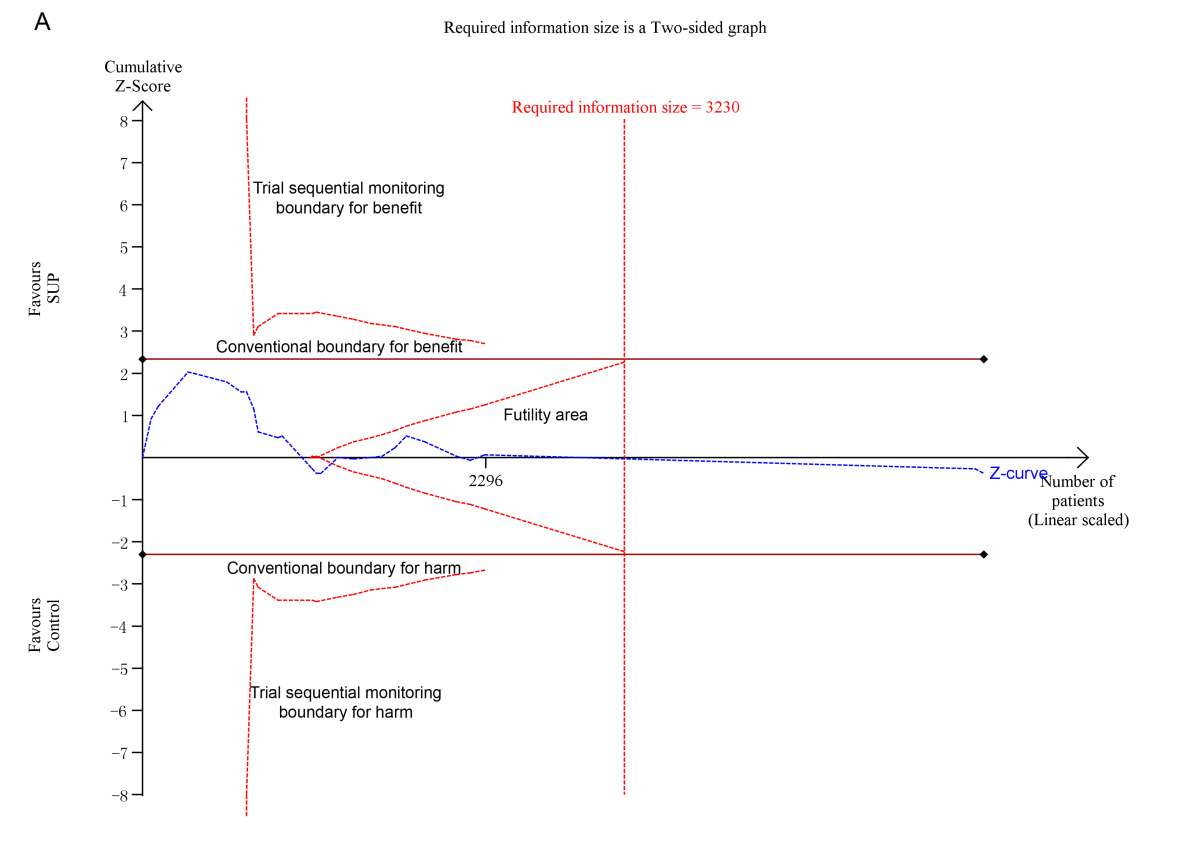


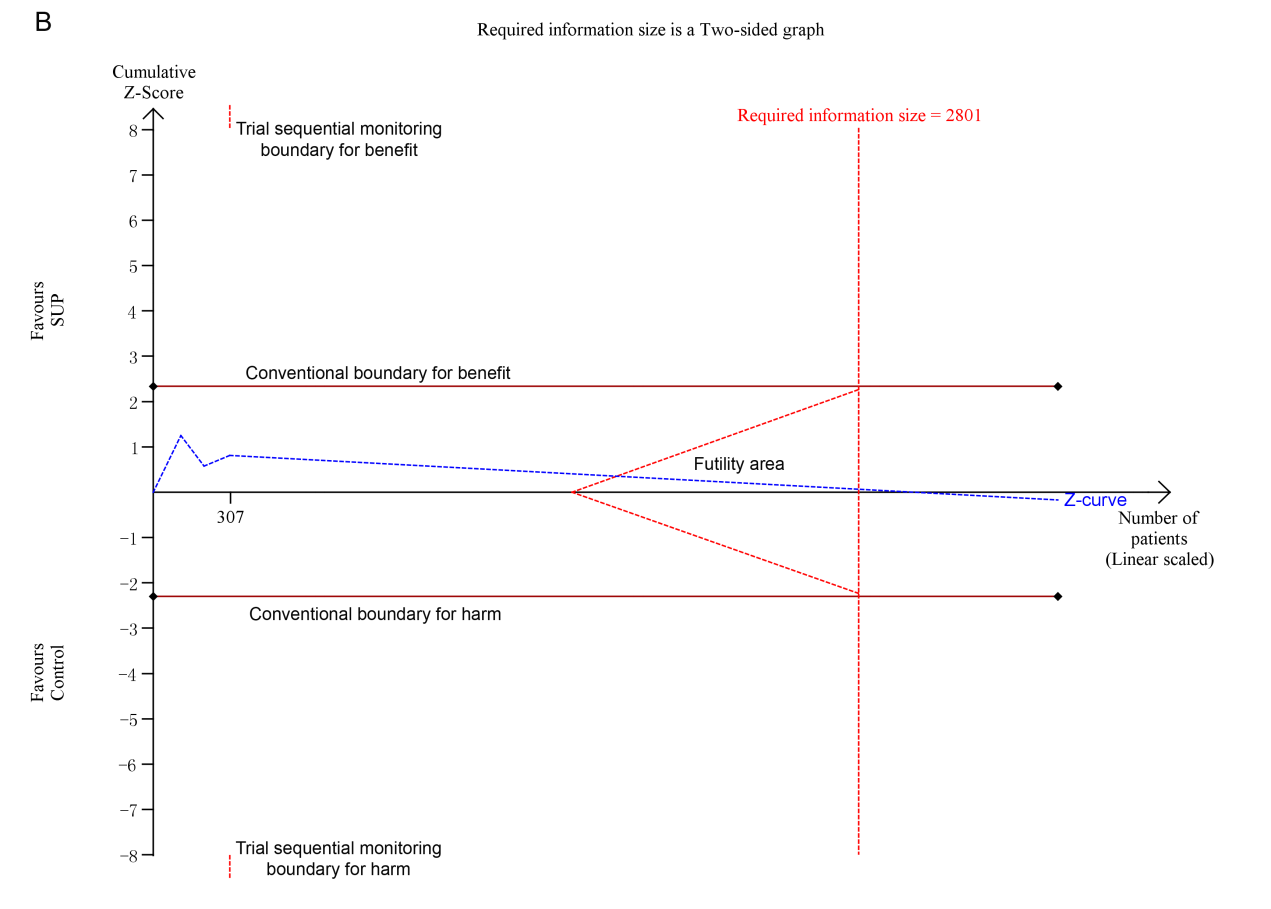


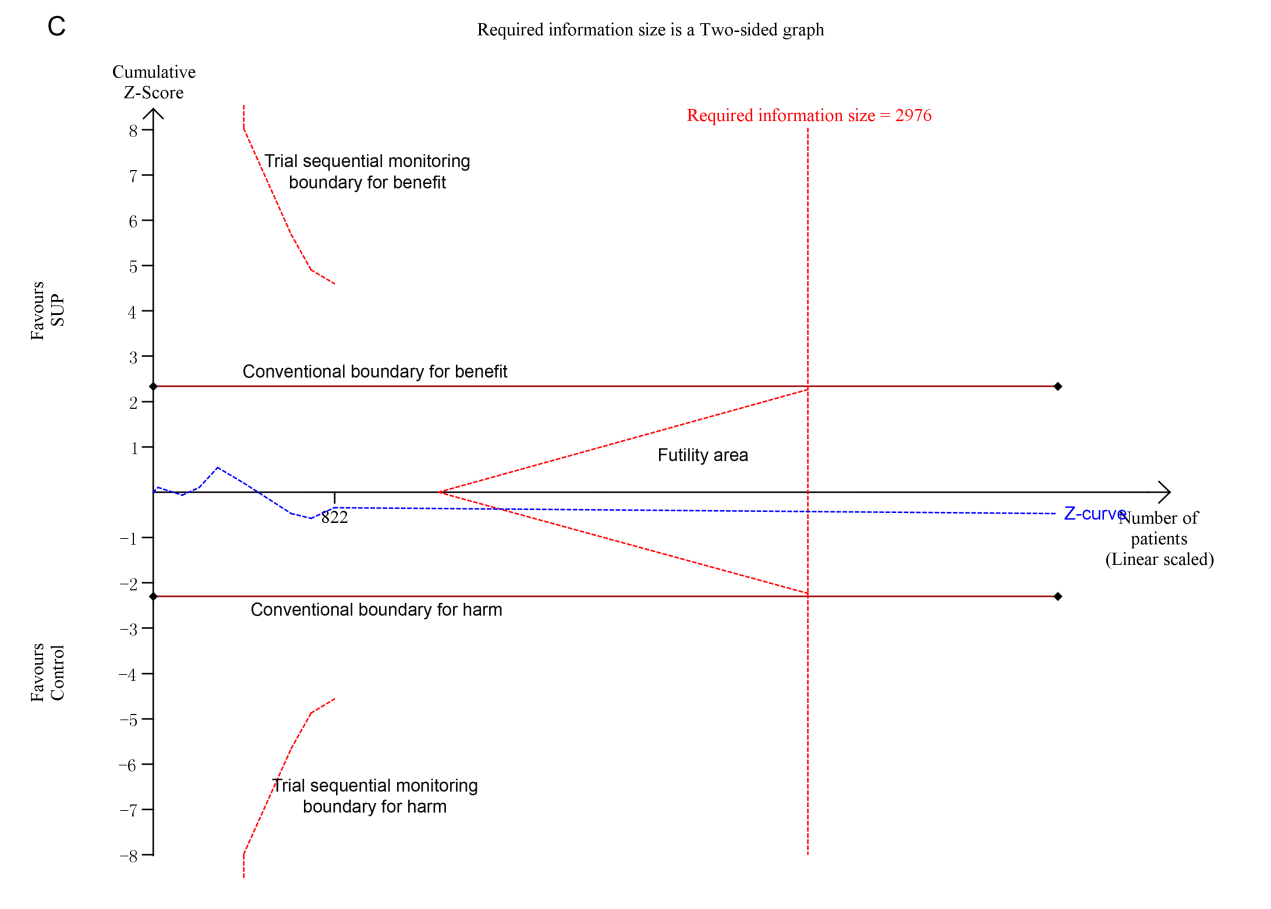


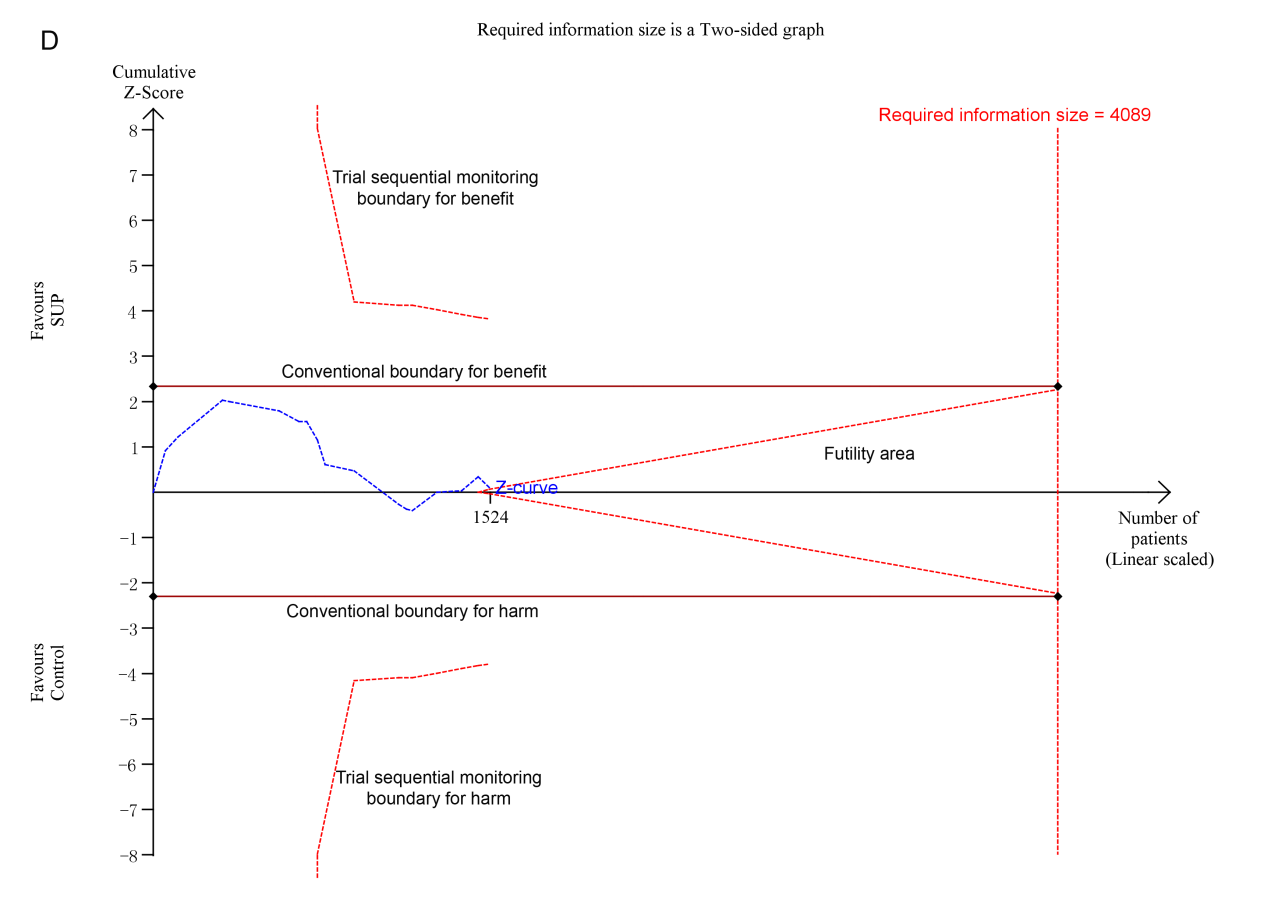


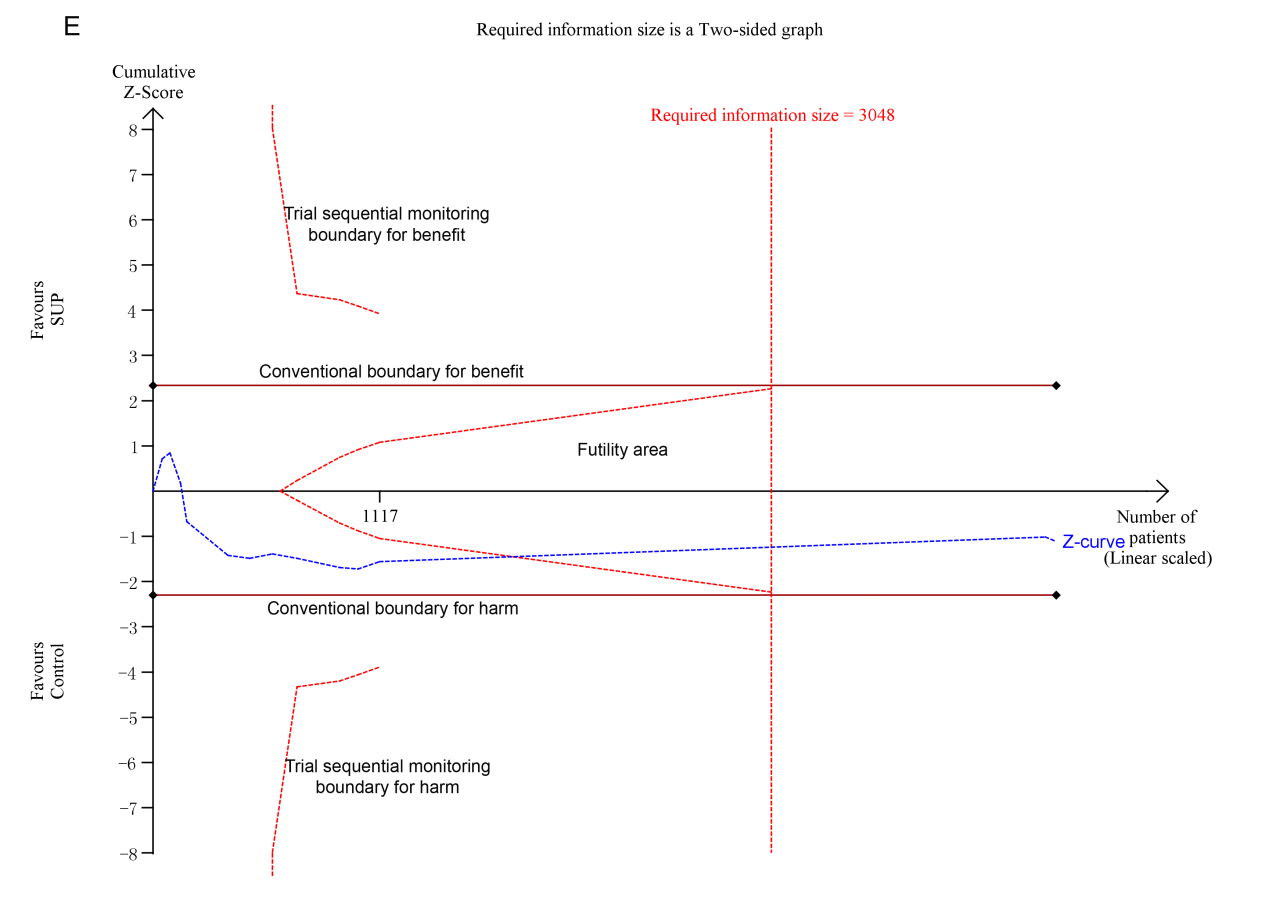


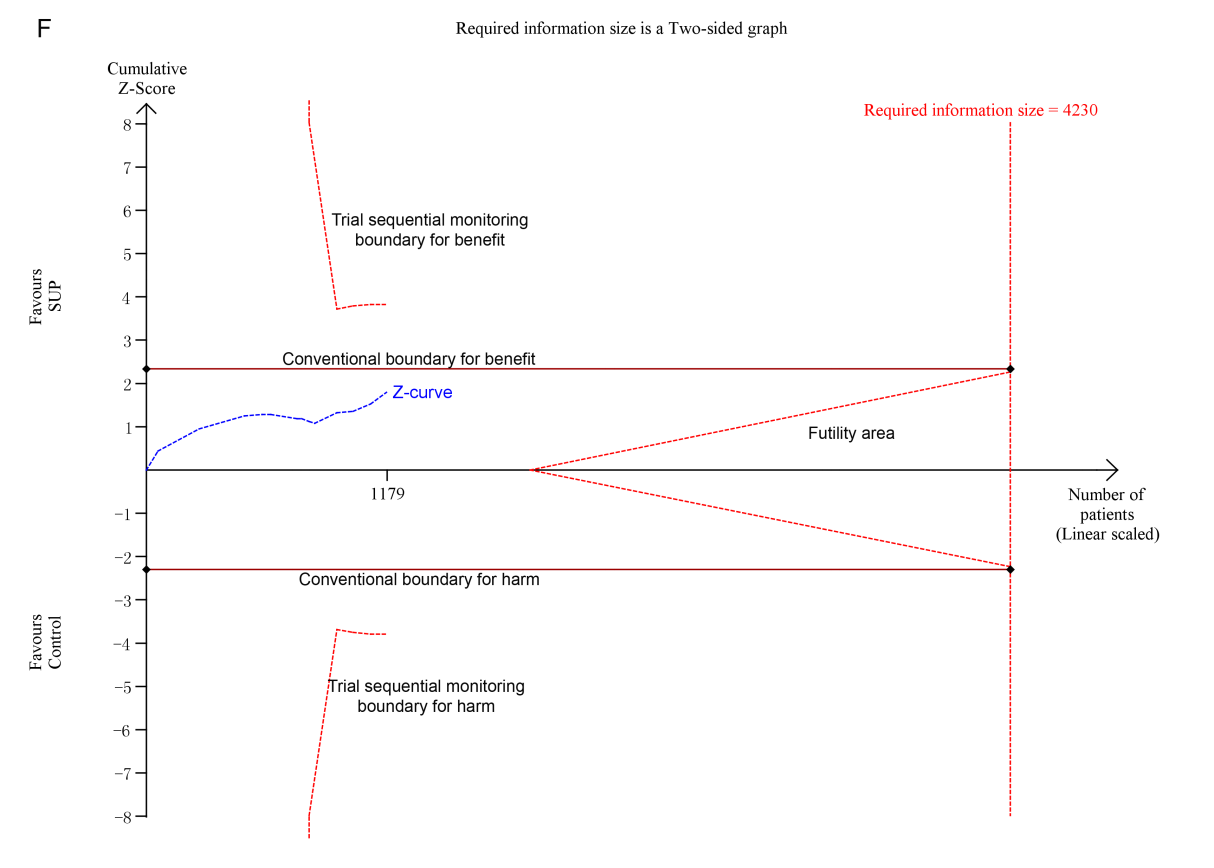


(**Panel** **A**) Trial sequential analysis using random-effects model for the all-cause mortality in all included trials, with an adjusted type I error of 2.5%, power of 80%, D^2^ of 25% (the actual measured D^2^ was 0), and a relative risk reduction of 20% in control event proportion of 26.8%. The cumulative z-curve cross the boundary for futility, and the required information size of 3230 patients are also reached, the TSA-adjusted 95% CI for an RR of 1.01 is 0.90 to 1.13. (**Panel** **B**) Trial sequential analysis using random-effects model for the all-cause mortality in trials with low risk of bias, with an adjusted type I error of 2.5%, power of 80%, D^2^ of 25% (the actual measured D^2^ was 0), and a relative risk reduction of 20% in control event proportion of 29.8%. The cumulative z-curve cross the boundary for futility, and the required information size of 2801 patients are reached, the TSA-adjusted 95% CI for an RR of 1.01 is 0.67 to 1.52. (**Panel** **C**) Trial sequential analysis using random-effects model for the all-cause mortality in trials with use of PPI, with an adjusted type I error of 2.5%, power of 80%, D^2^ of 25% (the actual measured D^2^ was 0), and a relative risk reduction of 20% in control event proportion of 28.5%. The cumulative z-curve cross the boundary for futility, the required information size of 2976 patients are reached, the TSA-adjusted 95% CI for an RR of 1.02 is 0.82 to1.28. (**Panel** **D**) Trial sequential analysis using random-effects model for the all-cause mortality in trials with use of H2RA, with an adjusted type I error of 2.5%, power of 80%, D^2^ of 25% (the actual measured D^2^ was 0), and a relative risk reduction of 20% in control event proportion of 22.3%. The cumulative z-curve cross the boundary for futility, but the required information size of 4089 patients are not reached, the TSA-adjusted 95% CI for an RR of 0.97 is 0.74 to 1.28. (**Panel** **E**) Trial sequential analysis using random-effects model for the all-cause mortality in trials with use of EN, with an adjusted type I error of 2.5%, power of 80%, D^2^ of 25% (the actual measured D^2^ was 0), and a relative risk reduction of 20% in control event proportion of 28%. The cumulative z-curve cross the boundary for futility, and the required information size of 3048 patients are reached, the TSA-adjusted 95% CI for an RR of 1.05 is 0.88 to 1.27. (**Panel** **F**) Trial sequential analysis using random-effects model for the all-cause mortality in trials without use of EN, with an adjusted type I error of 2.5%, power of 80%, D^2^ of 25% (the actual measured D^2^ was 0), and a relative risk reduction of 20% in control event proportion of 21.7%. The cumulative z-curve cross no boundaries, and the required information size of 4230 patients are not reached, the TSA-adjusted 95% CI for an RR of 0.85 is 0.63 to1.15.

## Figure S5. Trial sequential analysis for the incident of pneumonia


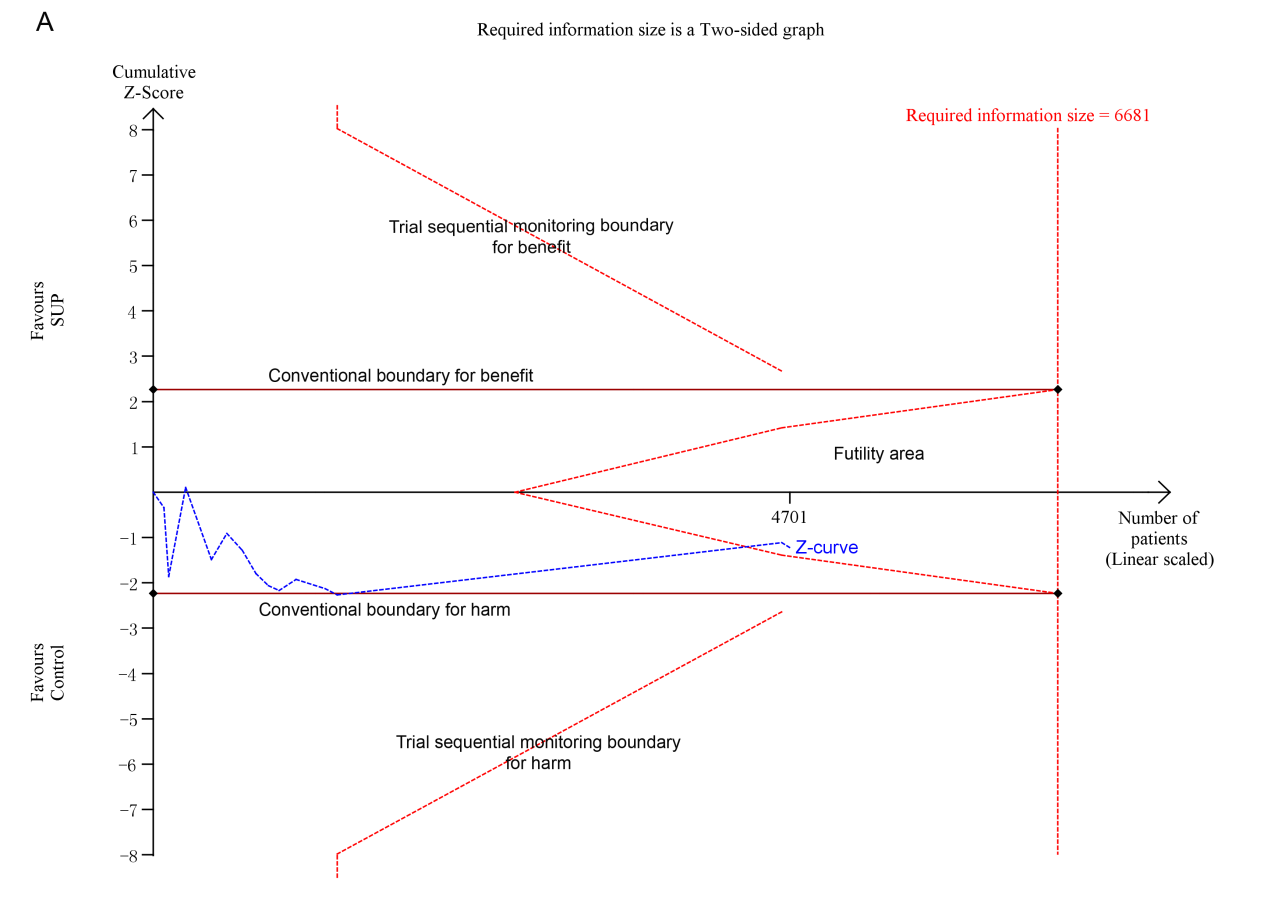


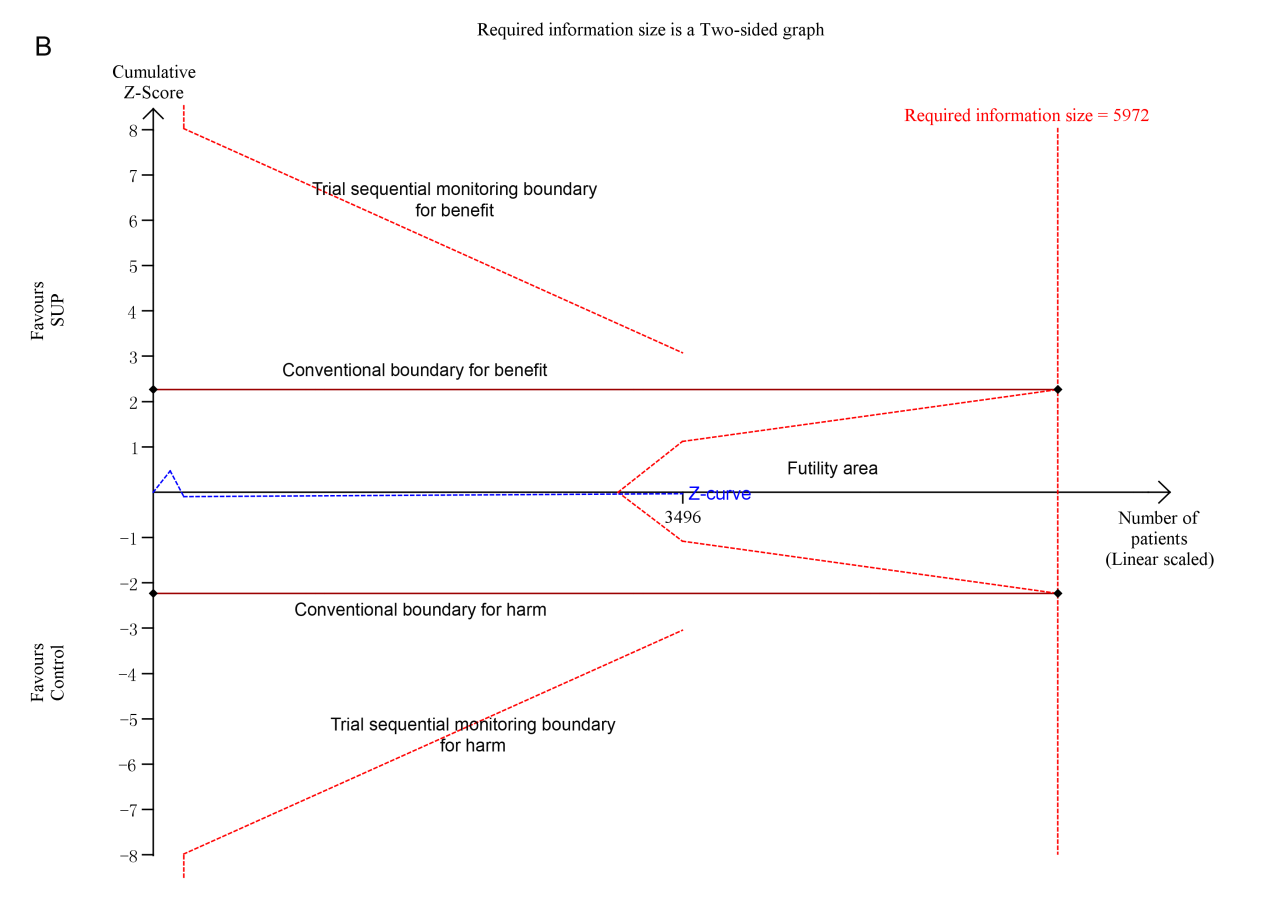


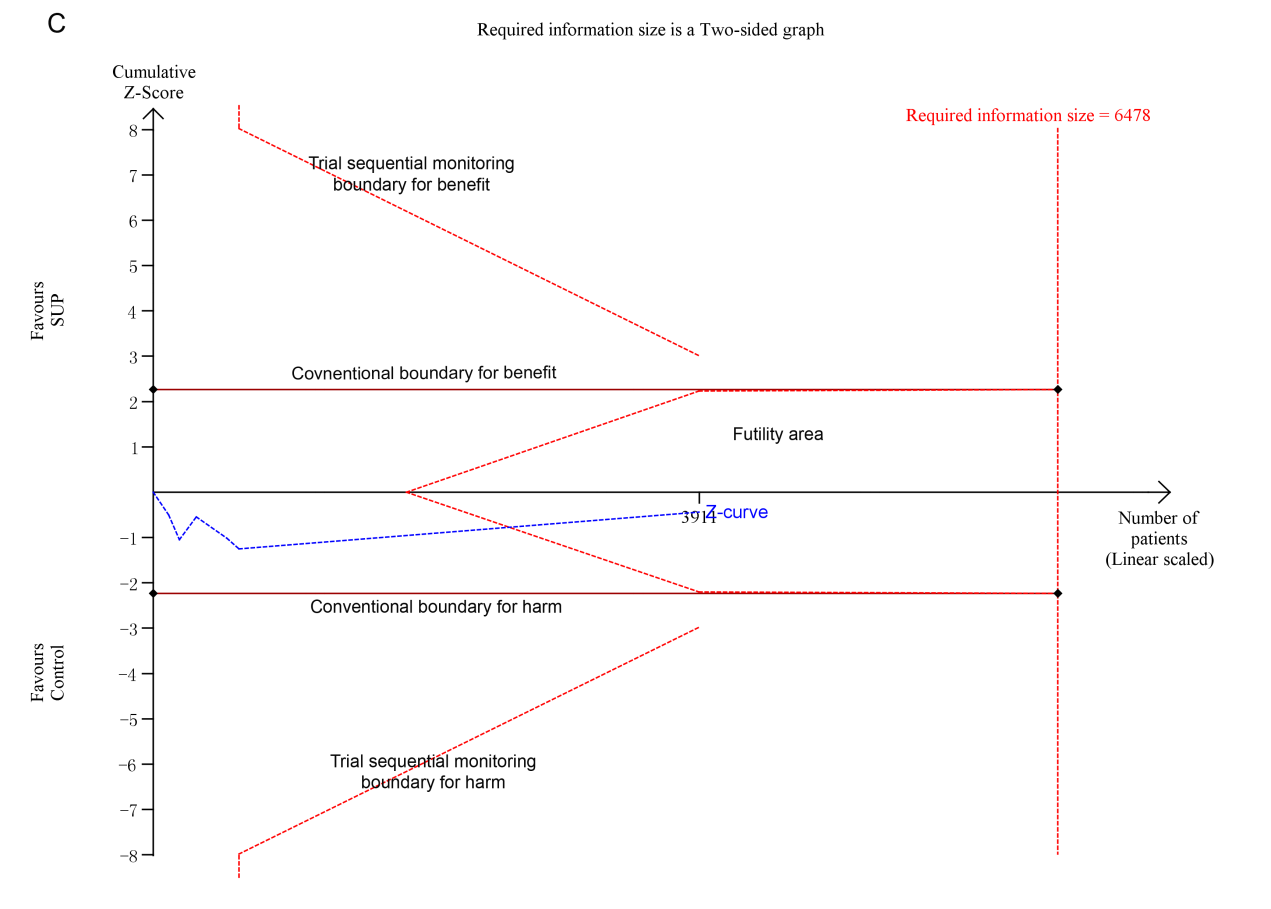


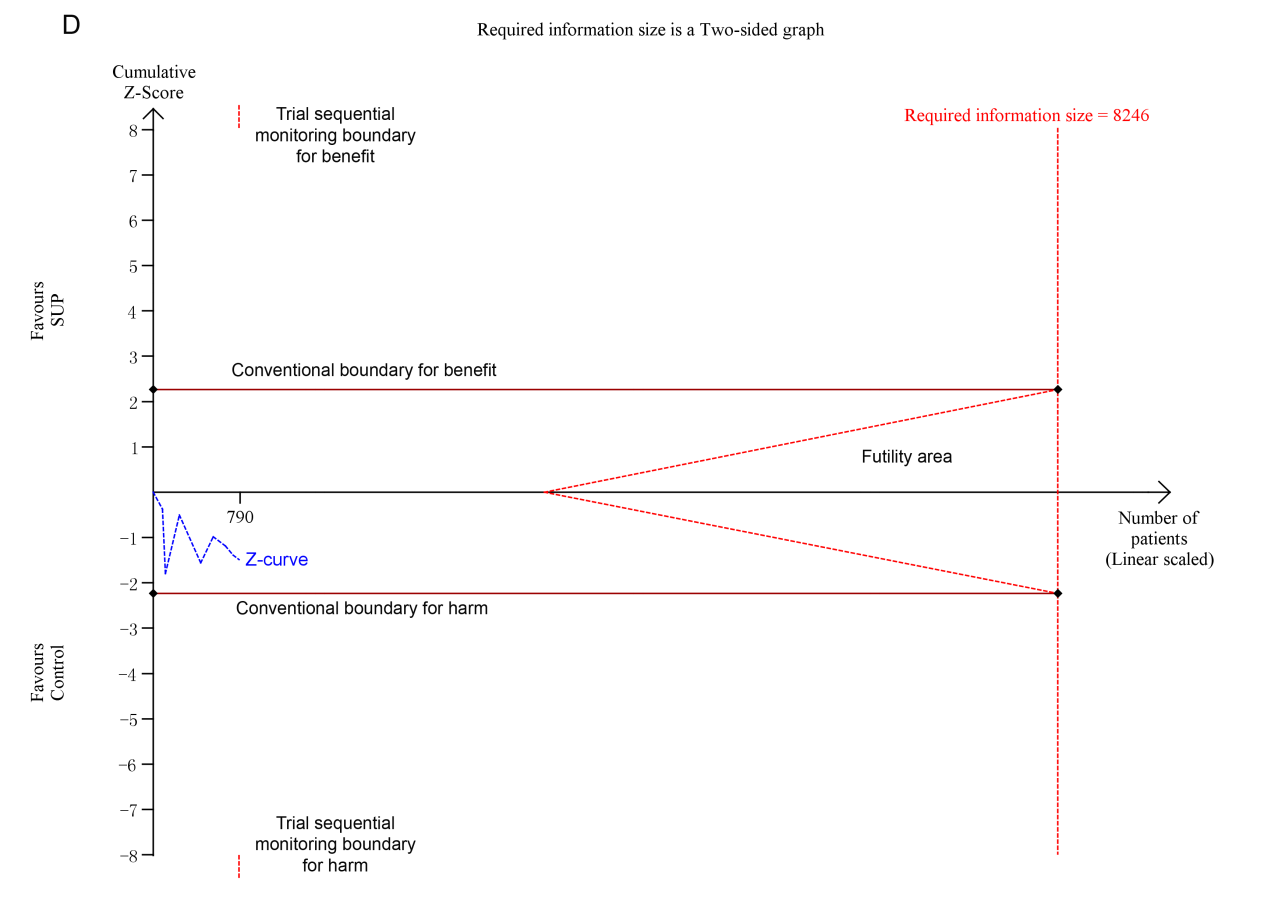


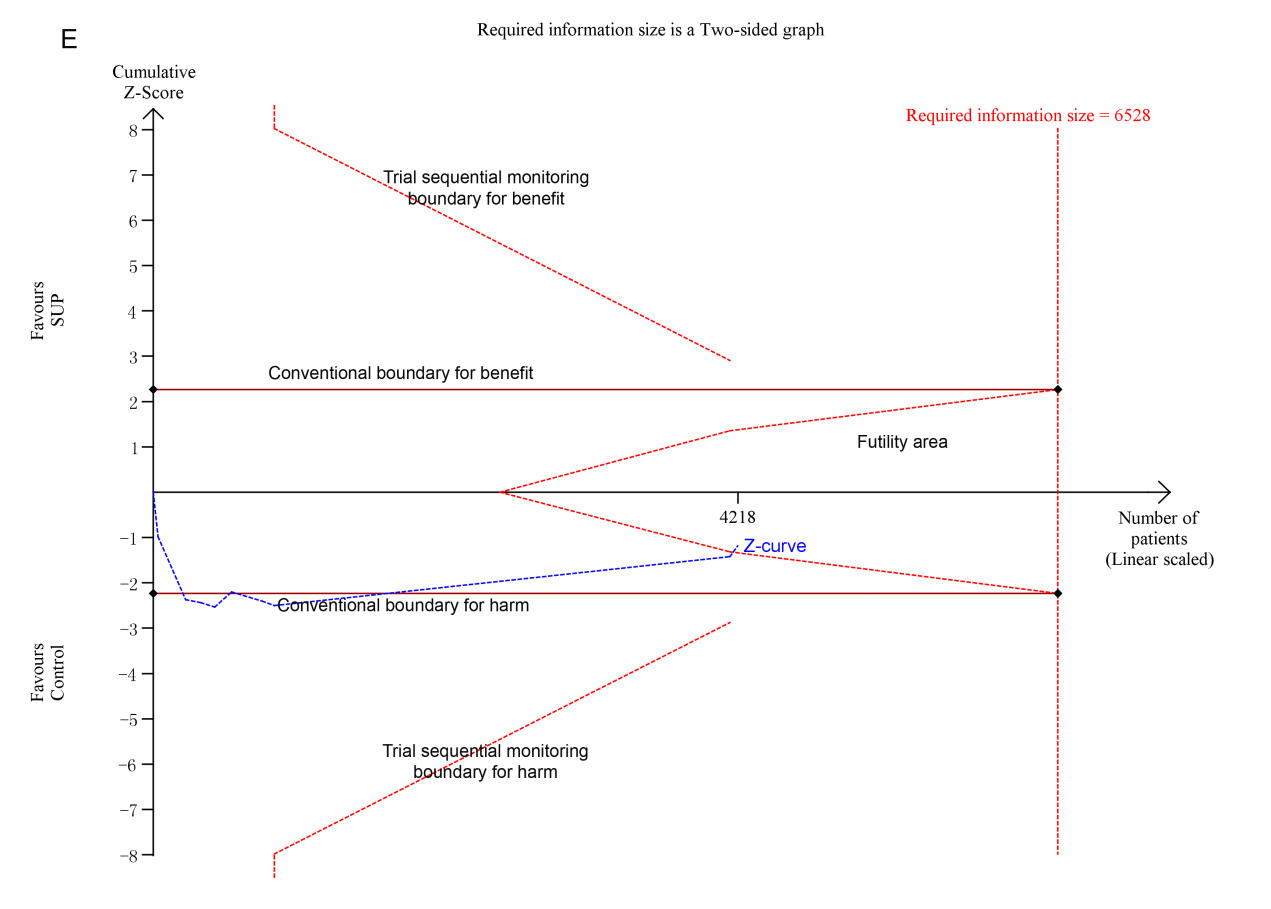


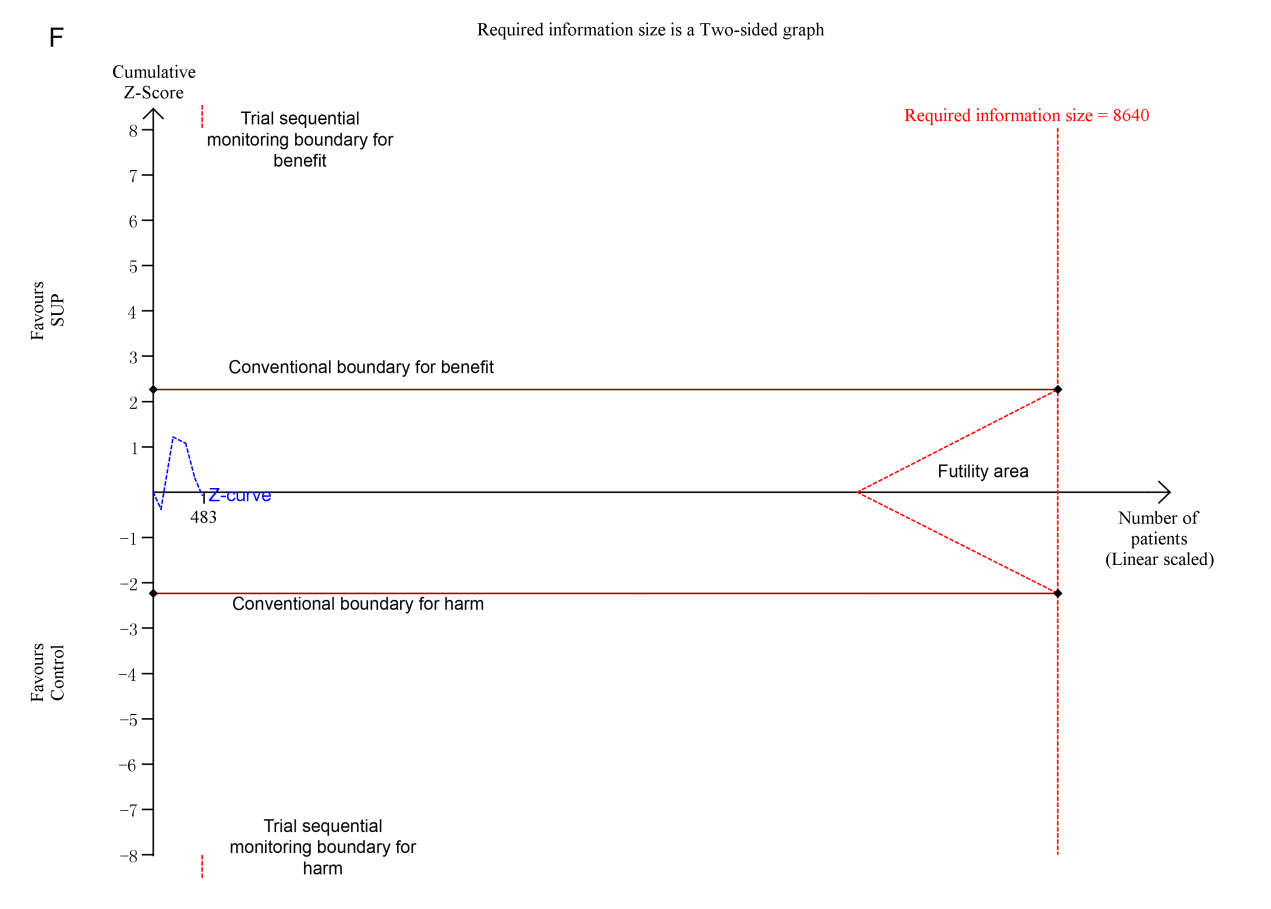


(**Panel** **A**) Trial sequential analysis using random-effects model for the incident of pneumonia in all included trials, with an adjusted type I error of 2.5%, power of 80%, D^2^ of 25% (the actual measured D^2^ was 0), and a relative risk reduction of 20% in control event proportion of 14.8%. The cumulative z-curve cross the boundary for futility, and the required information size of 6681 patients are not reached, the TSA-adjusted 95% CI for an RR of 1.09 is 0.90 to1.31. (**Panel** **B**) Trial sequential analysis using random-effects model for the incident of pneumonia in trials with low risk of bias, with an adjusted type I error of 2.5%, power of 80%, D^2^ of 25% (the actual measured D^2^ was 0), and a relative risk reduction of 20% in control event proportion of 16.3%. The cumulative z-curve cross the boundary for futility, the required information size of 5972 patients are not reached, the TSA-adjusted 95% CI for an RR of 1.00 is 0.79 to1.27. (**Panel** **C**) Trial sequential analysis using random-effects model for the incident of pneumonia in trials with use of PPI, with an adjusted type I error of 2.5%, power of 80%, D^2^ of 25% (the actual measured D^2^ was 0), and a relative risk reduction of 20% in control event proportion of 15.2%. The cumulative z-curve cross the boundary for futility, the required information size of 6478 patients are not reached, the TSA-adjusted 95% CI for an RR of 1.03 is 0.82 to 1.29. (**Panel** **D**) Trial sequential analysis using random-effects model for the incident of pneumonia in trials with use of H2RA, with an adjusted type I error of 2.5%, power of 80%, D^2^ of 25% (the actual measured D^2^ was 0), and a relative risk reduction of 20% in control event proportion of 12.3%. The cumulative z-curve cross no boundaries, the required information size of 8246 patients are not reached, the TSA-adjusted 95% CI for an RR of 1.39 is 0.37 to 5.13. (**Panel** **E**) Trial sequential analysis using random-effects model for the incident of pneumonia in trials with use of EN, with an adjusted type I error of 2.5%, power of 80%, D^2^ of 25% (the actual measured D^2^ was 0), and a relative risk reduction of 20% in control event proportion of 15.1%. The cumulative z-curve cross the boundary for futility, but the required information size of 6528 patients are not reached, the TSA-adjusted 95% CI for an RR of 1.09 is 0.88 to 1.34. (**Panel** **F**) Trial sequential analysis using random-effects model for the incident of pneumonia in trials without use of EN, with an adjusted type I error of 2.5%, power of 80%, D^2^ of 25% (the actual measured D^2^ was 0), and a relative risk reduction of 20% in control event proportion of 11.8%. The cumulative z-curve cross no boundaries, and the required information size of 8640 patients are not reached, the TSA-adjusted 95% CI for an RR of 1.08 is 0.14 to 8.58.

## Figure S6. The conventional meta-analysis for the incident of pneumonia


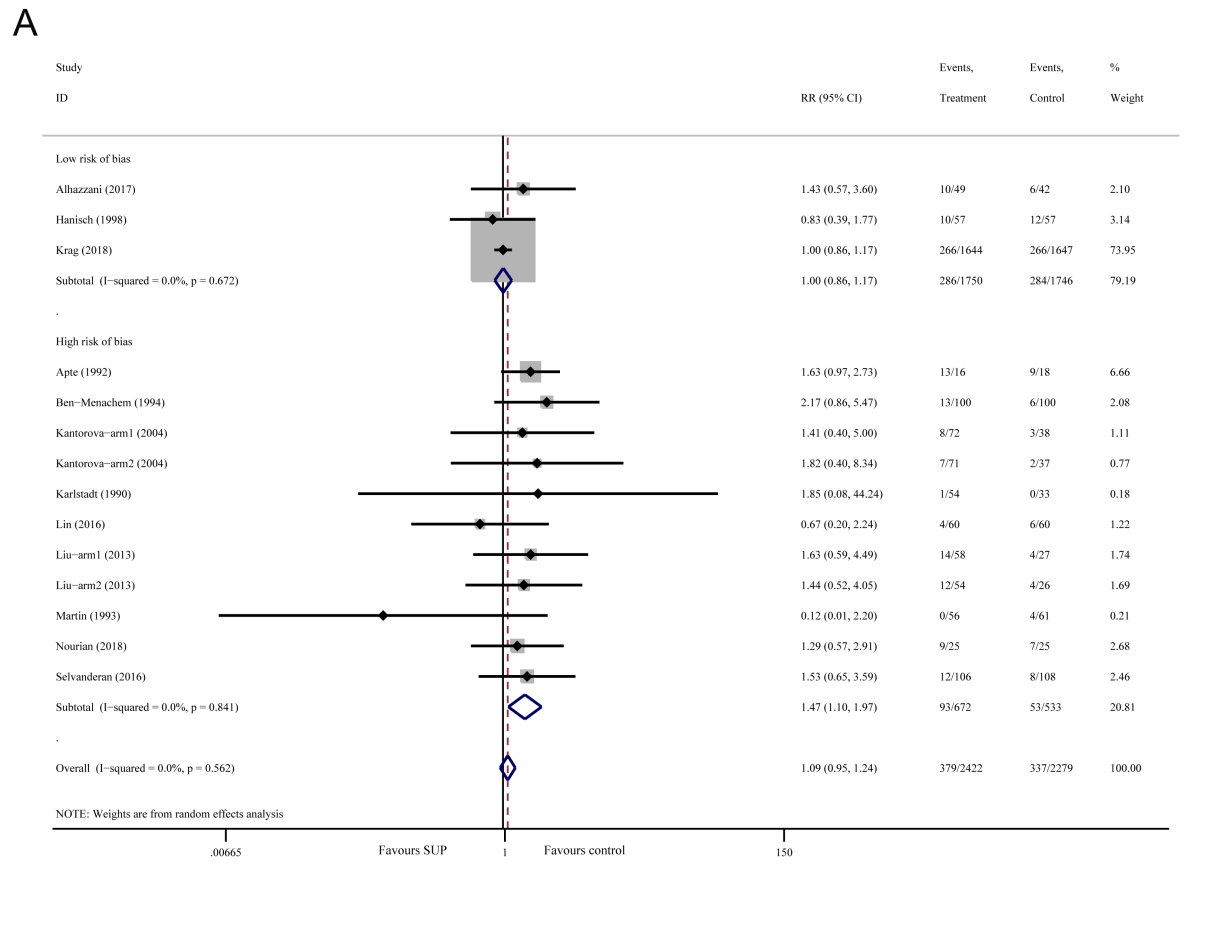


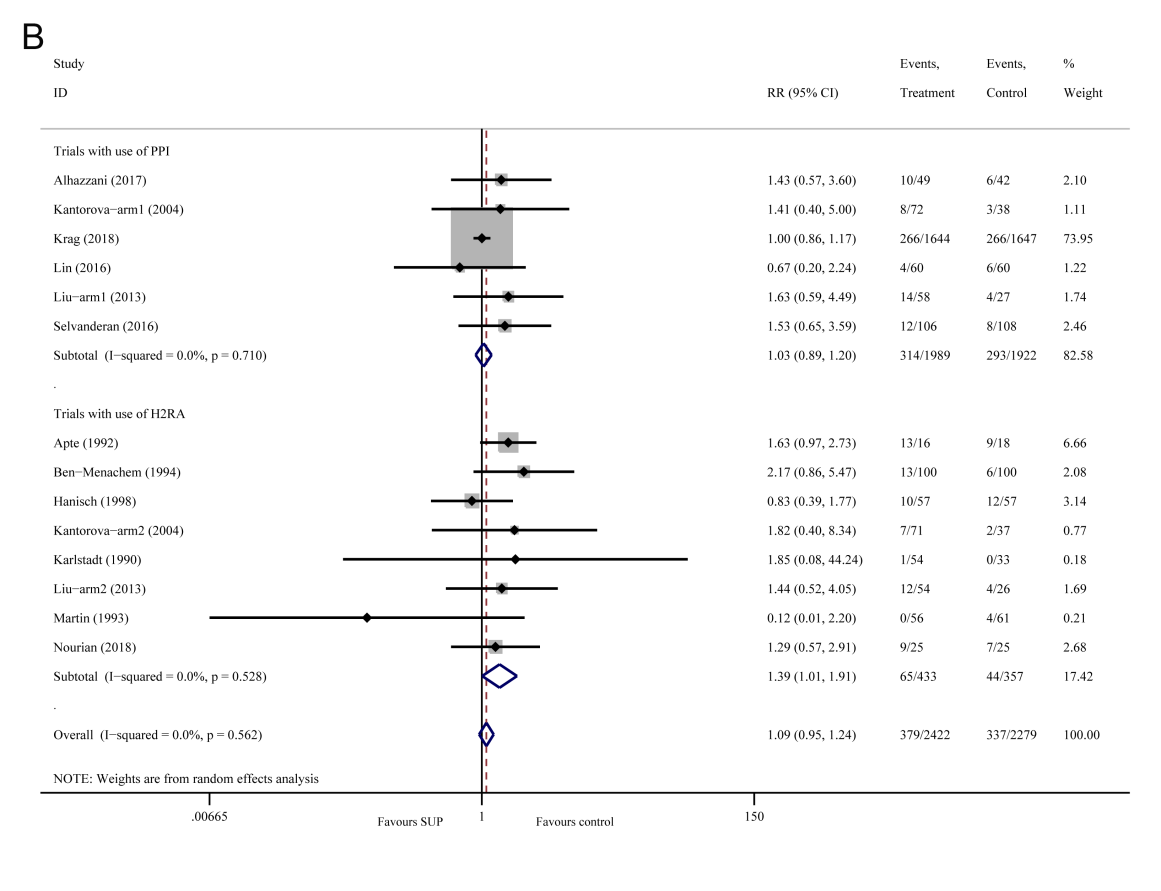


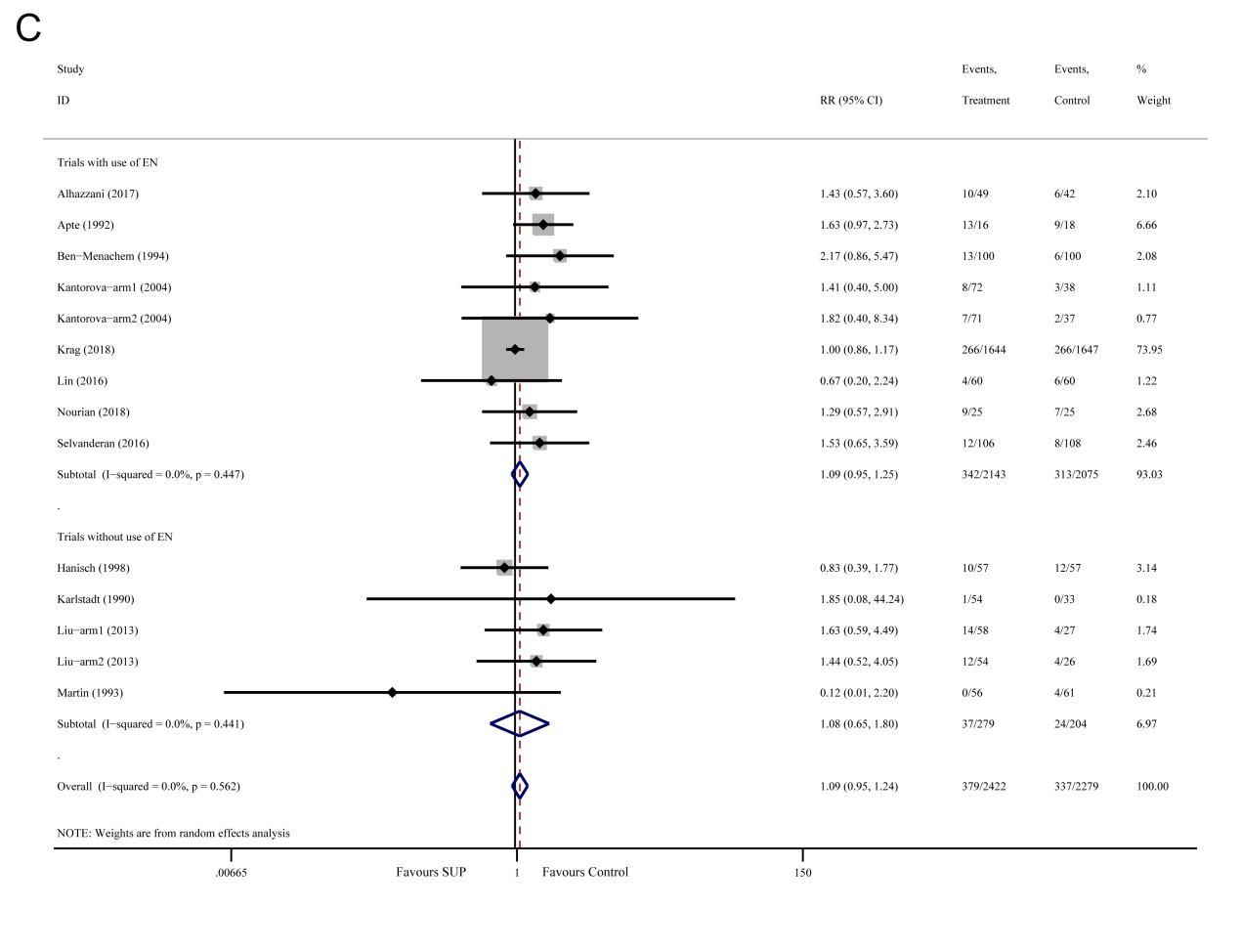


(**Panel** **A**) The conventional meta-analysis of the incident of pneumonia in subgroup stratified based on the trials quality. (**Panel** **B**) The conventional meta-analysis of the incident of pneumonia in subgroup stratified based on the type of SUP used. (**Panel** **C**) The conventional meta-analysis of the incident of pneumonia in subgroup stratified based on whether enteral nutrition was used.

## Figure S7. Sub-analysis of trials based on the trials quality for the incident of clostridium difficile infection


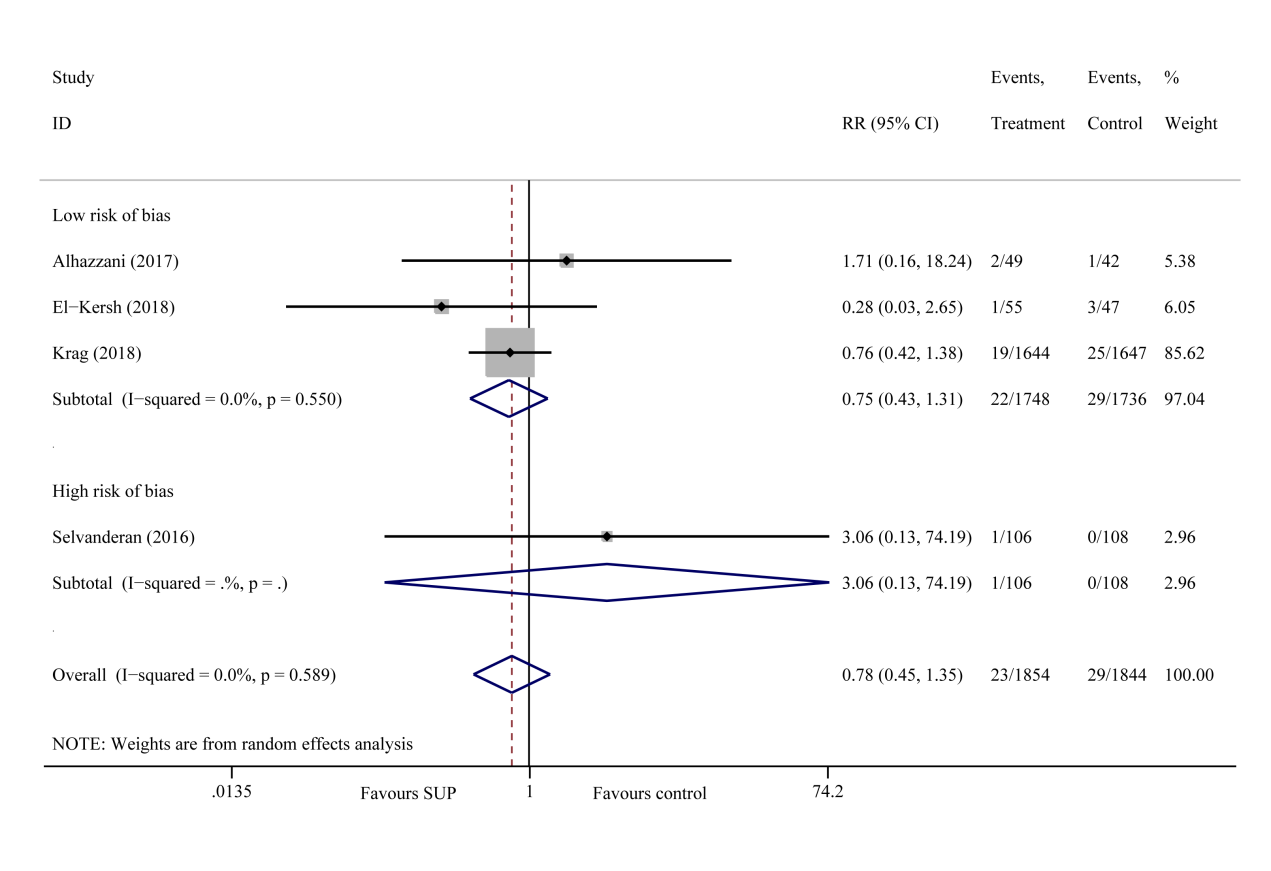


## Figure S8. Trial sequential analysis for the incident of clostridium difficile infection


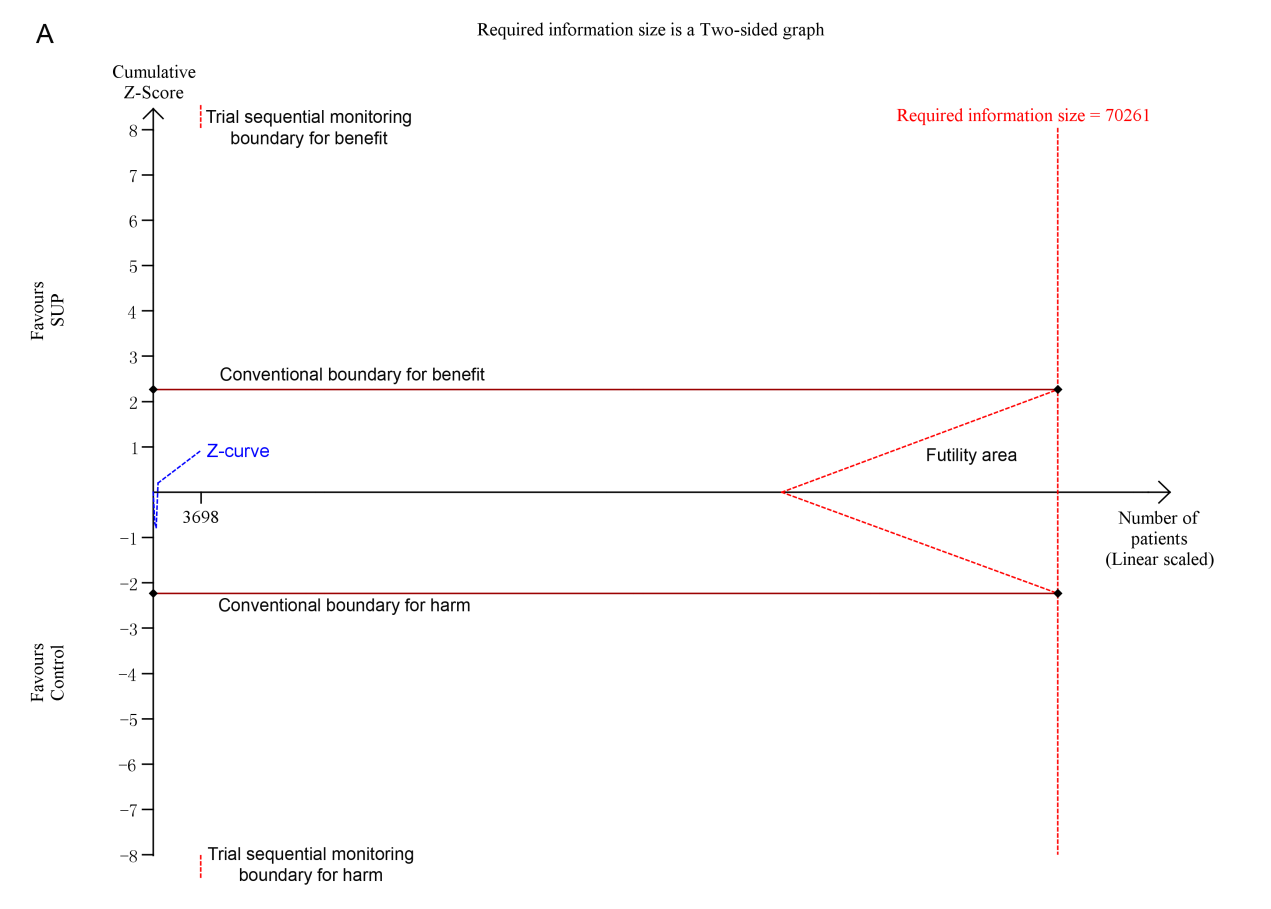


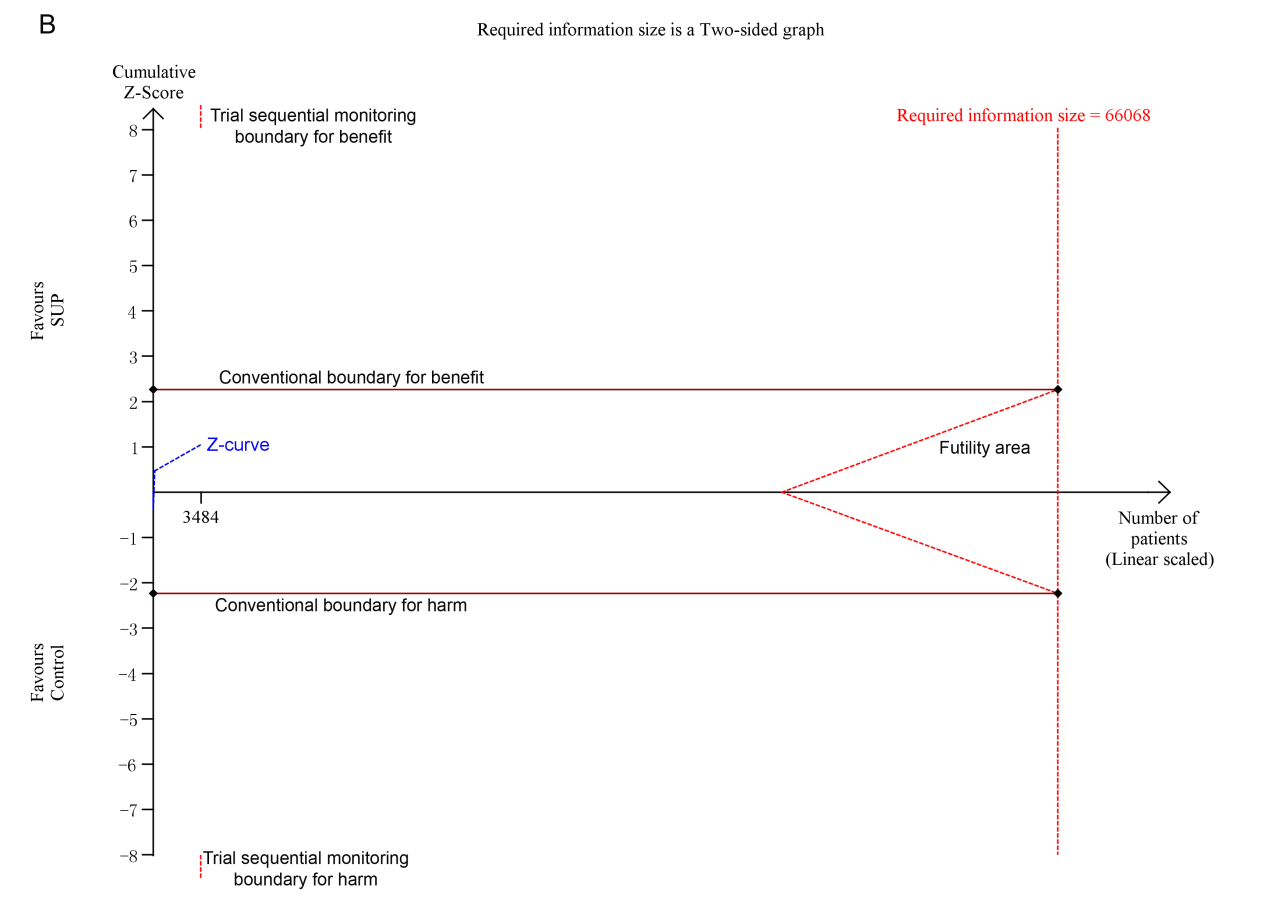


(**Panel** **A**) Trial sequential analysis using random-effects model for the incident of clostridium difficile infection in all included trials, with an adjusted type I error of 2.5%, power of 80%, D^2^ of 25% (the actual measured D^2^ was 0), and a relative risk reduction of 20% in control event proportion of 1.6%. The cumulative z-curve cross no boundaries, and the required information size of 70261 patients are not reached, the TSA-adjusted 95% CI for an RR of 0.78 is 0.08 to 7.32. (**Panel** **B**) Trial sequential analysis using random-effects model for the incident of clostridium difficile infection in trials with low risk of bias, with an adjusted type I error of 2.5%, power of 80%, D^2^ of 25% (the actual measured D^2^ was 0), and a relative risk reduction of 20% in control event proportion of 1.7%. The cumulative z-curve cross no boundaries, and the required information size of 66068 patients are not reached, the TSA-adjusted 95% CI for an RR of 0.75 is 0.08 to 7.26.

## Figure S9. The conventional meta-analysis for the duration of ICU stay


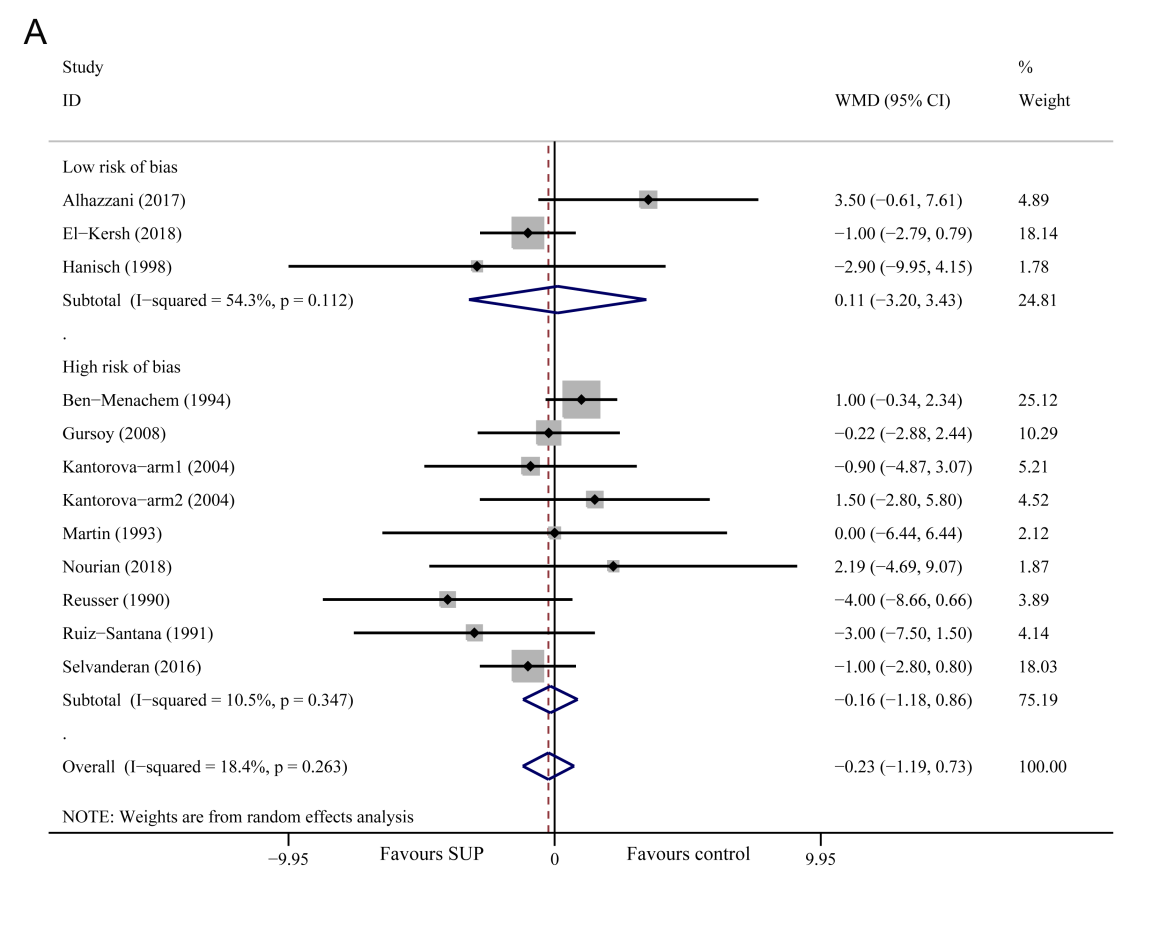


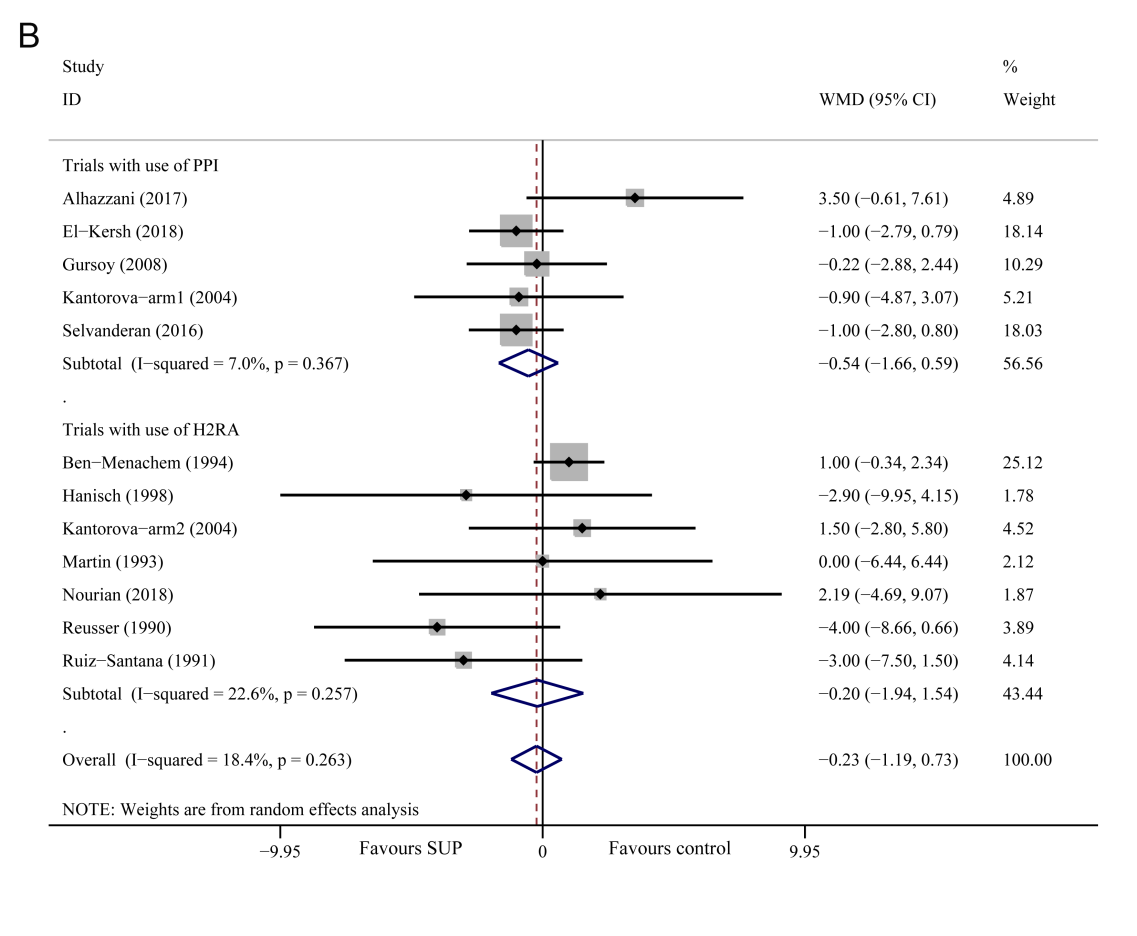


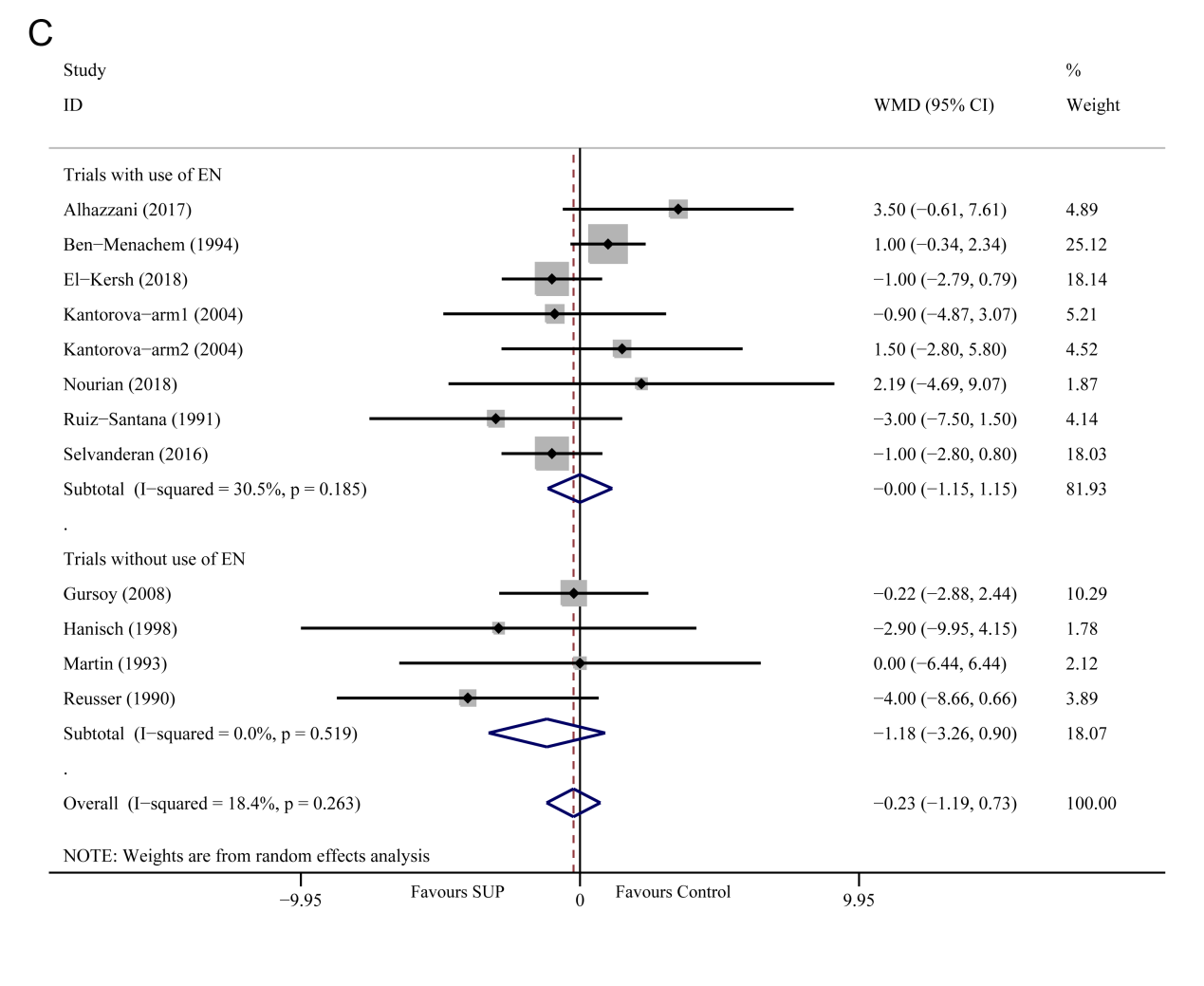


(**Panel** **A**) The conventional meta-analysis of the duration of ICU stay in subgroup stratified based on the trials quality. (**Panel** **B**) The conventional meta-analysis of the duration of ICU stay in subgroup stratified based on the type of SUP used. (**Panel** **C**) The conventional meta-analysis of the duration of ICU stay in subgroup stratified based on whether enteral nutrition was used.

## Figure S10. The conventional meta-analysis for the duration of mechanical ventilation


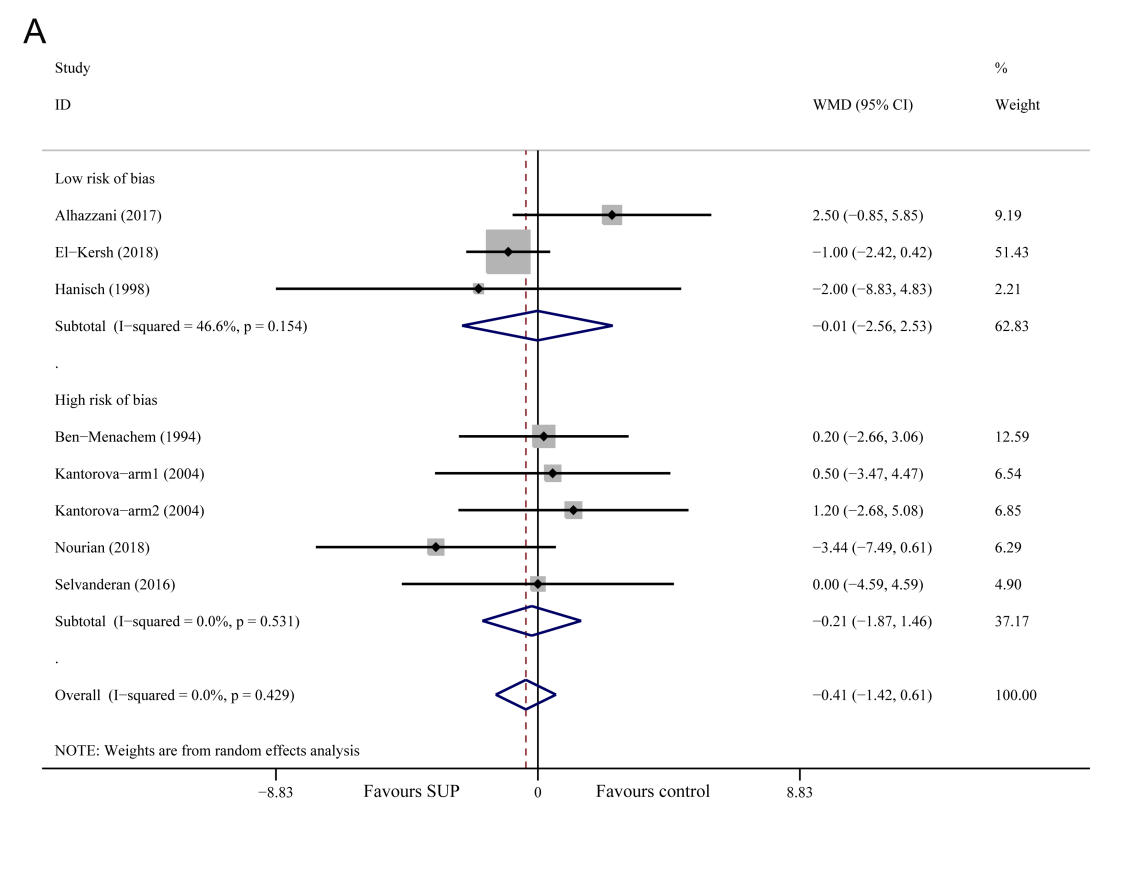


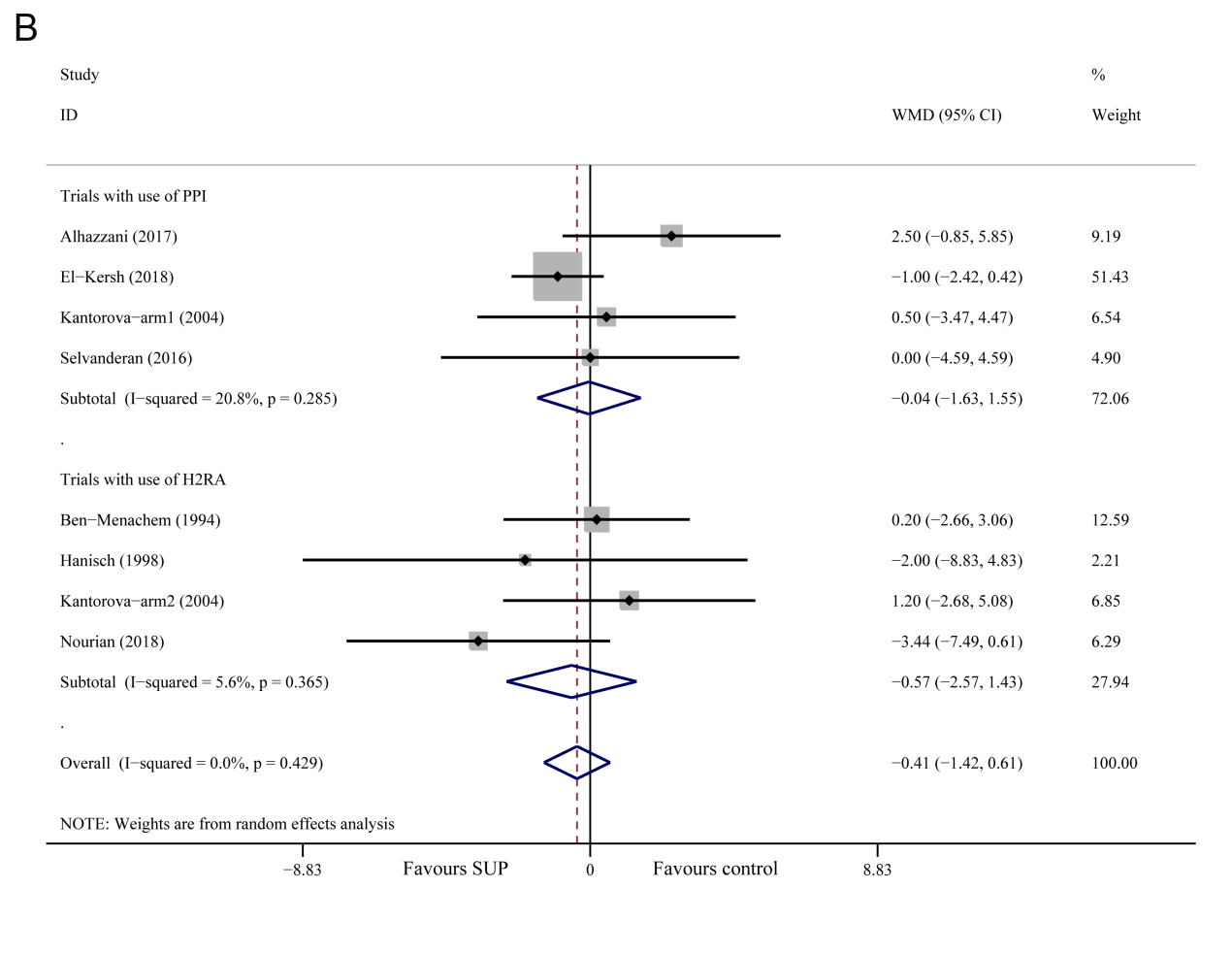


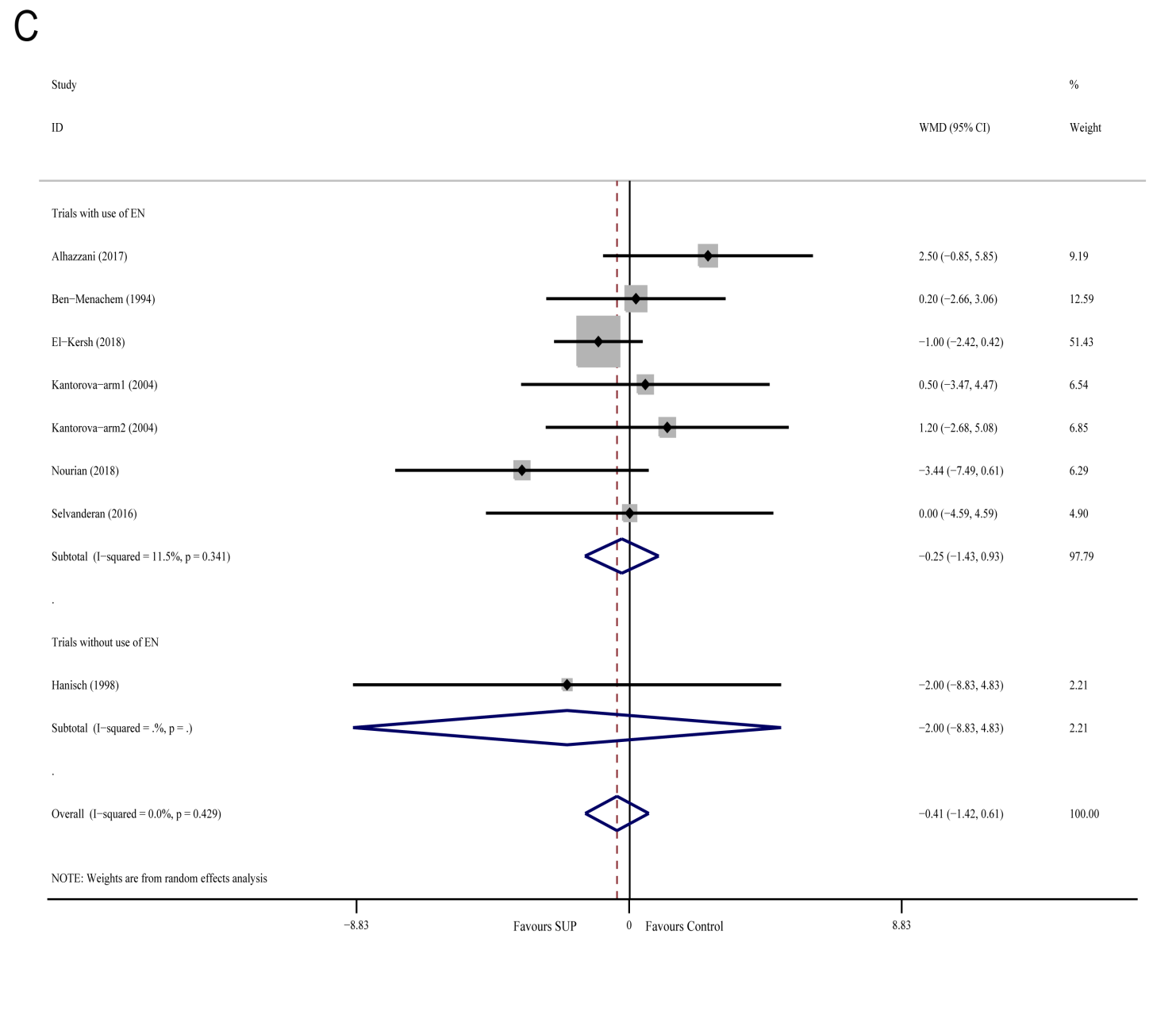


(**Panel** **A**) The conventional meta-analysis of the duration of mechanical ventilation in subgroup stratified based on the trials quality. (**Panel** **B**) The conventional meta-analysis of the duration of mechanical ventilation in subgroup stratified based on the type of SUP used. (**Panel** **C**) The conventional meta-analysis of the duration of mechanical ventilation in subgroup stratified based on whether enteral nutrition was used.
